# Supplementary material for: The epilepsy-associated protein PCDH19 undergoes NMDA receptor-dependent proteolytic cleavage and regulates the expression of immediate-early genes
Source: Cell Rep. 2022 May 24;39(8):110857. doi: 10.1016/j.celrep.2022.110857 (PMC9152703; doi:10.1016/j.celrep.2022.110857)
Supplement: Document S1. Figures S1–S5 and Tables S1–S9 [file mmc1.pdf]

Cell Reports, Volume *39*

Supplemental information

The epilepsy-associated protein PCDH19 undergoes NMDA receptor-dependent proteolytic cleavage and regulates the expression of immediate-early genes

Laura Gerosa, Sara Mazzoleni, Francesco Rusconi, Alessandra Longaretti, Elly Lewerissa, Silvia Pelucchi, Luca Murru, Serena Gea Giannelli, Vania Broccoli, Elena Marcello, Nael Nadif Kasri, Elena Battaglioli, Maria Passafaro, and Silvia Bassani

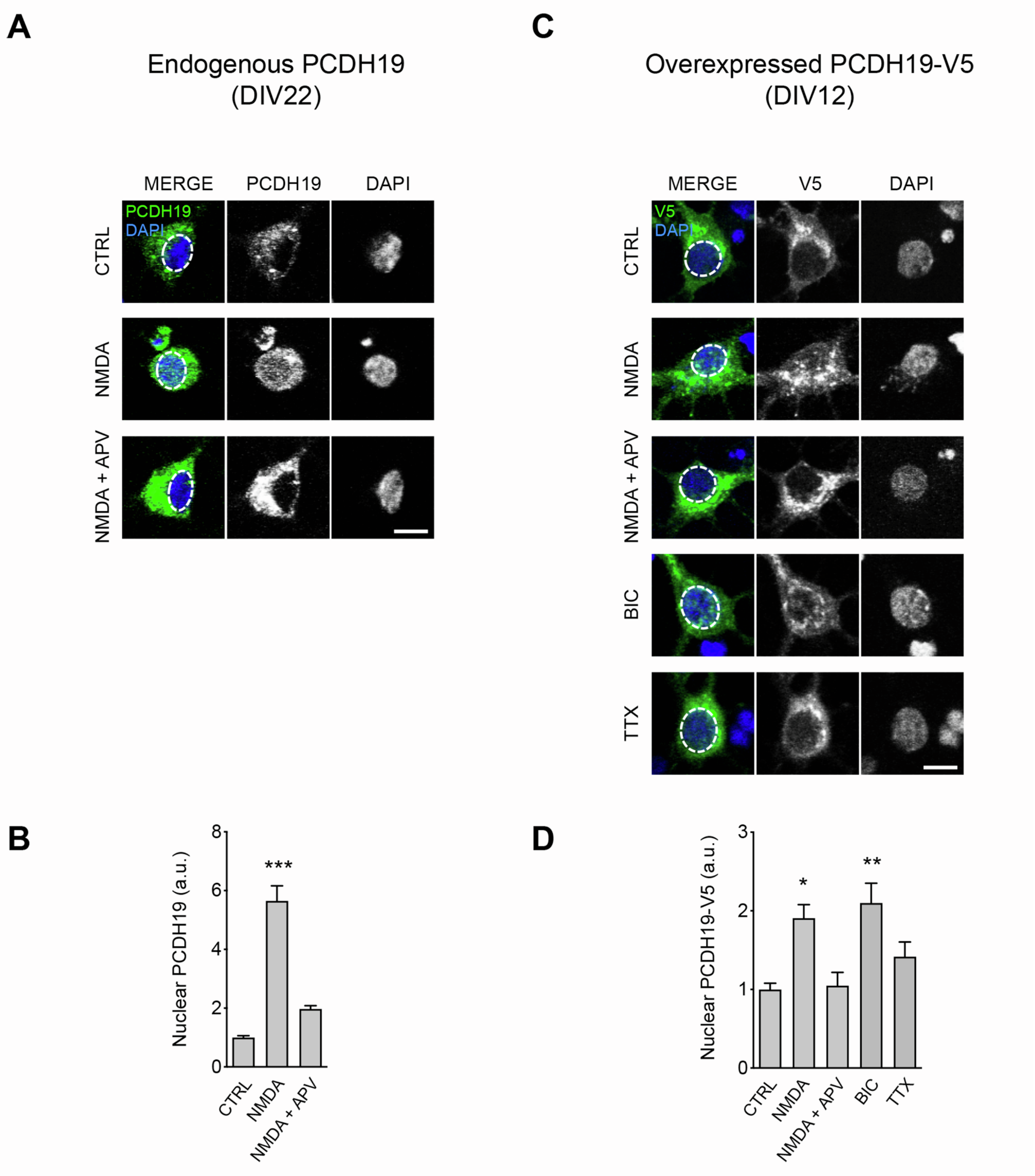

A
Endogenous PCDH19
(DIV22)
MERGE
PCDH19
DAPI
CTRL
NMDA
NMDA + APV
C
Overexpressed PCDH19-V5
(DIV12)
MERGE
V5
DAPI
CTRL
NMDA
NMDA + APV
BIC
TTX
B
Nuclear PCDH19 (a.u.)
***
0
2
4
6
8
CTRL
NMDA
NMDA + APV
D
Nuclear PCDH19-V5 (a.u.)
*
**
0
1
2
3
CTRL
NMDA
NMDA + APV
BIC
TTX

Figure S1. PCDH19 intracellular distribution changes following NMDAR activation in mature neurons expressing endogenous PCDH19 and developing neurons overexpressing PCDH19, Related to Figure 1

(A) Representative images of hippocampal neurons somata and nuclei at DIV 22 immunolabeled for PCDH19 and stained with DAPI, showing PCDH19 distribution after treatments with NMDA, NMDA + APV or vehicle (CTRL). Scale bar, 10 μm.

(B) Quantification of PCDH19 nuclear expression (n, CTRL 9, NMDA 11, NMDA + APV 9; CTRL vs. NMDA ***$p < 0.001$, one-way ANOVA and Dunnett's *post hoc* test). Data are presented as mean ± SEM.

(C) Representative images of hippocampal neurons somata and nuclei from neurons at DIV 12 previously transfected (DIV 4) with PCDH19-V5 and immunolabeled for V5 and stained with DAPI, showing PCDH19 distribution after treatments, as indicated. Scale bar, 10 μm.

(D) Quantification of PCDH19 nuclear expression (n, CTRL 10, NMDA 21, NMDA + APV 6, BIC 17, TTX 13; CTRL vs. NMDA *$p < 0.05$, CTRL vs. BIC **$p < 0.01$, one-way ANOVA and Dunnett's *post hoc* test). Data are presented as mean ± SEM.

Values are shown in Table S8.

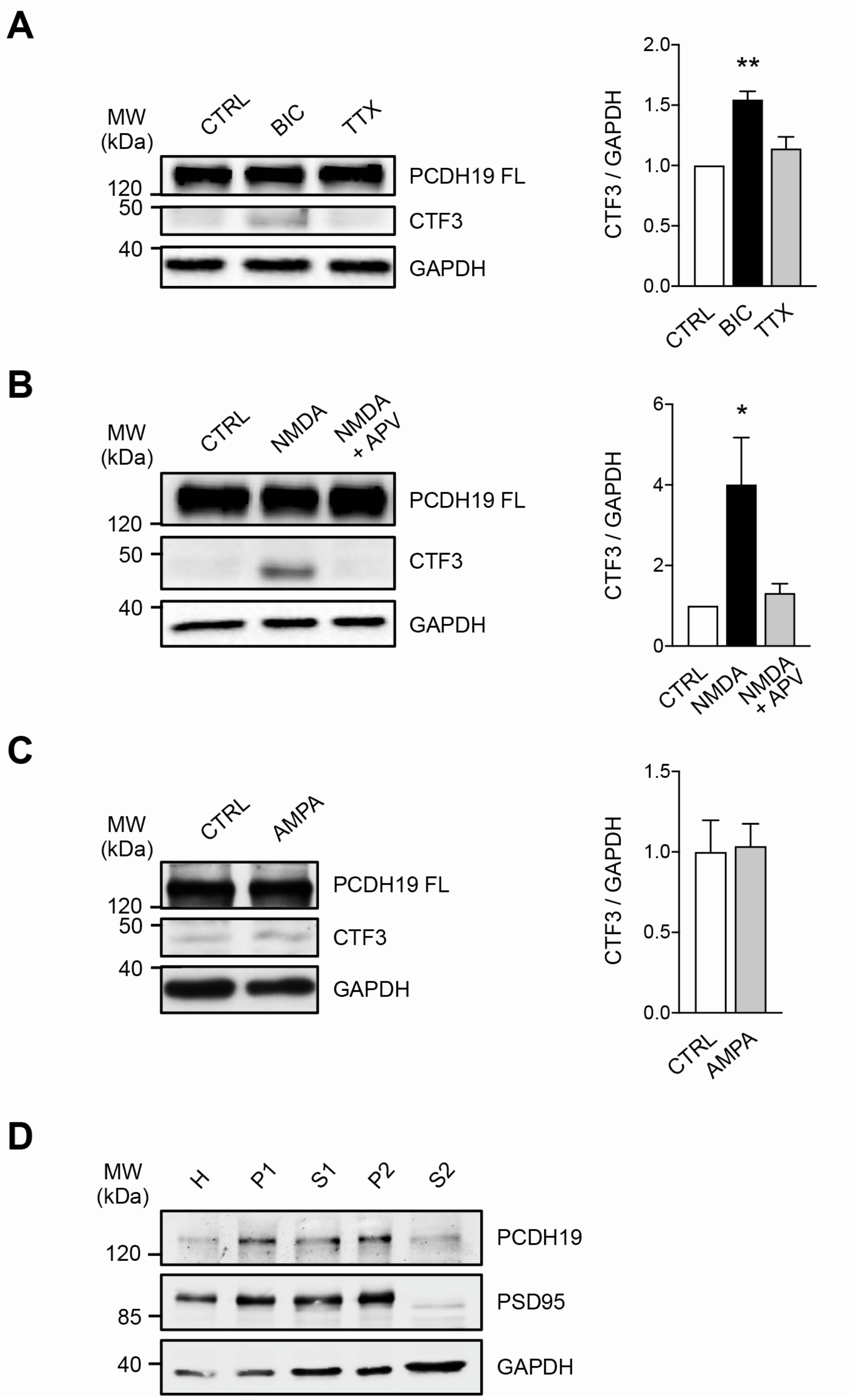
A
MW (kDa)
CTRL
BIC
TTX
PCDH19 FL
120
50
CTF3
40
GAPDH
CTF3 / GAPDH
2.0
1.5
1.0
0.5
0.0
**
B
CTRL
NMDA
NMDA + APV
6
4
2
0
*
C
AMPA
D
H
P1
S1
P2
S2
PCDH19
PSD95
85

Figure S2. PCDH19 proteolytic cleavage in hippocampal neurons and PCDH19 expression in crude synaptosomes, Related to Figure 2

(A) Representative western blots of primary hippocampal neurons at DIV 12 treated with vehicle (CTRL), BIC or TTX and quantification of PCDH19 CTF3 ($n = 3$; $^{**}p < 0.01$, one-way ANOVA and Dunnett's *post hoc* test). GAPDH is used as loading control. Data are presented as mean ± SEM.

(B) Western blots of primary hippocampal neurons at DIV 12 treated with vehicle (CTRL), NMDA or NMDA + APV and quantification of PCDH19 CTF3 ($n = 5$; $^{*}p < 0.05$, one-way ANOVA and Dunnett's *post hoc* test). Data are presented as mean ± SEM.

(C) Western blots of primary hippocampal neurons at DIV 12 treated with vehicle (CTRL) or AMPA and quantification of PCDH19 CTF3 ($n = 3$; $p > 0.05$, n.s., Student t test). Data are presented as mean ± SEM.

(D) Representative western blot of crude synaptosomes from rat brain (hippocampus and cortex). PSD95 is used as postsynaptic protein and GAPDH as loading control. H, total homogenate; P, pellet; S, supernatant. P2 fraction contains crude synaptosomes.

Values are shown in Table S8.

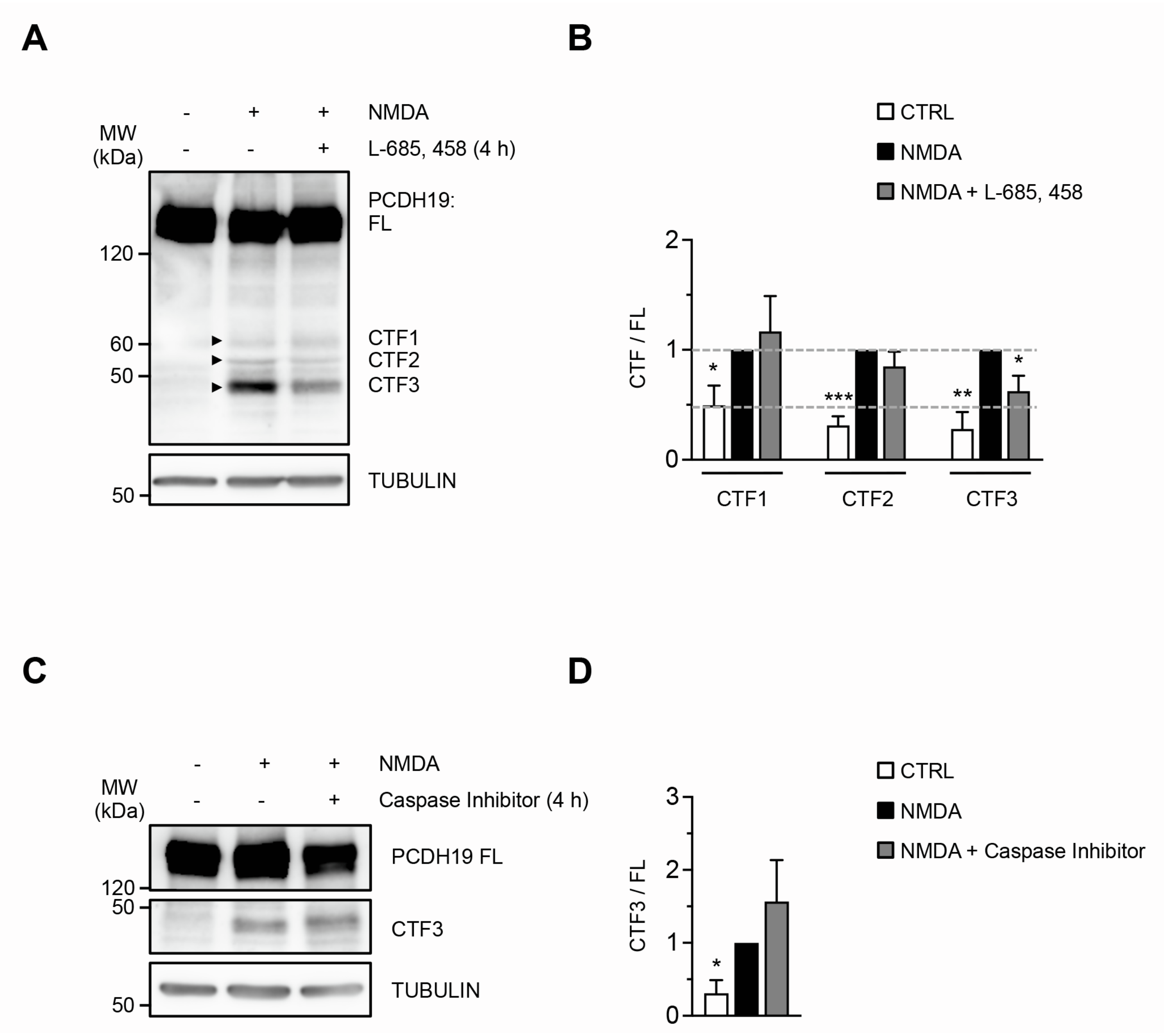

Figure S3. PCDH19 CTF3 level is reduced by gamma secretase inhibitor L-685, 458 but not by caspase inhibitor, Related to Figure 3

(A) Western blots of primary hippocampal neurons at DIV 12 treated with NMDA (6 min) NMDA + L-685, 458 (4 h) or vehicle (CTRL).

(B) Quantification of PCDH19 CTFs in western blots as in A (CTF1 / FL: n = 4; *p < 0.05, Student t test, CTRL compared with NMDA; CTF2 / FL: n = 4; ***p < 0.001, Student t test, CTRL compared with NMDA; CTF3 / FL: n = 4; **p < 0.01, Student t test, CTRL compared with NMDA; *p < 0.05, Student t test, NMDA + L-685, 458 compared with NMDA). Data are presented as mean ± SEM.

(C) Western blots of primary hippocampal neurons at DIV 12 treated with NMDA (6 min) NMDA + caspase inhibitor (4 h) or vehicle (CTRL).

(D) Quantification of PCDH19 CTF3 in western blots as in A (CTF3 / FL: n = 3; *p < 0.05, Student t test). Data are presented as mean ± SEM.

Values are shown in Table S8.

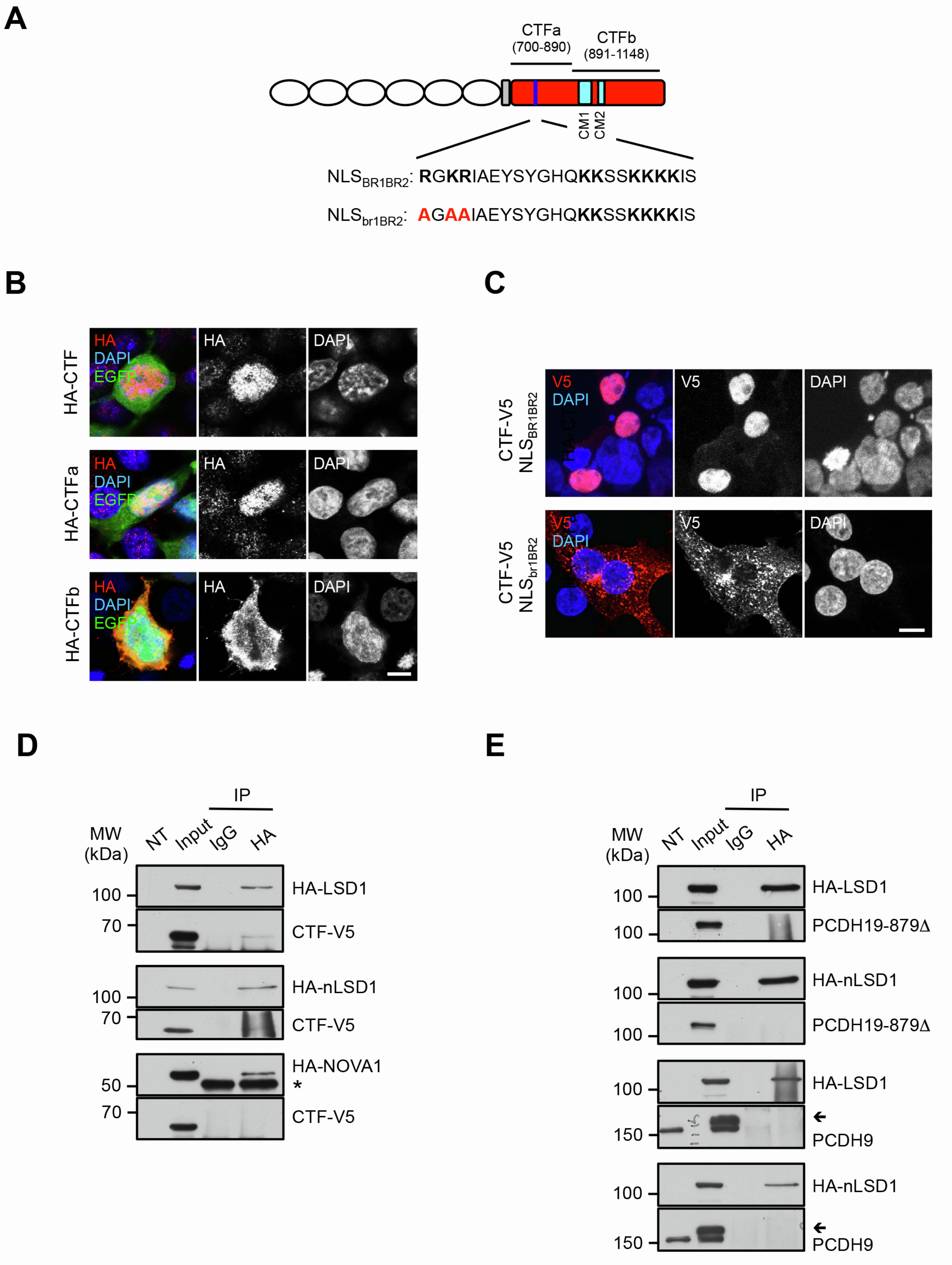

A
CTFa
(700-890)
CTFb
(891-1148)
CM1
CM2
NLSBR1BR2: RGKRIAEYSYGHQKKSSKKKKIS
NLSbr1BR2: AGAAIAEYSYGHQKKSSKKKKIS
B
HA-CTF
HA-CTFa
HA-CTFb
HA
DAPI
EGFP
C
CTF-V5 NLSBR1BR2
CTF-V5 NLSbr1BR2
V5
DAPI
D
IP
MW (kDa)
NT
Input
IgG
HA
HA-LSD1
CTF-V5
HA-nLSD1
HA-NOVA1
*
100
70
50
E
PCDH19-879Δ
PCDH9
150

Figure S4. PCDH19 CTF enters the nucleus and associates with LSD1 in HEK cells, Related to Figures 4 and 5

(A) Scheme of PCDH19 structure, showing PCDH19 intracellular C-terminus (CTF, red) with the nuclear localization signal (dark blue) and the conserved regions CM1 and CM2 (light blue). The amino acids composing the two basic regions of the bipartite NLS (NLS_{BR1BR2}, BR = basic region) are in bold, with the mutated amino acids in red.

(B) HEK cells transfected with HA-CTF, HA-CTFa and HA-CTFb together with pEGFP and stained with HA and DAPI. Scale bar, 10 µm.

(C) HEK cells transfected with wild type CTF-V5 (CTF-V5 NLS_{BR1BR2}) or CTF-V5 with mutated NLS (CTF-V5 NLS_{br1BR2}) and stained with V5 and DAPI. Scale bar, 10 µm.

(D) CoIP in HEK cells cotransfected with HA-LSD1/HA-nLSD1 and CTF-V5 or HA-NOVA1 and CTF-V5, as indicated. Input: 10 % of IP volume. IP: anti-IgG or anti-HA. Western blots probed for HA or V5. The star indicates IgG chains signal. NT, lysate from untransfected cells.

(E) CoIP in HEK cells cotransfected with HA-LSD1 and PCDH19-879Δ, HA-nLSD1 and PCDH19-879Δ, HA-LSD1 and PCDH9, or HA-nLSD1 and PCDH9, as indicated. Input: 10 % of IP volume. IP: anti-IgG or anti-HA. Western blots probed for HA, PCDH19 or PCDH9. The arrow indicates PCDH9 specific band, above of an aspecific signal. NT, lysate from untransfected cells.

Values are shown in Table S8.

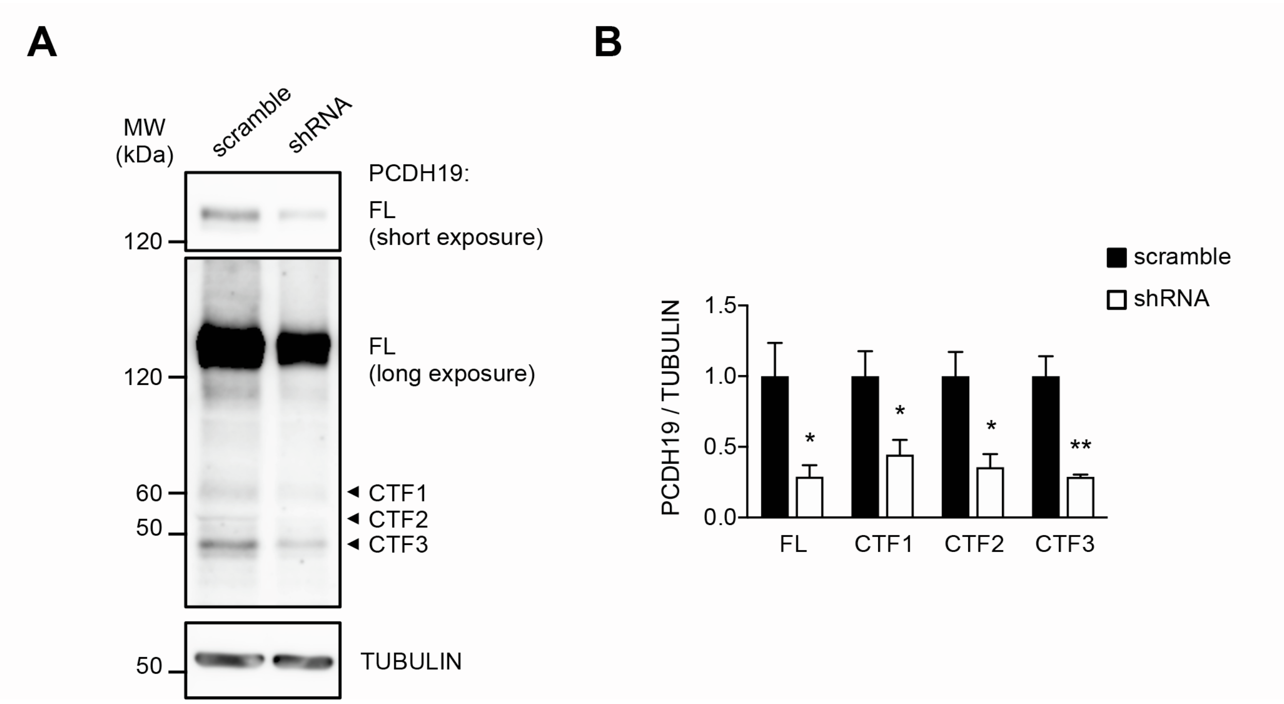

Figure S5. PCDH19 shRNA reduces PCDH19 FL and CTFs levels, Related to Figure 6

(A) Representative western blot of neurons transduced with PCDH19 shRNA (shRNA) or control shRNA (scramble). Neurons were treated with NMDA (50 μM, 6 min).

(B) Quantification of PCDH19 FL and CTFs expression in western blots as in A (PCDH19 / TUBULIN: n = 5; *p < 0.05, **p < 0.01, Student t test). Data are presented as mean ± SEM. Values are shown in Table S8.

Table S1. Mean values, SEM, number of samples and p values of data displayed in Figure 1, Related to Figure 1

**Fig. 1C**<br>**Dendritic PCDH19**	Mean	SEM	N	p value<br>(One-way ANOVA & Dunnett's *post hoc*)	
CTRL	1.000	0.033	17	CTRL vs.:	
BIC	0.764	0.045	23	BIC	$p = 0.004$ (**)
NMDA	0.805	0.036	26	NMDA	$p = 0.019$ (*)
NMDA + APV	1.017	0.060	26	NMDA + APV	$p > 0.05$ (n.s.)
**Fig. 1D**<br>**Nuclear PCDH19**	Mean	SEM	N	p value<br>(One-way ANOVA & Dunnett's *post hoc*)	
CTRL	1.000	0.062	152	CTRL vs.:	
BIC	1.808	0.127	20	BIC	$p < 0.001$ (***)
TTX	0.998	0.113	19	TTX	$p > 0.05$ (n.s.)
NMDA	1.740	0.123	47	NMDA	$p < 0.001$ (***)
NMDA + APV	1.151	0.114	31	NMDA + APV	$p > 0.05$ (n.s.)
SYN. NMDAR	1.529	0.150	61	SYN. NMDAR	$p < 0.001$ (***)
EXTRASYN.<br>NMDAR	1.160	0.118	56	EXTRASYN.<br>NMDAR	$p > 0.05$ (n.s.)

Table S2. Mean values, SEM, number of samples and p values of data displayed in Figure 2, Related to Figure 2

**Fig. 2A PCDH19 FL / GAPDH**	Mean	SEM	N	p value (One-way ANOVA & Tukey's *post hoc*)
CTRL	1.000	0.000	8	CTRL vs. NMDA $p < 0.01$ (**)
NMDA	0.573	0.085	8	CTRL vs. NMDA + APV $p > 0.05$ (n.s.)
NMDA + APV	0.909	0.104	7	NMDA vs. NMDA + APV $p < 0.05$ (*)
**PCDH19 FL / TfR**	Mean	SEM	N	p value (One-way ANOVA & Tukey's *post hoc*)
CTRL	1.000	0.000	4	CTRL vs. NMDA $p < 0.01$ (**)
NMDA	0.536	0.113	4	CTRL vs. NMDA + APV $p > 0.05$ (n.s.)
NMDA + APV	0.914	0.061	4	NMDA vs. NMDA + APV $p < 0.05$ (*)
**Fig. 2B PCDH19 (CTF / FL)**	Mean	SEM	N	p value (Student t test)
CTF1 (CTRL)	1.000	0.733	4	CTF1 (CTRL vs. NMDA) $p > 0.05$ (n.s.)
CTF1 (NMDA)	2.507	1.460	4	
CTF2 (CTRL)	0.570	0.479	4	CTF2 (CTRL vs. NMDA) $p > 0.05$ (n.s.)
CTF2 (NMDA)	3.329	2.000	4	
CTF3 (CTRL)	1.056	0.532	4	CTF3 (CTRL vs. NMDA) $p = 0.017$ (*)
CTF3 (NMDA)	8.789	2.312	4	
CTF1 (CTRL) +MG	0.342	0.088	3	CTF1 (CTRL vs. NMDA) $p > 0.05$ (n.s.)
CTF1 (NMDA) +MG	3.793	2.288	3	
CTF2 (CTRL) +MG	0.386	0.288	4	CTF2 (CTRL vs. NMDA) $p = 0.006$ (**)
CTF2 (NMDA) +MG	4.634	0.983	4	
CTF3 (CTRL) +MG	1.254	0.815	4	CTF3 (CTRL vs. NMDA) $p = 0.028$ (*)
CTF3 (NMDA) +MG	14.148	4.906	3	
**Fig. 2C PCDH19 FL / GAPDH**	Mean	SEM	N	p value (One-way ANOVA & Dunnett's *post hoc*)
CTRL (C)	1.000	0.000	4	CTRL vs.
3 min	1.031	0.102	4	3 min $p > 0.05$ (n.s.)
6 min	0.868	0.124	4	6 min $p > 0.05$ (n.s.)
10 min	0.684	0.091	4	10 min $p < 0.05$ (*)
20 min	0.693	0.048	4	20 min $p > 0.05$ (n.s.)
30 min	0.522	0.049	4	30 min $p < 0.01$ (**)
**CTF3 / GAPDH**	Mean	SEM	N	p value (One-way ANOVA & Dunnett's *post hoc*)
CTRL (C)	1.000	0.000	4	CTRL vs.

3 min	11.405	2.325	4	3 min	p > 0.05 (n.s.)
6 min	19.110	3.343	4	6 min	p < 0.05 (*)
10 min	23.769	3.565	4	10 min	p < 0.01 (**)
20 min	39.834	5.825	4	20 min	p < 0.001 (***)
30 min	23.966	6.653	4	30 min	p < 0.01 (**)
**Fig. 2D**<br>**CTF2 / FL**<br>**(NMDA / CTRL)**	Mean	SEM	N	p value<br>(Student t test)	
H	1.654	0.481	3		
TIF	3.265	0.153	3	H vs. TIF	p = 0.032 (*)

Table S3. Mean values, SEM, number of samples and p values of data displayed in Figure 3, Related to Figure 3

**Fig. 3B PCDH19 (CTF1 / FL)**	Mean	SEM	N	p value (Student t test)	
CTRL	0.169	0.040	4	CTRL vs. NMDA	$p < 0.001$ (***)
NMDA	1.000	0.000	4-5		
NMDA + GM6001	0.186	0.058	5	NMDA + GM6001 vs. NMDA	$p < 0.001$ (***)
NMDA + GI254023X	0.152	0.059	4	NMDA + GI254023X vs. NMDA	$p < 0.001$ (***)
NMDA + DAPT	1.066	0.064	5	NMDA + DAPT vs. NMDA	$p > 0.05$ (n.s.)
**PCDH19 (CTF2 / FL)**	Mean	SEM	N	p value (Student t test)	
CTRL	0.095	0.053	5	CTRL vs. NMDA	$p < 0.001$ (***)
NMDA	1.000	0.000	5-6		
NMDA + GM6001	0.110	0.027	6	NMDA + GM6001 vs. NMDA	$p < 0.001$ (***)
NMDA + GI254023X	0.078	0.031	5	NMDA + GI254023X vs. NMDA	$p < 0.001$ (***)
NMDA + DAPT	1.002	0.087	6	NMDA + DAPT vs. NMDA	$p > 0.05$ (n.s.)
**PCDH19 (CTF3 / FL)**	Mean	SEM	N	p value (Student t test)	
CTRL	0.177	0.123	4	CTRL vs. NMDA	$p < 0.001$ (***)
NMDA	1.000	0.000	3-8		
NMDA + GM6001	0.689	0.081	6	NMDA + GM6001 vs. NMDA	$p = 0.003$ (**)
NMDA + GI254023X	0.560	0.153	3	NMDA + GI254023X vs. NMDA	$p = 0.045$ (*)
NMDA + DAPT	0.809	0.045	8	NMDA + DAPT vs. NMDA	$p < 0.001$ (***)
**Fig. 3D PCDH19 (CTF1 / FL)**	Mean	SEM	N	p value (Student t test)	
CTRL	0.6534	0.116	8	CTRL vs. NMDA	$p = 0.010$ (*)
NMDA	1.000	0.000	4-8		
NMDA + GI254023X 4h	0.340	0.065	5	NMDA + GI254023X vs. NMDA 4h	$p < 0.001$ (***)
NMDA + DAPT 4h	1.625	0.359	6	NMDA + DAPT vs. NMDA 4h	$p > 0.05$ (n.s.)
NMDA + DAPT + GI254023X 4h	0.880	0.188	5	NMDA + DAPT + GI254023X vs. NMDA 4h	$p > 0.05$ (n.s.)
NMDA + GI254023X 24h	0.361	0.052	4	NMDA + GI254023X vs. NMDA 24h	$p < 0.001$ (***)
NMDA + DAPT 24h	3.693	0.582	7	NMDA + DAPT vs. NMDA 24h	$p < 0.001$ (***)
NMDA + DAPT + GI254023X 24h	0.934	0.281	6	NMDA + DAPT + GI254023X vs. NMDA 24h	$p > 0.05$ (n.s.)
**PCDH19**	Mean	SEM	N	p value	

(CTF2 / FL)				(Student t test)	
CTRL	0.381	0.103	8	CTRL vs. NMDA	p < 0.001 (***)
NMDA	1.000	0.000	5-9		
NMDA + GI254023X 4h	0.112	0.057	5	NMDA + GI254023X vs. NMDA 4h	p < 0.001 (***)
NMDA + DAPT 4h	1.861	0.480	5	NMDA + DAPT vs. NMDA 4h	p > 0.05 (n.s.)
NMDA + DAPT + GI254023X 4h	0.414	0.204	5	NMDA + DAPT + GI254023X vs. NMDA 4h	p = 0.020 (*)
NMDA + GI254023X 24h	0.384	0.173	6	NMDA + GI254023X vs. NMDA 24h	p = 0.001 (**)
NMDA + DAPT 24h	3.618	0.671	9	NMDA + DAPT vs. NMDA 24h	p = 0.021 (*)
NMDA + DAPT + GI254023X 24h	1.059	0.361	8	NMDA + DAPT + GI254023X vs. NMDA 24h	p > 0.05 (n.s.)
**PCDH19 (CTF3 / FL)**	Mean	SEM	N	p value (Student t test)	
CTRL	0.302	0.081	9	CTRL vs. NMDA	p < 0.001 (***)
NMDA	1.000	0.000	4-9		
NMDA + GI254023X 4h	0.674	0.086	4	NMDA + GI254023X 4h vs. NMDA	p = 0.009 (**)
NMDA + DAPT 4h	0.677	0.079	5	NMDA + DAPT 4h vs. NMDA	p = 0.003 (**)
NMDA + DAPT + GI254023X 4h	0.542	0.120	4	NMDA + DAPT + GI254023X 4h vs. NMDA	p = 0.009 (**)
NMDA + GI254023X 24h	0.738	0.061	5	NMDA + GI254023X 24h vs. NMDA	p = 0.002 (**)
NMDA + DAPT 24h	1.471	0.247	9	NMDA + DAPT 24h vs. NMDA	p > 0.05 (n.s.)
NMDA + DAPT + GI254023X 24h	1.282	0.250	8	NMDA + DAPT + GI254023X 24h vs. NMDA	p > 0.05 (n.s.)
**Fig. 3E PS1 FL / GAPDH**	Mean	SEM	N	p value (One-way ANOVA & Tukey's *post hoc*)	
NMDA	1.000	0.000	3	NMDA + DAPT 4h vs. NMDA	p > 0.05 (n.s.)
NMDA + DAPT 4h	0.994	0.055	3	NMDA + DAPT 24h vs. NMDA	p > 0.05 (n.s.)
NMDA + DAPT 24h	0.911	0.176	3	NMDA + DAPT 24h vs. NMDA + DAPT 4h	p > 0.05 (n.s.)
**PS1 CTF / GAPDH**	Mean	SEM	N	p value (One-way ANOVA & Tukey's *post hoc*)	
NMDA	1.000	0.000	3	NMDA + DAPT 4h vs. NMDA	p > 0.05 (n.s.)
NMDA + DAPT 4h	1.134	0.059	3	NMDA + DAPT 24h vs. NMDA	p < 0.001 (***)
NMDA + DAPT 24h	1.814	0.109	3	NMDA + DAPT 24h vs. NMDA + DAPT 4h	p = 0.001 (**)

Table S4. Mean values, SEM, number of samples and p values of data displayed in Figure 4, Related to Figure 4

**Fig. 4E**<br>CTF3 (P2 / H)	Mean	SEM	N	p value<br>(One-way ANOVA & Tukey's *post hoc*)
CTRL	1.000	0.000	3	CTRL vs. NMDA p > 0.05 (n.s.)
NMDA	2.295	0.505	3	CTRL vs. NMDA + APV p > 0.05 (n.s.)
NMDA + APV	0.613	0.275	3	NMDA vs. NMDA + APV p = 0.027 (*)
**Fig. 4F**<br>CTF3 / GAPDH	Mean	SEM	N	p value<br>(Student t test)
H (CTRL)	1.000	0.061	5	H:<br>CTRL vs. PILO p = 0.043 (*)
H (PILO)	2.092	0.574	3	
C (CTRL)	0.304	0.081	5	C:<br>CTRL vs. PILO p = 0.003 (**)
C (PILO)	1.390	0.286	3	
CTF3 / PCDH19 FL	Mean	SEM	N	p value<br>(Student t test)
H (CTRL)	1.000	0.259	5	H:<br>CTRL vs. PILO p > 0.05 (n.s.)
H (PILO)	3.059	1.397	3	
N-M (CTRL)	0.639	0.027	4	N-M:<br>CTRL vs. PILO p = 0.033 (*)
N-M (PILO)	2.698	0.847	3	

Table S5. Mean values, SEM, number of samples and p values of data displayed in Figure 5, Related to Figure 5

**Fig. 5C**<br>**LSD1 ChIP**<br>**(% of the input)**	Mean	SEM	N	p value<br>(Student t test)
Nr4a1 mock IP	0.053	0.015	4	Nr4a1:
Nr4a1 LSD1 IP	0.137	0.015	4	IP mock vs. LSD1 p = 0.008 (**)
c-Fos mock IP	0.051	0.014	4	c-Fos:
c-Fos LSD1 IP	0.334	0.051	4	IP mock vs. LSD1 p = 0.001 (**)
Npas4 mock IP	0.018	0.008	4	Npas4:
Npas4 LSD1 IP	0.058	0.008	4	IP mock vs. LSD1 p = 0.017 (*)
**CTF ChIP**<br>**(% of the input)**	Mean	SEM	N	p value<br>(Student t test)
Nr4a1 mock IP	0.042	0.013	3	Nr4a1:
Nr4a1 CTF IP	0.204	0.025	3	IP mock vs. CTF p = 0.004 (**)
c-Fos mock IP	0.045	0.018	3	c-Fos:
c-Fos CTF IP	0.253	0.049	3	IP mock vs. CTF p = 0.017 (*)
Npas4 mock IP	0.010	0.005	3	Npas4:
Npas4 CTF IP	0.088	0.017	3	IP mock vs. CTF p = 0.012 (*)

Table S6. Mean values, SEM, number of samples and p values of data displayed in Figure 6, Related to Figure 6

**Fig. 6A**<br>RT-PCR<br>(rel. mRNA level)	Mean	SEM	N	p value<br>(Student t test)
Nr4a1 (CTRL)	0.685	0.180	3	Nr4a1:
Nr4a1 (CTF-V5)	0.120	0.025	3	CTRL vs. CTF-V5 $p = 0.036$ (*)
c-Fos (CTRL)	0.791	0.181	3	c-Fos:
c-Fos (CTF-V5)	0.143	0.022	3	CTRL vs. CTF-V5 $p = 0.023$ (*)
Egr1 (CTRL)	1.008	0.073	4	Egr1:
Egr1 (CTF-V5)	0.991	0.120	4	CTRL vs. CTF-V5 $p > 0.05$ (n.s.)
Cyr61 (CTRL)	2.253	0.979	3	Cyr61:
Cyr61 (CTF-V5)	1.284	0.715	3	CTRL vs. CTF-V5 $p > 0.05$ (n.s.)
Npas4 (CTRL)	1.002	0.314	3	Npas4:
Npas4 (CTF-V5)	0.080	0.028	3	CTRL vs. CTF-V5 $p = 0.043$ (*)
**Fig. 6B**<br>RT-PCR<br>(rel. mRNA level)	Mean	SEM	N	p value<br>(Student t test)
Nr4a1 (scramble)	0.818	0.094	8	Nr4a1:
Nr4a1 (shRNA)	2.394	0.645	7	scramble vs. shRNA $p = 0.022$ (*)
c-Fos (scramble)	0.659	0.122	8	c-Fos:
c-Fos (shRNA)	0.680	0.163	7	scramble vs. shRNA $p > 0.05$ (n.s.)
Egr1 (scramble)	1.010	0.064	6	Egr1:
Egr1 (shRNA)	1.582	0.153	6	scramble vs. shRNA $p = 0.006$ (**)
Cyr61 (scramble)	0.171	0.046	7	Cyr61:
Cyr61 (shRNA)	0.182	0.059	6	scramble vs. shRNA $p > 0.05$ (n.s.)
Npas4 (scramble)	1.170	0.202	8	Npas4:
Npas4 (shRNA)	2.898	0.857	6	scramble vs. shRNA $p = 0.044$ (*)
**Fig. 6C**<br>NR4A1 intensity	Mean	SEM	N	p value<br>(One way ANOVA and Tukey's *post hoc*)
scramble (vehicle)	1.000	0.080	58	scramble (veh.) vs. :<br>scramble (NMDA) $p < 0.001$ (***)
scramble (NMDA)	1.644	0.173	32	CTF-V5 NLS_{BR1BR2}<br>(NMDA) $p > 0.05$ (n.s.)
CTF-V5 NLS_{BR1BR2}<br>(NMDA)	1.080	0.172	20	CTF-V5 NLS_{br1BR2}<br>(NMDA) $p = 0.024$ (*)
CTF-V5 NLS_{br1BR2}<br>(NMDA)	1.620	0.124	16	shRNA (veh.) $p = 0.006$ (**)
shRNA (vehicle)	1.535	0.116	35	Other comparisons $p > 0.05$ (n.s.)
scramble (DAPT)	1.000	0.055	15	scramble (DAPT) vs. :
shRNA + PCDH19	1.152	0.084	17	shRNA + PCDH19 $p > 0.05$ (n.s.)
shRNA + PCDH19<br>(DAPT)	1.397	0.066	14	shRNA + PCDH19 (DAPT) $p = 0.001$ (**)<br>shRNA + PCDH19 vs. :<br>shRNA + PCDH19 (DAPT) $p > 0.05$ (n.s.)

**Fig. 6D**<br>**% nLSD1**	Mean	SEM	N	p value<br>(Student t test)
CTRL	62.240	1.372	6	
CTF	64.669	2.174	6	CTRL vs. CTF $p > 0.05$ (n.s.)
scramble	55.931	0.435	6	
shRNA	70.165	0.578	6	scramble vs. shRNA $p < 0.001$ (***)
**Fig. 6E**<br>**NOVA1 expression**	Mean	SEM	N	p value<br>(One way ANOVA and Dunnett's *post hoc*)
scramble	1.018	0.029	4	scramble vs.:
shRNA	1.934	0.186	4	shRNA $p = 0.004$ (**)
rescue	0.884	0.214	3	rescue $p > 0.05$ (n.s.)

Table S7. Mean values, SEM, number of samples and p values of data displayed in Figure 7, Related to Figure 7

**Fig. 7B**<br>PCDH19 (CTF/FL)	Mean	SEM	N	p value<br>(One-way ANOVA & Tukey's post hoc)
CTRL	1.000	0.143	3	CTRL vs. NMDA p = 0.001 (**)
NMDA	6.434	0.941	3	CTRL vs. NMDA + APV p > 0.05 (n.s.)
NMDA + APV	1.530	0.381	3	NMDA vs. NMDA + APV p = 0.002 (**)
**Fig. 7D**<br>Nuclear PCDH19	Mean	SEM	N	p value<br>(Student t test)
CTRL	1.000	0.057	12	
NMDA	3.196	0.513	12	CTRL vs. NMDA p < 0.001 (***)
**Fig. 7E**<br>RT-PCR<br>(rel. mRNA level)	Mean	SEM	N	p value<br>(Student t test)
PCDH19 (scramble)	1.000	0.000	3	PCDH19:
PCDH19 (shRNA)	0.302	0.050	3	scramble vs. shRNA p < 0.001 (***)
**Fig. 7F**<br>RT-PCR<br>(rel. mRNA level)	Mean	SEM	N	p value<br>(Student t test)
NR4A1 (scramble)	0.8632	0.071	7	NR4A1:
NR4A1 (shRNA)	0.8709	0.149	8	scramble vs. shRNA p > 0.05 (n.s.)
c-FOS (scramble)	0.8834	0.146	7	c-FOS:
c-FOS (shRNA)	1.134	0.166	8	scramble vs. shRNA p > 0.05 (n.s.)
EGR1 (scramble)	0.8473	0.094	5	EGR1:
EGR1 (shRNA)	2.400	0.536	5	scramble vs. shRNA p = 0.03 (*)
CYR61 (scramble)	0.8835	0.079	5	CYR61:
CYR61 (shRNA)	1.219	0.289	6	scramble vs. shRNA p > 0.05 (n.s.)
NPAS4 (scramble)	1.039	0.039	4	NPAS4:
NPAS4 (shRNA)	4.549	0.384	4	scramble vs. shRNA p = 0.001 (**)

Table S8. Mean values, SEM, number of samples and p values of data displayed in Figures S1, S2, S3, and S5, Related to Figures 1, S1, 2, S2, 3, S3, 6 and S5

**Fig. S1B**<br>Nuclear PCDH19	Mean	SEM	N	p value<br>(One-way ANOVA & Dunnett's *post hoc*)
CTRL	1.000	0.060	9	CTRL vs.:
NMDA	5.649	0.512	11	NMDA $p < 0.001$ (***)
NMDA + APV	1.970	0.114	9	NMDA + APV $p > 0.05$ (n.s.)
**Fig. S1D**<br>Nuclear PCDH19-V5	Mean	SEM	N	p value<br>(One-way ANOVA & Dunnett's *post hoc*)
CTRL	1.000	0.079	10	CTRL vs.:
NMDA	1.905	0.173	21	NMDA $p = 0.011$ (*)
NMDA + APV	1.048	0.169	6	NMDA + APV $p > 0.05$ (n.s.)
BIC	2.098	0.252	17	BIC $p = 0.002$ (**)
TTX	1.415	0.186	13	TTX $p > 0.05$ (n.s.)
**Fig. S2A**<br>CTF3 / GAPDH	Mean	SEM	N	p value<br>(One way ANOVA and Dunnett's *post hoc*)
CTRL	1.000	0.000	3	CTRL vs.:
BIC	1.545	0.069	3	BIC $p = 0.002$ (**)
TTX	1.140	0.095	3	TTX $p > 0.05$ (n.s.)
**Fig. S2B**<br>CTF3 / GAPDH	Mean	SEM	N	p value<br>(One way ANOVA and Dunnett's *post hoc*)
CTRL	1.000	0.000	5	CTRL vs.:
NMDA	4.008	1.163	5	NMDA $p = 0.016$ (*)
NMDA + APV	1.314	0.234	5	NMDA + APV $p > 0.05$ (n.s.)
**Fig. S2C**<br>CTF3 / GAPDH	Mean	SEM	N	p value<br>(Student t test)
CTRL	1.000	0.196	3	CTRL vs.
AMPA	1.036	0.138	3	AMPA $p > 0.05$ (n.s.)
**Fig. S3B**<br>**PCDH19 (CTF1 / FL)**	Mean	SEM	N	p value<br>(Student t test)
CTRL	0.494	0.180	4	NMDA vs. :
NMDA	1.000	0.000	4	CTRL $p = 0.031$ (*)
NMDA + L-685, 458	1.167	0.321	4	NMDA + L-685, 458 $p > 0.05$ (n.s.)
**PCDH19 (CTF2 / FL)**	Mean	SEM	N	p value<br>(Student t test)
CTRL	0.312	0.083	4	NMDA vs. :
NMDA	1.000	0.000	4	CTRL $p < 0.001$ (***)
NMDA + L-685, 458	0.848	0.134	4	NMDA + L-685, 458 $p > 0.05$ (n.s.)

**PCDH19 (CTF3 / FL)**	Mean	SEM	N	p value (Student t test)
CTRL	0.280	0.154	4	NMDA vs. :
NMDA	1.000	0.000	4	CTRL p = 0.003 (**)
NMDA + L-685, 458	0.623	0.140	4	NMDA + L-685, 458 p = 0.036 (*)

**Fig. S3D PCDH19 (CTF3 / FL)**	Mean	SEM	N	p value (Student t test)
CTRL	0.308	0.180	3	NMDA vs. :
NMDA	1.000	0.000	3	CTRL p = 0.018 (*)
NMDA + Caspase Inh.	1.566	0.568	3	NMDA + Caspase Inh. p > 0.05 (n.s.)

**Fig. S5B PCDH19 / TUBULIN**	Mean	SEM	N	p value (Student t test)
FL (scramble)	1.000	0.235	5	
FL (shRNA)	0.289	0.080	5	scramble vs. shRNA p = 0.021 (*)
CTF1 (scramble)	1.000	0.176	5	
CTF1 (shRNA)	0.445	0.103	5	scramble vs. shRNA p = 0.026 (*)
CTF2 (scramble)	1.000	0.171	5	
CTF2 (shRNA)	0.355	0.092	5	scramble vs. shRNA p = 0.010 (*)
CTF3 (scramble)	1.000	0.141	5	
CTF3 (shRNA)	0.287	0.017	5	scramble vs. shRNA p = 0.001 (**)

Table S9. Oligonucleotides sequences used for data in Figures 4, S4, 5, 6, and 7, Related to Figures 4, S4, 5, 6, and 7

**Figs. 4, S4<br>Name:**	**Primers for mutagenesis<br>Sequence (5' - 3'):**
Fw	GGAGAAAGTGAGCCTAGCGGGAGCGGCAATTGCTGAGTACTCCTATGG
Rev	CCATAGGAGTACTCAGCAATTGCCGCTCCCGCTAGGCTCACTTCTCC
**Fig. 5<br>Name:**	**Primers for ChIP experiments<br>Sequence (5' - 3'):**
Nr4a1, Fw	GTCACGGAGCGCTTAAGATGT
Nr4a1, Rev	TCCGCTCCGCAGTCCTTCTA
c-Fos, Fw	TCAGAGTTGGCTGCAGCCGGC
c-Fos, Rev	GCGTGTAGGATTTCGGAGATG
Npas4, Fw	AAAGGGTCTTGGGTAGGTGC
Npas4, Rev	CCTCCGCACAGCTCTAGAAAA
**Fig. 6<br>Name:**	**Primers for RT-PCR<br>Sequence (5' - 3'):**
RPSA, Fw	ACCCAGAGGAGATTGAGAAGG
RPSA, Rev	TGGGGAACTGCTGAATGGGC
Nr4a1, Fw	GAAGCTCATCTTCTGCTCAGG
Nr4a1, Rev	CCATGTCGATCAGTGATGAGG
c-Fos, Fw	TCAGAGTTGGCTGCAGCCGGC
c-Fos, Rev	GCGTGTAGGATTTCGGAGATG
Egr1, Fw	TTCAATCCTCAAGGGGAGC
Egr1, Rev	AACCGGGTAGTTTGGCTGGGA
Cyr61, Fw	AACGAGGACTGCAGCAAAACG
Cyr61, Rev	TCTGAGTGAGCTCTGCAGATC
Npas4, Fw	GGTGGTGAGACTTCAAGCCAA
Npas4, Rev	TCCGTGTCACTGATAGGGTAG
**Fig. 7<br>Name:**	**Primers for RT-PCR<br>Sequence (5' - 3'):**
PCDH19, Fw	AACGATGTGCTGAACACCAG
PCDH19, Rev	GCATCCAGCACCTGTCAGAGT
NR4A1, Fw	CACAGCTTGCTTGTCGATGTC
NR4A1, Rev	ATGCCGGTCGGTGATGAG
c-FOS, Fw	GCGTTGTGAAGACCATGACAG
c-FOS, Rev	TCTAGTTGGTCTGTCTCCGCT
EGR1, Fw	ACCTGACCGCAGAGTCTTTTC
EGR1, Rev	GTGGTTTGGCTGGGGTAACT
CYR61, Fw	GAAGCGGCTCCCTGTTTTTG
CYR61, Rev	CGGGTTTCTTTCACAAGGCG
NPAS4, Fw	CTGCATCTACACTCGCAAGG
NPAS4, Rev	GCCACAATGTCTTCAAGCTCT
