# Supplementary material for: The epilepsy-associated protein PCDH19 undergoes NMDA receptor-dependent proteolytic cleavage and regulates the expression of immediate-early genes
Source: Cell Rep. 2022 May 24;39(8):110857. doi: 10.1016/j.celrep.2022.110857 (PMC9152703; doi:10.1016/j.celrep.2022.110857)
Supplement: Document S2. Article plus supplemental information [file mmc2.pdf]

Cell Reports

Article

The epilepsy-associated protein PCDH19 undergoes NMDA receptor-dependent proteolytic cleavage and regulates the expression of immediate-early genes

Graphical abstract

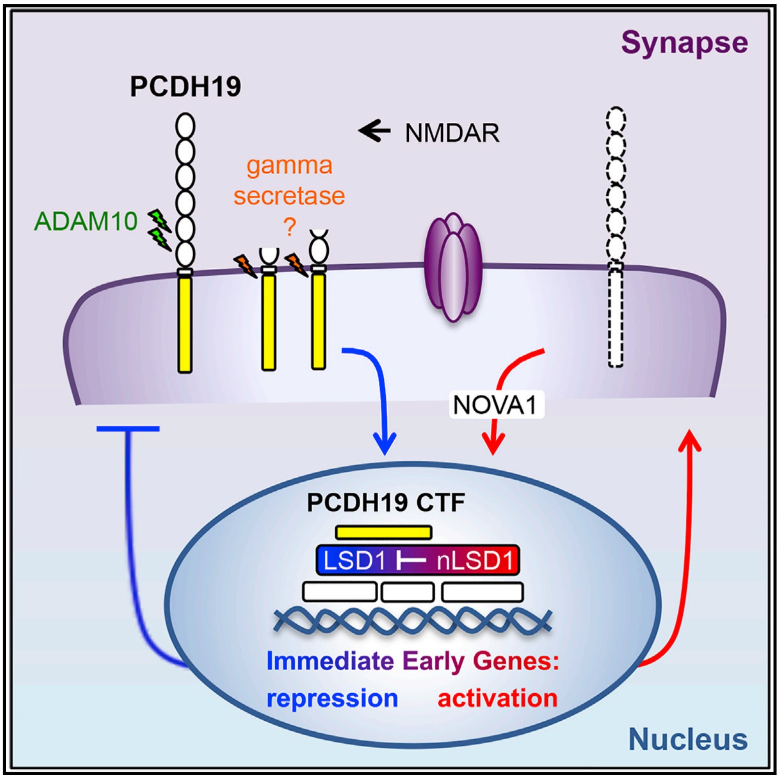

Highlights

- PCDH19 undergoes NMDAR-dependent cleavage by ADAM10 and possibly gamma secretase
- In the nucleus, PCDH19 C-terminal fragment (CTF) associates with the chromatin remodeler LSD1
- PCDH19 CTF favors immediate-early gene (IEG) repression
- PCDH19 downregulation affects LSD1 splicing by NOVA1 and increases IEG expression

Authors

Laura Gerosa, Sara Mazzoleni, Francesco Rusconi, ..., Elena Battaglioli, Maria Passafaro, Silvia Bassani

Correspondence

silvia.bassani@in.cnr.it

In brief

Gerosa et al. report a synapse-to-nucleus signaling pathway through which PCDH19 bridges neuronal activity with gene expression. Their findings are consistent with a model whereby PCDH19 favors the maintenance of neuronal homeostasis via negative-feedback regulation of immediate-early gene (IEG) expression.

Gerosa et al., 2022, Cell Reports *39*, 110857

May 24, 2022 © 2022 The Author(s).

https://doi.org/10.1016/j.celrep.2022.110857

CellPress

Cell Reports

CellPress
OPEN ACCESS

Article

The epilepsy-associated protein PCDH19 undergoes NMDA receptor-dependent proteolytic cleavage and regulates the expression of immediate-early genes

Laura Gerosa,[1] Sara Mazzoleni,[1,2,7] Francesco Rusconi,[2,7] Alessandra Longaretti,[2] Elly Lewerissa,[3] Silvia Pelucchi,[4] Luca Murru,[1,5] Serena Gea Giannelli,[6] Vania Broccoli,[1,6] Elena Marcello,[4] Nael Nadif Kasri,[3] Elena Battaglioli,[1,2] Maria Passafaro,[1,5] and Silvia Bassani[1,5,8,*]

[1]Institute of Neuroscience, CNR, 20854 Vedano al Lambro, Italy
[2]Department of Medical Biotechnology and Translational Medicine, University of Milan, 20129 Milano, Italy
[3]Donders Institute for Brain, Cognition, and Behaviour, Department of Human Genetics, Department of Human Genetics Cognitive Neuroscience, Radboud University Nijmegen Medical Centre, Nijmegen, the Netherlands
[4]Department of Pharmacological and Biomolecular Sciences, University of Milan, 20133 Milano, Italy
[5]NeuroMI Milan Center for Neuroscience, University of Milano-Bicocca, 20126 Milano, Italy
[6]Stem Cell and Neurogenesis Unit, Division of Neuroscience, San Raffaele Scientific Institute, 20132 Milano, Italy
[7]These authors contributed equally
[8]Lead contact
*Correspondence: silvia.bassani@in.cnr.it
https://doi.org/10.1016/j.celrep.2022.110857

SUMMARY

Protocadherin-19 (PCDH19) is a synaptic cell-adhesion molecule encoded by X-linked *PCDH19*, a gene linked with epilepsy. Here, we report a synapse-to-nucleus signaling pathway through which PCDH19 bridges neuronal activity with gene expression. In particular, we describe the NMDA receptor (NMDAR)-dependent proteolytic cleavage of PCDH19, which leads to the generation of a PCDH19 C-terminal fragment (CTF) able to enter the nucleus. We demonstrate that PCDH19 CTF associates with chromatin and with the chromatin remodeler lysine-specific demethylase 1 (LSD1) and regulates expression of immediate-early genes (IEGs). Our results are consistent with a model whereby PCDH19 favors maintenance of neuronal homeostasis via negative feedback regulation of IEG expression and provide a key to interpreting *PCDH19*-related hyperexcitability.

INTRODUCTION

Protocadherin-19 (PCDH19) is a cell-adhesion molecule (CAM) encoded by the *PCDH19* gene (Xq22.1). *PCDH19* mutations cause developmental and epileptic encephalopathy 9 (DEE9; OMIM: 300088), a neurodevelopmental disorder characterized by seizures, cognitive impairment, autism spectrum disorder (ASD), and psychiatric symptoms including schizophrenia (Dibbens et al., 2008; Kolc et al., 2018; Vlaskamp et al., 2019). PCDH19 is highly expressed in the hippocampus, where it has been detected at synapses (Hayashi et al., 2017), and forms homodimers *in trans* (Cooper et al., 2016) and heterodimers *in cis* with NCAD (Emond et al., 2011). PCDH19 adhesive properties are thought to set the basis for neuronal recognition, sorting, and interaction (Cooper et al., 2015; Hayashi et al., 2017; Pederick et al., 2018), but a comprehensive understanding of PCDH19 function is lagging behind.

It is well accepted that cell-adhesion molecules (CAMs), in addition to a structural role, also play functional roles and are directly involved in both Hebbian and homeostatic forms of plasticity (Nagappan-Chettiar et al., 2017). In fact, several CAMs undergo a two-step proteolytic cleavage, which typically depends on glutamate receptor activation. First, matrix metalloproteases or other proteases such metalloproteases containing a disintegrin domain (ADAM) cut the target protein extracellularly, causing ectodomain shedding. Next, the resulting membrane-bound protein stump is cut within the transmembrane region or at the inner membrane surface by presenilin, the catalytic component of the gamma secretase complex, thus releasing the cytoplasmic domain (McCusker and Alfandari, 2009). The cytoplasmic domain can exert a signaling function in the cytoplasm or in the nucleus, where it can modulate gene expression directly or indirectly, for instance via protein-protein interaction with transcriptional regulators (Bouza et al., 2021; Du et al., 2014; Marambaud et al., 2003). Epigenetics, which encompasses all those processes that regulate gene expression by modifying chromatin structure without changing the DNA sequence (Goldberg et al., 2007), is emerging as a mechanism to regulate synaptic plasticity and cognitive processes. In fact, the transcription of genes such as immediate-early genes (IEGs)

© 2022 The Author(s).
This is an open access article under the CC BY license (http://creativecommons.org/licenses/by/4.0/).

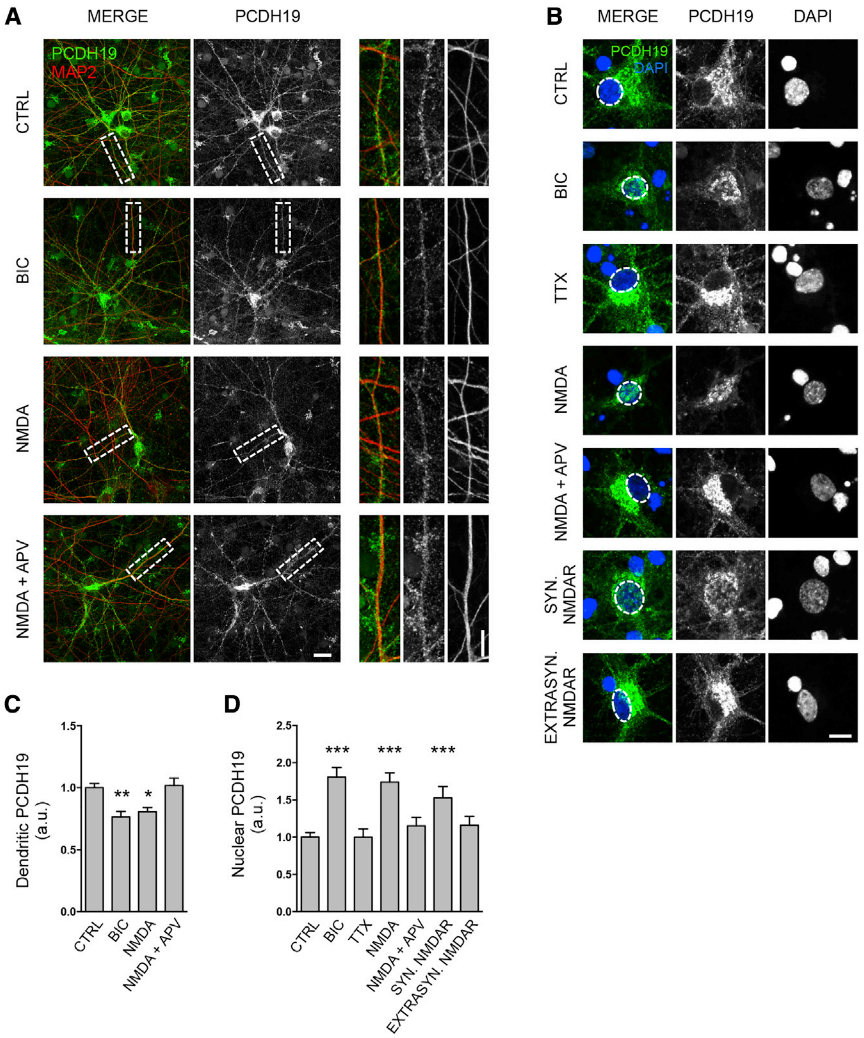

Figure 1. PCDH19 intracellular distribution changes according to neuronal activity

(A) Confocal images of rat hippocampal neurons immunolabeled for PCDH19 and MAP2 at DIV 12 after treatment with BIC, NMDA, NMDA + APV, or vehicle (CTRL). Scale bar, 20 μm. Insets show higher magnification of dendrites highlighted in white. Scale bar, 10 μm.

(B) Representative images of hippocampal neurons somata and nuclei at DIV 12 immunolabeled for PCDH19 and stained with DAPI, showing PCDH19 distribution after drug treatments, as indicated. Scale bar, 10 μm.

(C) Quantification of PCDH19 dendritic expression (n, CTRL 17, BIC 23, NMDA 26, NMDA + APV 26; *$p < 0.05$, **$p < 0.01$, one-way ANOVA and Dunnett's post hoc test). Data are presented as mean ± SEM.

(D) Quantification of PCDH19 nuclear expression (n, CTRL 152, BIC 20, TTX 19, NMDA 47, NMDA + APV 31, synaptic NMDARs activation 61, extrasynaptic NMDARs activation 56; ***$p < 0.001$, one-way ANOVA and Dunnett's post hoc test). Data are presented as mean ± SEM. Values are shown in Table S1.

See also Figure S1 and Table S8.

allows translating patterns of neuronal activity into structural and functional long-term synaptic changes (Campbell and Wood, 2019; Clayton et al., 2020; Kim et al., 2018). The nuclear translocation of synaptic proteins is one of the mechanisms proposed to transmit information from synapses to nucleus (reviewed in Campbell and Wood, 2019). However, the mechanisms by which synaptic proteins can influence epigenetic mechanisms to transfer information are largely unknown.

Here, we describe the NMDA receptor (NMDAR)-dependent proteolytic processing of PCDH19 and unveil a nuclear role of PCDH19, which is conserved from rodents to humans. In particular, we report the crosstalk between PCDH19 and the chromatin remodeler lysine-specific demethylase 1 (LSD1). LSD1 is a transcriptional corepressor of the corepressor for the RE1-silencing transcription factor (CoREST)/HDAC2 complex that provides a bridge between neuronal activity and IEGs (Longaretti et al., 2020; Rusconi et al., 2016). Through the interplay with LSD1, we show that PCDH19 is able to regulate IEG expression in a way that suggests a homeostatic control of neuronal activity.

RESULTS

Neuronal activity affects PCDH19 intracellular distribution

Under basal condition, PCDH19 is mainly distributed in the perinuclear region and along dendrites of hippocampal primary rodent neurons (Figures 1A and 1B). However, we observed that PCDH19 distribution changes according to neuronal activity. Following bath application of bicuculline (BIC), to globally increase the network activity, or NMDA, but not NMDA plus its selective antagonist APV (NMDA + APV), PCDH19 expression decreased along dendrites and increased in the nucleus, as evaluated by immunocytochemistry (ICC) with an antibody against the PCDH19 intracellular C-terminal domain (Figures 1A–1D). Treatment with BIC and 4-aminopyridine (4-AP), which activates the synaptic pool of NMDARs, was enough to trigger the increase of the PCDH19 nuclear signal. By contrast, the selective activation of extrasynaptic NMDARs, achieved by NMDA application after pre-blocking synaptic NMDARs with the open channel blocker MK-801 (Karpova et al., 2013), as well as suppression of network activity by TTX, did not affect PCDH19 distribution (Figures 1B and 1D).

The NMDAR-dependent nuclear increase of endogenously expressed PCDH19 occurred at different developmental stages, i.e., in both developing (days *in vitro* [DIV] 12; Figure 1) and mature neurons (DIV 22; Figures S1A and S1B), and a similar nuclear relocalization was also observed when we overexpressed human PCDH19 (Figures S1C and S1D). Altogether, these data reveal the redistribution of PCDH19 signal

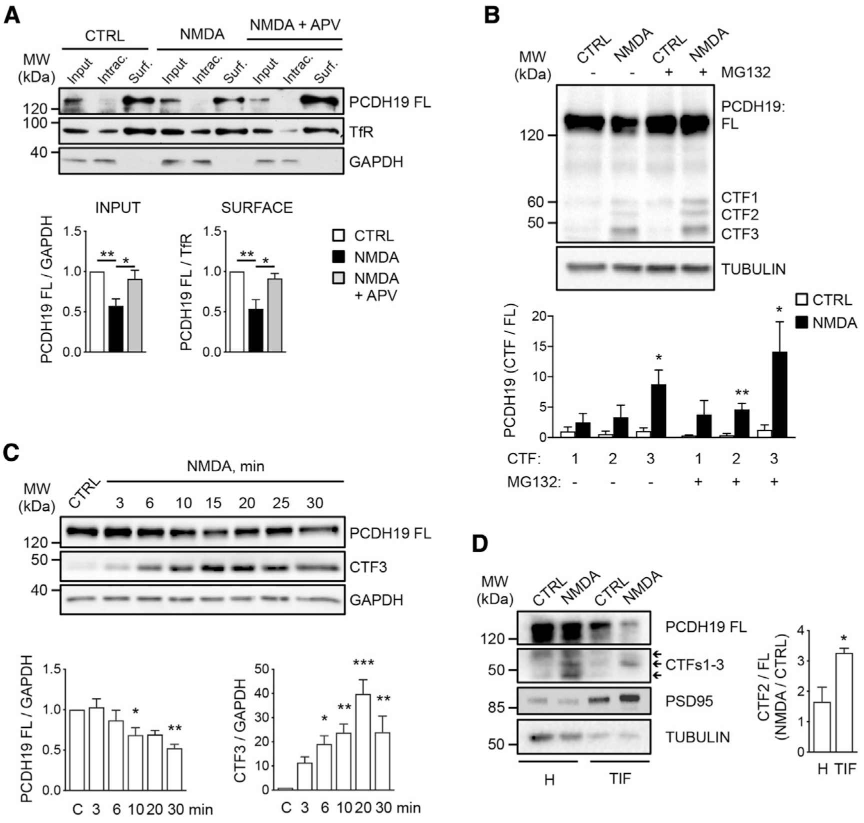

Figure 2. PCDH19 undergoes NMDAR-dependent proteolytic cleavage

(A) Biotinylation assay of neurons at DIV 12 treated with vehicle (CTRL), NMDA, or NMDA + APV, and quantification of PCDH19 in total lysate and on the surface (n input = 7–8, n surface = 4; *p < 0.05, **p < 0.01, one-way ANOVA and Tukey's post hoc test). Data are presented as mean ± SEM. Intrac., non-biotinylated intracellular protein fraction. Surf., biotin-labeled surface protein fraction. TfR, transferrin receptor.

(B) Representative western blot of neurons treated with vehicle (CTRL) or NMDA, in presence of MG132 or not, as indicated (top), and quantification of PCDH19 CTF1-3 normalized on PCDH19 FL (bottom) (n = 3–4; *p < 0.05, **p < 0.01, Student's t test). Data are presented as mean ± SEM.

(C) Time course of NMDA treatment (top) and relative quantification (bottom) (n = 4; *p < 0.05, **p < 0.01, ***p < 0.001, one-way ANOVA and Dunnett's post hoc test). Data are presented as mean ± SEM. CTRL, C = vehicle-treated neurons.

(D) Western blot of total homogenate (H) and TIF fraction from neurons treated with vehicle (CTRL) or NMDA (left), and quantification of CTF2 enrichment in the TIF of NMDA-treated neurons (n = 3; *p < 0.05, Student's t test) (right). Data are presented as mean ± SEM. Values are shown in Table S2.

See also Figure S2 and Table S8.

from dendrites to the nucleus in response to NMDAR activation.

PCDH19 undergoes NMDAR-dependent proteolytic cleavage

To investigate the mechanisms underlying PCDH19 activity-dependent redistribution, we performed biochemical assays in hippocampal neurons by using an antibody against PCDH19 C-terminal domain, as before. Bath application of NMDA (50 μM, 30 min), but not NMDA + APV, caused a significant decrease of full-length PCDH19 (PCDH19 FL) in the total lysate and in the plasma-membrane fraction (Figures 2A and 2C). This was paralleled by a simultaneous increase, in the total lysate, of three low-molecular-weight bands running at about 60, 55, and 45 kDa that we named C-terminal fragment (CTF) 1, CTF2, and CTF3 and whose amount tended to increase following proteasome inhibition with MG132 (Figure 2B). A closer examination of the most abundant fragment, CTF3, revealed that it significantly increased after 6 min of NMDA application and peaked after 20 min (Figure 2C). BIC and the activation of NMDARs caused the increase of CTF3 level; by contrast, tetrodotoxin (TTX), NMDA + APV, and the activation of AMPA receptor (AMPAR) were ineffective (Figures S2A–S2C). Since PCDH19 is expressed at synapses (Hayashi et al., 2017; Figure S2D), we prepared Triton insoluble fractions (TIFs) from control neurons (CTRL) and NMDA-treated neurons (30 min), and we looked at PCDH19 CTFs. Interestingly, we observed that NMDA-dependent enrichment of CTF2 was significantly higher in TIFs with respect of the total homogenate, while CTF3 was barely detectable and not enriched in the TIFs of NMDA-treated neurons (Figure 2D). Based on these results, we hypothesized that PCDH19 might be cleaved at synapses, generating a soluble CTF3 fragment.

To verify whether PCDH19 might undergo a two-step proteolytic cleavage mediated by metalloproteases and gamma secretase, we performed experiments with protease inhibitors. Ideally, any protein fragment whose generation is prevented by a specific protease inhibitor is expected to be the direct product of that protease or a downstream product, whereas any protein fragment that accumulates is expected to be the substrate of that protease.

In a first set of experiments, we treated neurons for 40 min (20 min prior and 20 min during NMDA application, 50 μM) with the broad-spectrum metalloprotease inhibitor GM6001 and the specific gamma secretase inhibitor DAPT, together with the proteasome inhibitor MG132 in order to facilitate CTFs detection. We also tested GI254023X, the specific inhibitor of ADAM10, since ADAM10 is responsible for the ectodomain shedding of other protocadherins (Bonn et al., 2007; Bouillot et al., 2011; Reiss et al., 2006). CTF1 and CTF2 were strongly reduced by both metalloprotease inhibitors, while they were unaffected by DAPT. By contrast, CTF3 was significantly reduced, albeit partially, by both metalloprotease inhibitors and DAPT (Figures 3A and 3B).

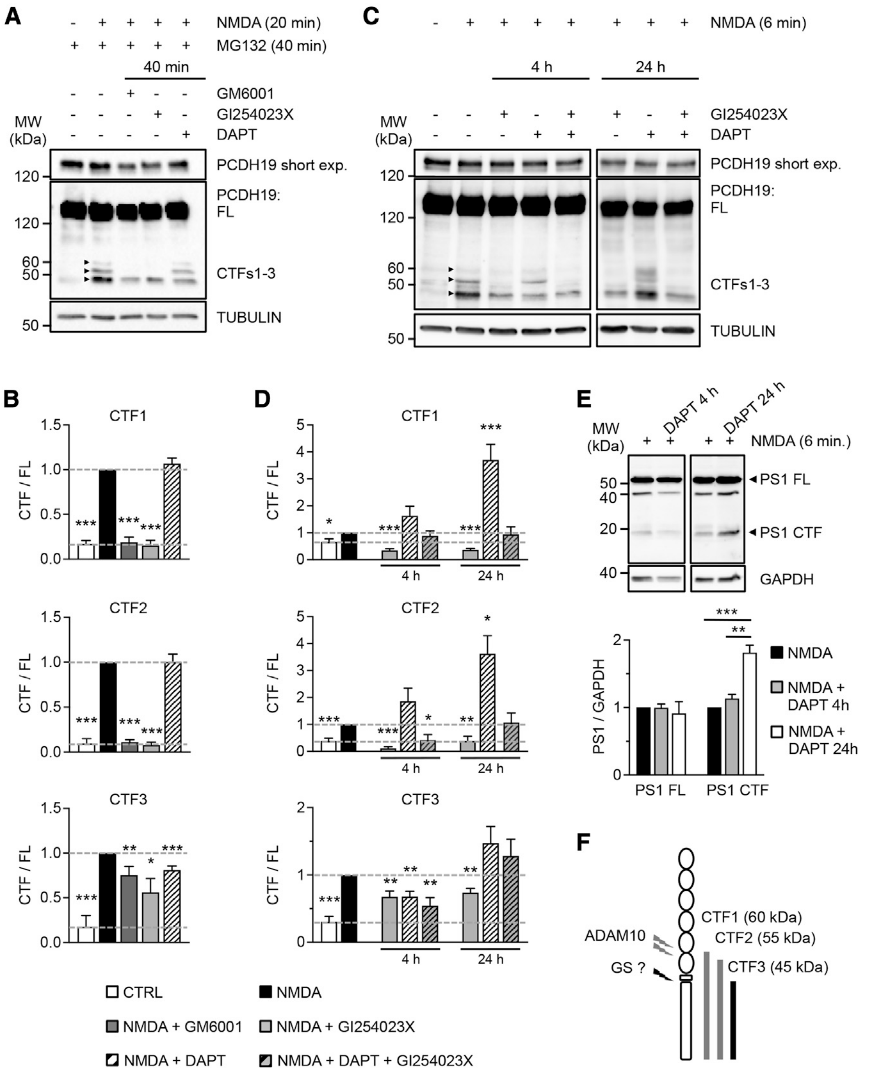

Figure 3. PCDH19 proteolytic cleavage is mediated by ADAM10 and possibly gamma secretase

(A) Western blot of neurons treated with NMDA, MG132, and the proteases inhibitors GM6001, GI254023X, and DAPT, as indicated. Two different exposures are shown for PCDH19 FL, a short (short exp.) one and a long one.

(B) Quantification of PCDH19 CTFs from neurons in (A). All CTF levels (CTF/FL) were increased by NMDA (n = 3–8, ***p < 0.001, Student's t test, CTRL compared with NMDA) and were significantly reduced by inhibitors of metalloproteases (n = 3–6; *p < 0.05, **p < 0.01, ***p < 0.001, Student's t test, NMDA + GM6001 or NMDA + GI254023X compared with NMDA). Only the CTF3 level was reduced by the inhibitor of gamma secretase (n = 5–8; ***p < 0.001, Student's t test, NMDA + DAPT compared with NMDA). Data are presented as mean ± SEM.

(C) Representative western blot of neurons treated with NMDA and proteases inhibitors, as indicated. Two different exposures are shown for PCDH19 FL, a short (short exp.) one and a long one. Protease inhibitors were applied for 4 or 24 h before NMDA treatment (6 min).

(D) Quantification of PCDH19 CTFs (CTFs/FL) from neurons in (C). All CTFs increased following NMDA treatment (n = 4–9; *p < 0.05, ***p < 0.001, Student's t test, CTRL compared with NMDA). All CTFs decreased following 4 or 24 h of treatment with GI254023X (n = 4–6; **p < 0.01, ***p < 0.001, Student's t test, NMDA + GI254023X compared with NMDA). The 4 h treatment with DAPT caused a decrease in CTF3 (n = 5; **p < 0.01, Student's t test, NMDA + DAPT compared with NMDA). The 24 h treatment with DAPT caused an increase in CTF1 and CTF2 (n = 7–9; *p < 0.05, ***p < 0.001, Student's t test, NMDA + DAPT compared with NMDA). Co-administration of GI254023X with DAPT reduced CTF3 levels at 4 h (n = 4; **p < 0.01, Student's t test, NMDA + DAPT + GI254023X compared with NMDA) and prevented DAPT-mediated changes in CTF1 and CTF2 levels at 24 h. Data are presented as mean ± SEM.

(E) Top panel, western blot of neurons treated with NMDA or NMDA + DAPT showing full-length presenilin-1 (PS1 FL) and presenilin-1 C-terminal fragment (PS1 CTF) expression. DAPT was applied for 4 or 24 h before NMDA treatment (6 min), as indicated. Bottom panel, quantification of PS1 FL and PS1 CTF. The 24 h treatment with DAPT caused a significant increase in PS1 CTF expression level (PS1 CTF/GAPDH, n = 3; **p < 0.01, ***p < 0.001, one-way ANOVA and Tukey's post hoc test). Data are presented as mean ± SEM.

(F) Scheme of PCDH19 proteolytic cleavage and resulting fragments (CTF1-3). GS, gamma secretase. Values are shown in Table S3.

See also Figure S3 and Table S8.

In a second set of experiments, we prolonged pretreatments with protease inhibitors to 4 and 24 h. In these experiments, we also shortened NMDA treatment to 6 min and did not apply MG132 since we were still able to appreciate NMDA-dependent CTFs increases under these conditions. We reconfirmed a strong decrease of CTF1 and CTF2 in the presence of the ADAM10 inhibitor GI254023X at both time points. By contrast, the gamma secretase inhibitor DAPT caused an upward trend in CTF1 and CTF2 at 4 h, which became statistically significant at 24 h and was prevented by the co-administration of GI254023X (Figures 3C and 3D). Four h treatments with GI254023X or DAPT caused a reduction in CTF3 by approximately 30% (Figures 3C and 3D), comparable to that observed with a different gamma secretase inhibitor, L-685,458 (Figures S3A and S3B). CTF3 reduction reached about 45% in neurons treated with DAPT + GI254023X (Figures 3C and 3D). Based on the partial reduction of CTF3 following ADAM10 and gamma secretase inhibition, we could not rule out the involvement of additional proteases, with the exception of caspases that can be activated by NMDA (Li et al., 2010) but did not affect CTF3 levels (Figures S3C and S3D).

While CTF3 reduction was still observed after the 24 h treatment with GI254023X, intriguingly, the 24 h treatment with DAPT tended to increase CTF3, although the increase did not reach the statistical significance (Figures 3C and 3D). It has been reported that prolonged DAPT treatments can provoke a

CellPress
OPEN ACCESS

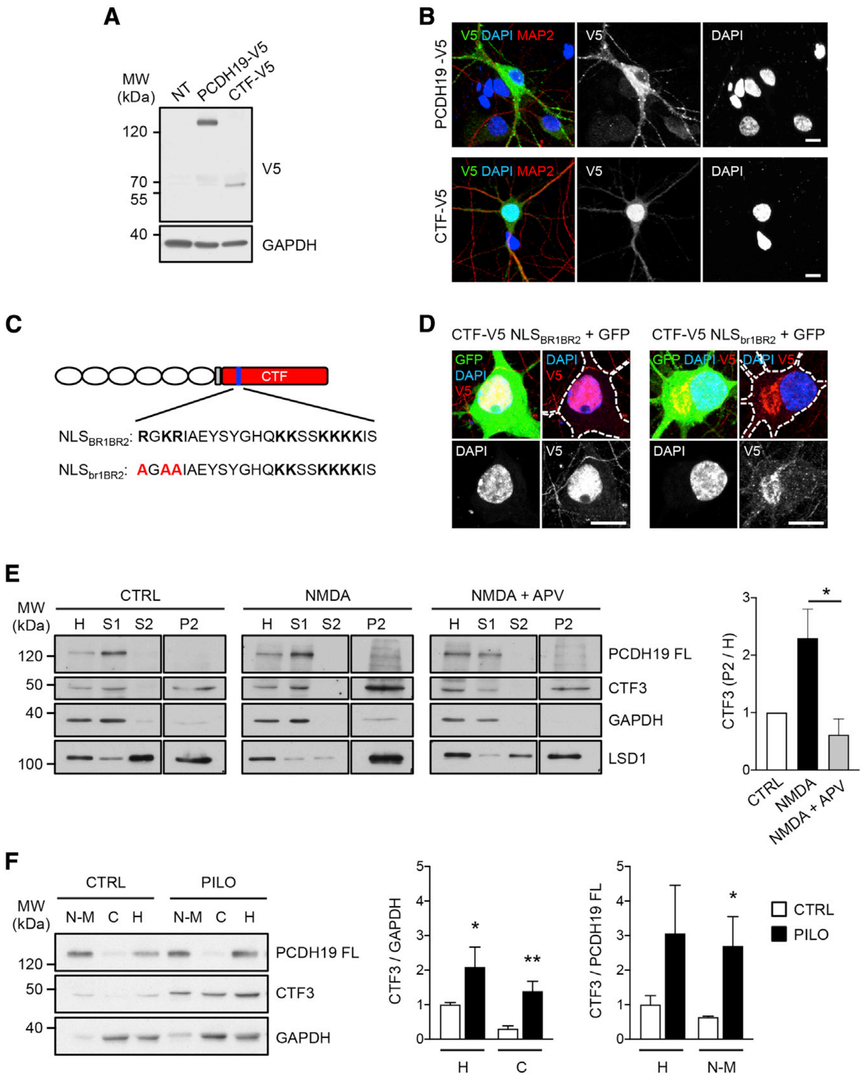

Figure 4. PCDH19 CTF harbors a functional nuclear localization signal (NLS) and enters the nucleus

(A) Western blot of primary hippocampal neurons either untransduced (NT) or transduced with PCDH19-V5 or CTF-V5.

(B) Hippocampal neurons transfected with PCDH19-V5 or CTF-V5 and stained with V5, MAP2, and DAPI. Scale bar, 10 μm.

(C) Scheme of PCDH19 structure, showing PCDH19 CTF (in red) and the NLS (in blue). The amino acids composing the two basic regions of the bipartite NLS (NLS_{BR1BR2}; BR, basic region) are in bold, with the mutated amino acids in red.

(D) ICC images of neurons transfected with the wild-type CTF-V5 + pEGFP (CTF-V5 NLS_{BR1BR2} + GFP) or CTF-V5 with mutated NLS + pEGFP (CTF-V5 NLS_{br1BR2} + GFP) and stained with V5 and DAPI. Scale bar, 10 μm.

(E) Fractionation of hippocampal neurons treated with vehicle (CTRL), NMDA, or NMDA + APV, and quantification of PCDH19 CTF3 enrichment in the P2 fraction normalized on CTRL (n = 3; *$p < 0.05$, one-way ANOVA and Tukey's post hoc test). GAPDH and LSD1 are used as cytoplasmic and nuclear markers, respectively. Data are presented as mean ± SEM. H, total homogenate; S1, cytosol and membrane-enriched fraction; S2, soluble nuclear fraction; P2, nuclear aggregates fraction.

(F) Western blot of brain-slice fractions from adult mice either untreated (CTRL) or treated with pilocarpine (PILO), and quantification of CTF3 normalized on GAPDH (n = 3–5; *$p < 0.05$, **$p < 0.01$, Student's t test) and PCDH19 FL (n = 3–5; *$p < 0.05$, Student's t test). N-M, nuclei-membranes enriched fraction; C, cytosolic fraction; H, total homogenate. Data are presented as mean ± SEM. Values are shown in Table S4.

See also Figure S4 and Table S9.

rebound effect associated with the increase of presenilin-1 (PS1) fragments (Sogorb-Esteve et al., 2018), which derive from PS1-regulated endoproteolysis and associate to form the active form of the enzyme (Brunkan et al., 2005). By using an antibody able to recognize the full-length PS1 (PS1 FL) and its active CTF (PS1 CTF), we observed a significant increase in PS1 CTF after a 24 h DAPT treatment, while PS1 FL was unaffected (Figure 3E). An increased enzymatic activity of PS1 and the accumulation of CTF1/CTF2 could explain the failure of prolonged DAPT treatment to decrease CTF3, provided, however, that DAPT is inactivated within the 24 h incubation period. Among possible alternatives, the emergence of other proteolytic cleavage pathways, as a consequence of prolonged inhibition of gamma secretase, cannot be excluded.

In conclusion, we demonstrate that PCDH19 undergoes NMDA-dependent proteolytic cleavage, which generates three C-terminal protein fragments, CTF1–3. Our results are consistent with a model whereby CTF1 and CTF2 are the membrane-bound protein stumps generated by ADAM10, while CTF3's origin appears more controversial. We hypothesize that CTF3 might be the cytosolic fragment deriving from CTF1 and CTF2, as result of a second cleavage step by gamma secretase (Figure 3F). However, further studies are needed to confirm this hypothesis and elucidate in full the proteolytic processing of PCDH19, which might involve additional proteases.

PCDH19 CTF enters the nucleus

We reasoned that the proteolytic cleavage of PCDH19 might explain the NMDA-dependent increase of PCDH19 signal in the nucleus (Figure 1 and S1), provided that PCDH19 CTF3 is able to enter the nucleus once released from the plasma membrane.

Since the gamma secretase cleavage sites lack a consensus motif (Wolfe and Kopan, 2004) and the cleavage site in PCDH19 is unknown, we cloned the entire human PCDH19 CTF (from amino acid [aa] 700) with a C-terminal V5 tag (CTF-V5) and transfected it into neurons (Figures 4A and 4B). We noticed that the apparent molecular weight of CTF-V5 in western blotting is higher

than that of CTF3. Partially, this is due to the V5 tag, and for the rest, it might be due to different post-translational modifications in human and rat PCDH19 cytoplasmic domains. Alternatively, we cannot exclude that rat PCDH19 CTF might undergo further processing, which results in a smaller CTF3.

When expressed in neurons, the PCDH19 FL (PCDH19-V5) and its cytoplasmic domain (CTF-V5) showed a complementary distribution: PCDH19-V5 was enriched in the perinuclear region and along dendrites, while CTF-V5 was enriched in the nucleus (Figure 4B). Similarly, in HEK293 cells, the entire human PCDH19 CTF (HA-CTF) and its N-terminal portion (HA-CTFa), which retain the nuclear localization signal (NLS), localized in the nucleus. By contrast, the C-terminal portion of the CTF (HA-CTFb) diffused in the cytoplasm (Figures S4A and S4B).

Consistently with the distribution of PCDH19 CTF, amino-acid sequence analysis revealed that it harbors a putative bipartite NLS composed by two regions enriched in basic amino acids (basic regions [BRs]) and located about 60 amino acids downstream of the transmembrane region (NLS_{BR1BR2}, aa 760–782 "**R**G**KR**IAEYSYGHQ**KK**SS**KKKK**IS", basic amino acids are in bold). To verify whether this NLS was responsible for CTF nuclear localization, we mutated the first stretch of basic amino acids (**R**G**KR**) into alanines (AGAA) and transfected the mutated construct (CTF-V5 NLS_{br1BR2}) in HEK cells and neurons. As expected, CTF-V5 NLS_{br1BR2} was excluded from the nucleus in both cell types (Figures 4C, 4D, S4A, and S4C).

To get evidence of the nuclear localization of the endogenously expressed PCDH19 CTF, we fractionated neurons treated with vehicle (CTRL), NMDA, and NMDA + APV to obtain the cytosol and membrane-enriched fraction (S1) and two nuclear fractions: the soluble one (S2) and the nuclear-aggregate-enriched one (P2). Notably, while PCDH19 FL was present exclusively in the total lysate (H) and S1 fraction, CTF3 was also detectable in the P2 fraction, at low levels in CTRL and NMDA + APV conditions, and at significantly higher levels in NMDA-treated neurons (Figure 4E). Importantly, PCDH19 proteolytic cleavage and CTF3 nuclear localization were also observed *in vivo* in mouse hippocampus. This occurred in response to pilocarpine, which induces glutamate-mediated synaptic activation and seizures (Smolders et al., 1997), as demonstrated by CTF3 increase in different cellular fractions, including the nuclei-membrane-enriched fraction (N-M; Figure 4F).

Taken together, these results demonstrate that PCDH19 CTF harbors a functional NLS and can reach the nucleus, both *in vitro* and *in vivo*.

PCDH19 CTF associates with the transcriptional corepressor complex LSD1/CoREST/HDAC2 and with chromatin

To get insights into PCDH19 function in the nucleus, we decided to investigate the hypothetical crosstalk between PCDH19 and the chromatin remodeler LSD1. LSD1 activity is influenced by NMDAR (Longaretti et al., 2020), and LSD1 controls neuronal excitability and is involved in neurodevelopmental disorders with DEE9 overlapping phenotypes (Pilotto et al., 2016; Rusconi et al., 2015).

Co-immunoprecipitation (coIP) experiments in HEK cells demonstrated that PCDH19-V5 is able to bind both the ubiquitous LSD1 isoform (HA-LSD1) and its neuronal-specific isoform neuroLSD1 (HA-nLSD1), but not the nuclear protein NOVA1 (HA-NOVA1), used as a negative control (Figure 5A). Furthermore, PCDH19 CTF (CTF-V5) was co-immunoprecipitated by both LSD1 isoforms but not by NOVA1 (Figure S4D), while the removal of the distal portion of PCDH19 CTF (PCDH19-879Δ) prevented the coIP (Figure S4E). Analogously, no coIP was detected between LSD1 (HA-LSD1 or HA-nLSD1) and another protocadherin, PCDH9, which shares with PCDH19 the conserved regions CM1 and CM2 (Figure S4E). Taken together, these data indicate that PCDH19 and LSD1 can interact, and that the region of PCDH19 involved is the CTF portion downstream of amino acid 879, while CM1 and CM2 are not required, thus suggesting the specificity of the interaction.

To verify whether PCDH19 and LSD1 can associate in brain tissue, we performed a GST pull down assay in rat brain homogenates and reconfirmed the data obtained in heterologous cells. GST-CTF was able to pull down CoREST, SRF/SRFΔ5, and HDAC2 that, together with LSD1, belong to the CoREST complex (Gerosa et al., 2020). By contrast, no association was detected with NOVA1, a nuclear protein that does not belong to the complex (Figure 5B).

These results prompted us to investigate whether PCDH19 CTF might also associate with chromatin. To this end, we performed chromatin immunoprecipitation (ChIP) experiments in homogenates from mouse hippocampal slices by using an antibody against LSD1 or PCDH19 CTF. Among the chromatin fragments precipitated by anti-LSD1 and anti-PCDH19, we looked for promoters of selected LSD1 target IEGs, namely *Nr4a1*, *c-Fos*, and *Npas4* (Rusconi et al., 2016; Wang et al., 2015). Notably, qRT-PCR indicated a significant enrichment of all these gene promoters with both antibodies (Figure 5C).

Altogether, these data indicate that PCDH19 CTF associates with the LSD1 complex and with chromatin in correspondence of known LSD1 target IEGs. Importantly, PCDH19 CTF binding with chromatin can be detected under basal conditions.

PCDH19 regulates the expression of IEGs

The association of PCDH19 CTF with the LSD1 complex and chromatin prompted us to investigate whether PCDH19 might contribute to gene-expression regulation in hippocampal neurons.

To this end, we focused on a panel of IEGs known to be LSD1 targets, namely *Nr4a1*, *c-Fos*, *Egr1*, *Cyr61*, and *Npas4* (Wang et al., 2015), and we measured their expression following PCDH19 CTF (CTF-V5) overexpression or short hairpin RNA (shRNA)-mediated PCDH19 downregulation (Figures 6A–6C, S5A, and S5B). Notably, CTF-V5 overexpression was sufficient to cause a significant decrease of *Nr4a1*, *c-Fos*, and *Npas4* expression, while *Egr1* expression did not change, and *Cyr61*-expression changes did not reach statistical significance compared with control neurons (Figure 6A). Conversely, shRNA-mediated PCDH19 downregulation was associated with an increased expression of *Nr4a1*, *Egr1*, and *Npas4*, while *c-Fos* and *Cyr61* did not change compared with control neurons expressing a control shRNA (scramble; Figure 6B). It is interesting to note that both conditions, i.e., PCDH19 CTF overexpression and PCDH19 downregulation, had a significant effect

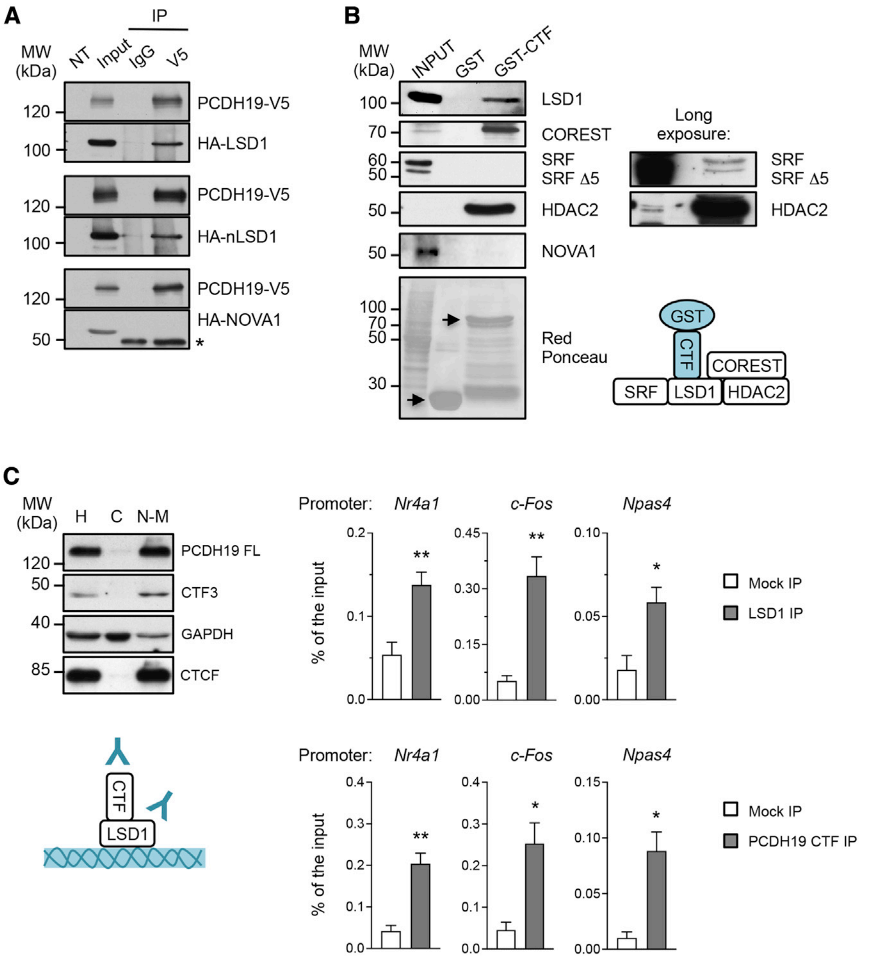

Figure 5. PCDH19 associates with the LSD1 gene-expression regulatory complex and with chromatin

(A) coIP in HEK cells co-transfected with PCDH19-V5 and HA-LSD1, HA-nLSD1, or HA-NOVA1, as indicated. Input: 10% of IP volume. IP: anti-IgG or anti-V5. Western blots probed for V5 or HA. The star indicates IgG chain signals. NT, lysate from untransfected cells.

(B) GST pull down assay in adult rat cortex and hippocampus. GST is used as negative control. Input: 10%. Red ponceau shows GST and GST-CTF (arrows).

(C) Left, western blot of subcellular fractions from mouse brain slices. H, total homogenate; C, cytosolic fraction; N-M, nuclei-membranes enriched fraction. Right, ChIP on homogenates of mouse brain slices. ChIP was performed on *Nr4a1*, *c-Fos*, and *Npas4* promoters with anti-LSD1 antibody (top right panel, n = 4; *p < 0.05, **p < 0.01, Student's t test) and anti-PCDH19 CTF antibody (bottom right panel, n = 3; *p < 0.05, **p < 0.01, Student's t test). Data are presented as mean ± SEM. Values are shown in Table S5.

See also Figure S4 and Table S9.

on 3 out of 5 IEGs tested but in opposite directions, as the former was associated with reduced—whereas the latter with increased—gene expression. *Nr4a1* and *Npas4* were targeted by both CTF-V5 and shRNA, while *c-Fos* and *Egr1* were affected specifically by CTF-V5 overexpression and shRNA condition, respectively (Figures 6A and 6B).

We further investigated the effect of PCDH19 at the protein level of NR4A1. We measured the amount of NR4A1 by ICC in neurons transfected with control shRNA (scramble), wild-type (CTF-V5 NLS$_{BR1BR2}$) or mutant (CTF-V5 NLS$_{br1BR2}$) CTF-V5, PCDH19 shRNA, or PCDH19 shRNA + PCDH19-V5 (rescue) treated with either vehicle, NMDA, or DAPT. As expected, NR4A1 fluorescent signal increased in response to NMDA. Notably, the overexpression of the wild-type CTF-V5, but not of the CTF-V5 with mutant NLS, fully prevented the NMDA-induced increase of NR4A1. Conversely, PCDH19 downregulation was sufficient to increase NR4A1 expression without NMDA application. The co-transfection of PCDH19 shRNA with PCDH19-V5 was able to restore NR4A1 expression levels to control levels, provided that neurons had not been treated with DAPT, thus suggesting that the rescue effect of PCDH19 on NR4A1 expression requires gamma secretase activity (Figure 6C).

Altogether, these data uncover the role of PCDH19 in regulating gene expression, in particular a subset IEGs, with potential implications for neuronal excitability and plasticity.

PCDH19 downregulation affects LSD1 alternative splicing

In neurons, the epigenetic activity of LSD1 crucially depends on the relative amount of LSD1 and its neuron-specific alternative isoform neuroLSD1, whose generation is promoted by the splicing factor NOVA1 together with nSR100 (Rusconi et al., 2015). In fact, neuroLSD1 acts as a dominant-negative isoform, which promotes corepressor-complex disassembly and derepression of LSD1 target genes (Toffolo et al., 2014). For this reason, we measured the LSD1/neuroLSD1 ratio in neurons following PCDH19 CTF overexpression or shRNA-mediated downregulation. Notably, while CTF overexpression did not alter the LSD1/neuroLSD1 ratio, PCDH19 downregulation significantly increased the proportion of neuroLSD1 (Figure 6D), as well as NOVA1 expression, compared with control neurons (Figure 6E).

These data are consistent with the increased expression of IEGs observed in neurons expressing PCDH19 shRNA (Figures 6B and 6C) and provide an additional mechanism by which PCDH19 can affect IEG expression via LSD1 complex.

The nuclear role of PCDH19 is conserved in human neurons

To extend the validity of our results to human neurons, we took advantage of neurons derived from human-induced pluripotent stem cells (hiPSCs). Western blot analysis of lysates from hiPSC-derived neurons (Frega et al., 2017) revealed a major

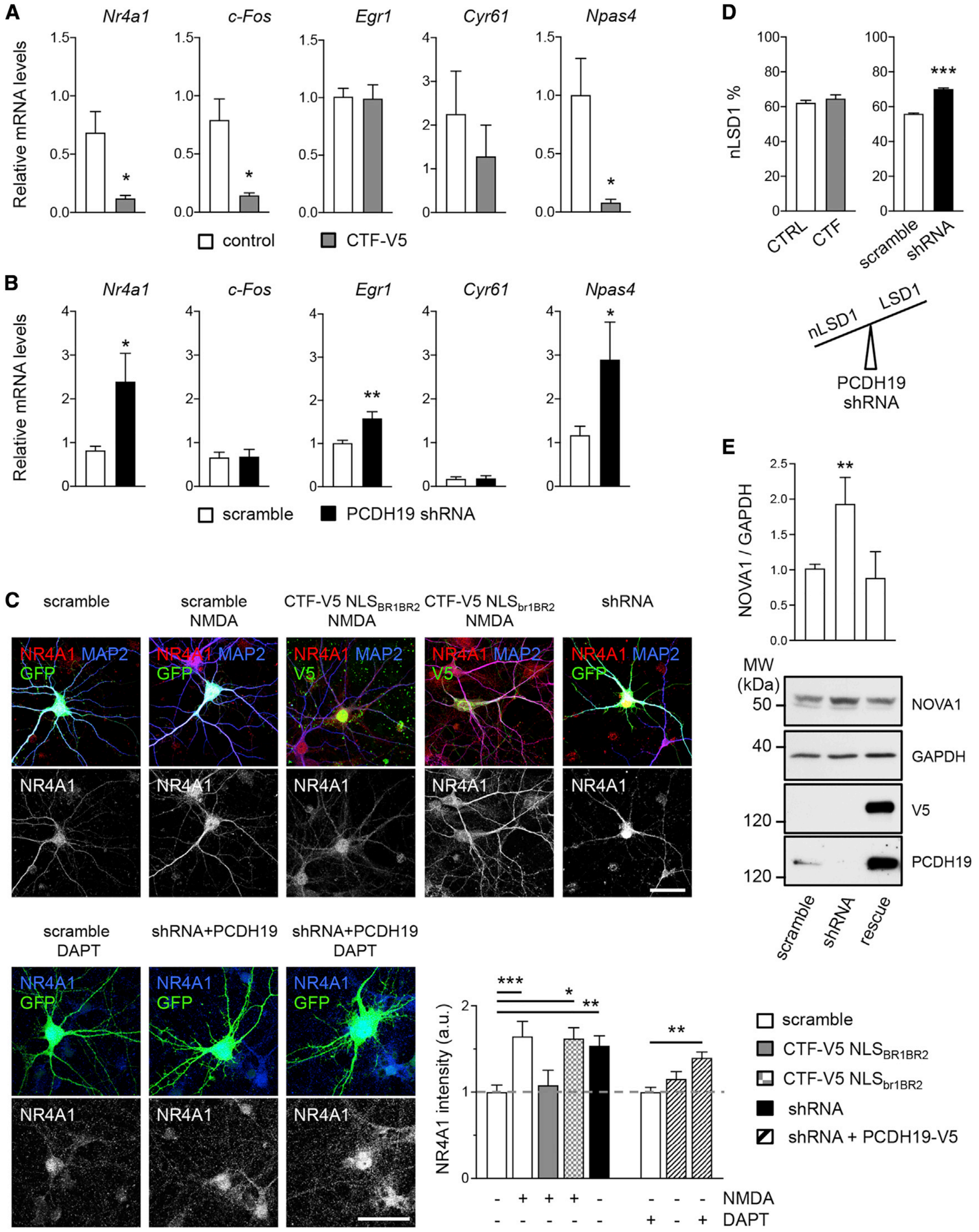

Figure 6. PCDH19 regulates IEG expression and LSD1 alternative splicing

(A) RT-PCR in hippocampal neurons transduced with PCDH19 CTF (CTF-V5) or empty vector as control. The relative mRNA level of *Nr4a1*, *c-Fos*, *Egr1*, *Cyr61*, and *Npas4* is shown (n = 3–4; *p < 0.05, Student's t test). Data are presented as mean ± SEM.

(B) RT-PCR in hippocampal neurons transduced with PCDH19 shRNA or control shRNA (scramble). The relative mRNA level of *Nr4a1*, *c-Fos*, *Egr1*, *Cyr61*, and *Npas4* is shown (n = 6–8; *p < 0.05, **p < 0.01, Student's t test). Data are presented as mean ± SEM.

(C) Left panels, confocal images of hippocampal neurons transfected with control shRNA (scramble), CTF-V5 NLS$_{BR1BR2}$, CTF-V5 NLS$_{br1BR2}$, PCDH19 shRNA (shRNA), and PCDH19 shRNA + PCDH19-V5 (shRNA + PCDH19, rescue condition) and treated with vehicle, NMDA, or DAPT (4 h) before immunolabeling for NR4A1, MAP2, and V5, as indicated. Scale bars, 40 μm. Bottom right panel, quantification of NR4A1 fluorescence intensity (entire neuron) (n, scramble 58,

(legend continued on next page)

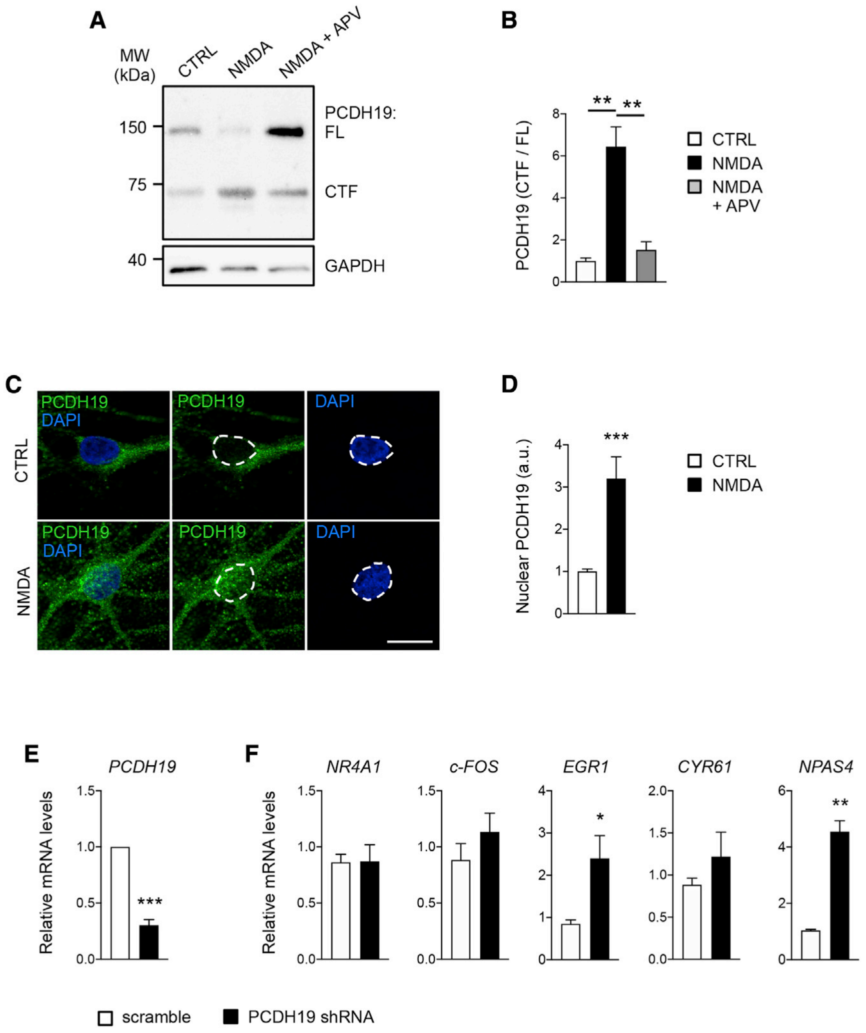

Figure 7. PCDH19 nuclear role in hiPSC-derived neurons

(A) Western blot of neurons treated with vehicle (CTRL) or NMDA, in presence of APV or not, as indicated.

(B) Quantification of PCDH19 (CTF/FL) in western-blot experiments shown in (A) (n = 3; **p < 0.01, one-way ANOVA and Tukey's post hoc test). Data are presented as mean ± SEM.

(C) Representative images of hiPSC-derived neurons stained for PCDH19 and DAPI, showing PCDH19 distribution after NMDAR stimulation. Scale bar, 10 μm.

(D) Quantification of PCDH19 nuclear expression in ICC experiments shown in (C) (n = 12; ***p < 0.001, Student's t test). Data are presented as mean ± SEM.

(E) RT-PCR in hiPSC-derived neurons transduced with PCDH19 shRNA or control shRNA (scramble). The relative mRNA level of *PCDH19* is shown (n = 3; ***p < 0.001, Student's t test). Data are presented as mean ± SEM.

(F) RT-PCR in hiPSC-derived neurons infected with PCDH19 shRNA or control shRNA (scramble). The relative mRNA level of *NR4A1*, *c-FOS*, *EGR1*, *CYR61*, and *NPAS4* is shown (n = 4–8; *p < 0.05, **p < 0.01, Student's t test). Data are presented as mean ± SEM. Values are shown in Table S7.
See also Table S9.

band, corresponding to PCDH19 FL, and a minor band (CTF) with an apparent molecular weight consistent with that of the human PCDH19 CTF when overexpressed in rat neurons (Figure 7A). The PCDH19 CTF/FL ratio significantly increased following NMDA, but not NMDA + APV, treatment (Figure 7B). Furthermore, upon NMDA treatment, the PCDH19 nuclear signal increased, as evaluated by ICC with an antibody against its C-terminal domain (Figures 7C and 7D), thus confirming the NMDA-dependent processing of PCDH19 in human neurons.

Since PCDH19 mutations include whole-gene deletions and pathogenic variants are expected to cause PCDH19 loss of function (Niazi et al., 2019), we analyzed the effect of PCDH19 downregulation (Figure 7E) on IEG expression in hiPSC-derived neurons. We observed a significant increase in *EGR1* and *NPAS4*. Except for *NR4A1*, which did not change, these data recapitulated those obtained in rat neurons (Figure 7F), thus indicating that the regulatory role of PCDH19 on IEG expression is largely conserved from rodents to humans.

DISCUSSION

This study reports a synapse-to-nucleus signaling pathway through which the synaptic adhesion molecule PCDH19 bridges neuronal activity with IEG expression through epigenetic regulation. In particular, the identified pathway relies on the proteolytic cleavage of PCDH19 by ADAM10 and possibly gamma secretase, which generates an intracellular CTF able to enter the

scramble + NMDA 32, CT-V5 NLS_{BR1BR2} + NMDA 20, CTF-V5 NLS_{br1BR2} + NMDA 16, shRNA 35, scramble + DAPT 15, shRNA + PCDH19 17, shRNA + PCDH19 + DAPT 14; *p < 0.05, **p < 0.01, ***p < 0.001, one-way ANOVA and Tukey's post hoc test). Data are presented as mean ± SEM.
(D) Quantification of neuroLSD1 (nLSD1) isoform (%) in hippocampal neurons transduced as in (A) and (B) (n = 6, ***p < 0.001, Student's t test). Data are presented as mean ± SEM.
(E) Quantification of NOVA1 expression in western blots (top) and representative western blot (bottom) from hippocampal neurons transfected with control shRNA (scramble), PCDH19 shRNA (shRNA), and shRNA + PCDH19-V5 (rescue) (n = 3–4; **p < 0.01, one-way ANOVA and Dunnett's post hoc test). Data are presented as mean ± SEM. Values are shown in Table S6.
See also Figure S5 and Tables S8 and S9.

nucleus and modulate IEG expression via crosstalk with the epigenetic enzyme LSD1.

Interestingly, PCDH19 cleavage and CTF nuclear localization appear to occur constitutively, as demonstrated by the presence of CTF in homogenates and nuclei-enriched fractions from both primary neurons and brain tissue and the association of CTF to chromatin under basal conditions. Nonetheless, PCDH19 cleavage is strongly enhanced specifically by NMDARs in both developing and mature primary neurons and *in vivo* in mice by pilocarpine, whose convulsant effects are initiated via muscarinic receptors and further mediated via NMDARs (Smolders et al., 1997). PCDH19, ADAM10, and gamma secretase have all been detected at synapses (Hayashi et al., 2017; Marcello et al., 2007; Schedin-Weiss et al., 2016), where TIF fractionation assays suggest that PCDH19 cleavage takes place. This is consistent with the increase of PCDH19 nuclear signal in response to synaptic—but not extrasynaptic—NMDAR activation. Altogether, PCDH19 emerges as a protein that mediates a constitutive synapses-to-nucleus information flux, modulated by neuronal activity.

There is increasing evidence in favor of the engagement by synaptic activity of epigenetic mechanisms to implement transcriptional patterns that will, in turn, shape synaptic structure and function (Campbell and Wood, 2019). In support of this view, we found that PCDH19 CTF associates with the chromatin remodeler LSD1 and its gene-expression regulatory complex (Rusconi et al., 2016). Notably, CTF overexpression in hippocampal neurons was sufficient to downregulate the expression of selected LSD1 target IEGs, namely *Nr4a1*, *c-Fos*, and *Npas4*, and to fully prevent NMDA-mediated *Nr4a1* upregulation. These results are consistent with a model whereby PCDH19 CTF cooperates with LSD1 complex to repress IEGs expression, thus exerting a homeostatic negative feedback that might prevent runaway excitation and neuron hyperactivity.

It is interesting to note that altered proteolytic processing has been associated with neurological disorders, including schizophrenia, and altered protease expression and activity to fragile X syndrome and epilepsy (Nagappan-Chettiar et al., 2017). We hypothesize that PCDH19 proteolytic processing and CTF signaling might also be altered in DEE9. In fact, PCDH19 cleavage and CTF generation would be prevented in the presence of *PCDH19* whole-gene deletions and truncating variants (Depienne et al., 2011; Kolc et al., 2018). We speculate that extracellular missense point mutations that impair adhesiveness (Niazi et al., 2019) might also affect PCDH19 processing, since adhesion and protein-protein interactions are known to modulate proteolytic processing, by either promoting or preventing it (Nagappan-Chettiar et al., 2017). For the same reason, heterozygous gene mutations and the resulting PCDH19 mosaic expression, which has been shown to introduce trans-synaptic mismatches between PCDH19-NCAD complexes with a deleterious effect on synapses (Hoshina et al., 2021), might also theoretically affect PCDH19 proteolytic processing.

Here, we mimicked PCDH19 loss of function by shRNA-mediated downregulation. We recently reported that PCDH19 shRNA-expressing neurons are hyperexcitable (Serratto et al., 2020). Consistently, we observed a general increase of IEG expression following PCDH19 downregulation, contrary to what observed with CTF overexpression. Mechanistically, IEGs' increased expression can occur in two non-exclusive ways: (1) the loss of CTF nuclear signaling and its physical association with LSD1, and (2) the upregulation of neuroLSD1 isoform via the splicing factor NOVA1. Interestingly, LSD1 splicing was specifically affected by PCDH19 shRNA but not by CTF overexpression. This might explain why some IEGs, namely *Nr4a1* and *Npas4*, were oppositely regulated by PCDH19 CTF and shRNA, while others (*c-Fos* and *Egr1*) were specifically affected by one of the two. In fact, neuroLSD1 isoform, in addition to counteracting LSD1 repressor function, also displays different substrate specificities, which allows a differential control of gene expression (Laurent et al., 2015).

Notably, upregulation of NOVA1, neuroLSD1, and of the IEG proteins NR4A1, EGR1, and NPAS4 has been observed in patients with epilepsy and/or in epileptic mouse models (van Loo et al., 2019; Rusconi et al., 2015, 2017; Shan et al., 2018; Zhang et al., 2016), and their deregulation might contribute to DEE9 epileptic phenotype. Concerning NPAS4, it has been proposed to function as a molecular switch to initiate homeostatic scaling (Shan et al., 2018) and might therefore reflect the attempt of neurons to counteract hyperexcitability.

Importantly, PCDH19 downregulation was also associated with increased IEG expression in hiPSC-derived neurons. Despite key differences in the experimental models, *in primis*, the neuronal type (cortical versus hippocampal neurons), the absence of inhibitory neurons in human hiPSC-derived neuronal cultures, and the timing of PCDH19 downregulation (DIV 8-12 in rat neurons and DIV 1-35 in hiPSC-derived neurons), still, the increase of IEGs was observed and reached statistical significance for *Egr1* and *Npas4*. This provides an important indication about the conservation of the PCDH19 nuclear role in humans.

In conclusion, our study provides evidence in favor of the deep crosstalk between synaptic activity and epigenetics exerted via the proteolytic processing of the CAM PCDH19. Furthermore, PCDH19 homeostatic function in neurons provides a key to interpret DEE9 hyperexcitability and associated symptoms.

Limitations of the study

In this study, we address PCDH19 proteolytic cleavage and its nuclear role in regulating IEGs expression. We acknowledge the following main limitations.

We propose a model whereby CTF3 might be the cytosolic fragment deriving from the ADAM10 products CTF1 and CTF2 as result of a second cleavage step by gamma secretase. While being supported by the partial reduction of CTF3 following inhibition of ADAM10 and gamma secretase and by the effect of DAPT on PCDH19-mediated rescue of NR4A1 expression in shRNA expressing neurons, this hypothesis is challenged by the modest extent of CTF3 reduction (less than 50% in all the experimental conditions tested) and CTF1–2-level patterns following gamma secretase inhibition. CTF1 and CTF2 levels were unaffected by the 40 min DAPT treatment, showed an upward trend after 4 h of DAPT—but not L-685,458—treatment, and increased significantly only after 24 h of DAPT treatment. The chance to readily detect gamma-secretase-substrate accumulation might be affected by protein fragment instability and degradation, which should be addressed. Nonetheless, in view

of these considerations, the involvement of gamma secretase in the processing of PCDH19, though anticipated, could not be confirmed by our analyses.

While reporting PCDH19's nuclear role, this study considers a few selected IEGs. It would be valuable to learn about the impact at the genome-wide level of PCDH19, following its constitutive and NMDA-induced cleavage. In fact, each IEG regulates several downstream genes, thus creating the basis for a broad PCDH19 transcriptomic effect.

Although we observed PCDH19 proteolytic cleavage in pilocarpine-treated mice, we currently do not know about the *in vivo* relevance of our results, as this study was mainly conducted *in vitro* on cultured neurons of both rodent and human origin.

The function of PCDH19 soluble ectodomain, generated by ADAM10 together with CTF1 and CTF2, was beyond the scope of this study but remains an intriguing open question. PCDH19 ectodomain might exert a signaling role important for synapse development and function, as demonstrated for several adhesion molecules including cadherins, neuroligins, integrins, and immunoglobulins (Bemben et al., 2019; Nagappan-Chettiar et al., 2017; Pillai-Nair et al., 2005; Suzuki et al., 2012).

Finally, the role of PCDH19 processing and nuclear signaling in DEE9 is admittedly speculative, and the validation of this pathogenic hypothesis will have to take into account the peculiar inheritance pattern of DEE9, which mainly affects females with heterozygous mutations (Dibbens et al., 2008). While the cell interference (Depienne et al., 2011) was considered the only plausible explanation for this, a more complex scenario is currently emerging (Lim et al., 2019; Rakotomamonjy et al., 2020; Robens et al., 2021), which deserve examination.

STAR★METHODS

Detailed methods are provided in the online version of this paper and include the following:

- KEY RESOURCE TABLE
- RESOURCE AVAILABILITY
  - Lead contact
  - Materials availability
  - Data and code availability
- EXPERIMENTAL MODEL AND SUBJECT DETAILS
  - Primary hippocampal neurons
  - C57BL/6N mice
  - HEK cells
  - Human-induced pluripotent stem cells
- METHOD DETAILS
  - Plasmids
  - NLS prediction
  - Treatments on primary neurons
  - ICC, image acquisition and analysis
  - Biotinylation assay
  - Crude synaptosomes and TIF
  - Subcellular fractionation
  - GST pull-down
  - CoIP
  - Immunoblotting
  - ChIP
  - RT-PCR
- QUANTIFICATION AND STATISTICAL ANALYSIS

SUPPLEMENTAL INFORMATION

Supplemental information can be found online at https://doi.org/10.1016/j.celrep.2022.110857.

ACKNOWLEDGMENTS

This work was supported by Fondazione Cariplo (grant no. 2014-0972 to S.B. and F.R.), Fondazione LeJeune (grant no. 1945_2020 to M.P.), Insieme per la Ricerca PCDH19 – ONLUS (grant 2014 to M.P. and E.B.), and Fondazione Telethon – Italy (grant nos. GGP17260 and GGP20056 to S.B.). This work was also supported by grants from the Netherlands Organisation for Health Research and Development (ZonMW grants 91217055 to N.N.K). The purchase of the Zeiss LSM 800 confocal microscope was funded by Regione Lombardia (Amanda Project CUP_B42F16000440005) and Fondazione Antonio Carlo Monzino. We acknowledge Daniele di Marino (Polytechnic University of Marche, Ancona), Federico Miozzo (IN-CNR, Milano), and Antonio Zippo (IN-CNR, Milano) for valuable discussions.

AUTHOR CONTRIBUTIONS

Conceptualization, F.R., E.B., M.P., and S.B.; investigation, L.G., S.M., F.R., A.L., E.L., S.P., and S.B.; writing – original draft, S.B.; writing – review & editing, L.G., S.M., F.R., A.L., L.M., E.M., N.N.K., E.B., M.P., and S.B.; visualization, L.G., S.M., and S.B.; resources, V.B. and S.G.G.; supervision, L.M., V.B., E.M., N.N.K., E.B., M.P., and S.B.; funding acquisition, F.R., N.N.K., E.B., M.P., and S.B.

DECLARATION OF INTERESTS

The authors declare no competing interests.

Received: March 27, 2021
Revised: February 11, 2022
Accepted: May 1, 2022
Published: May 24, 2022

REFERENCES

Bassani, S., Cwetsch, A.W., Gerosa, L., Serratto, G.M., Folci, A., Hall, I.F., Mazzanti, M., Cancedda, L., and Passafaro, M. (2018). The female epilepsy protein PCDH19 is a new GABAAR-binding partner that regulates GABAergic transmission as well as migration and morphological maturation of hippocampal neurons. Hum. Mol. Genet. *27*, 1027–1038. https://doi.org/10.1093/hmg/ddy019.

Bemben, M.A., Nguyen, T.A., Li, Y., Wang, T., Nicoll, R.A., and Roche, K.W. (2019). Isoform-specific cleavage of neuroligin-3 reduces synapse strength. Mol. Psychiat. *24*, 145–160. https://doi.org/10.1038/s41380-018-0242-y.

Bonn, S., Seeburg, P.H., and Schwarz, M.K. (2007). Combinatorial expression of alpha- and gamma-protocadherins alters their presenilin-dependent processing. Mol. Cell Biol. *27*, 4121–4132. https://doi.org/10.1128/mcb.01708-06.

Bouillot, S., Tillet, E., Carmona, G., Prandini, M.H., Gauchez, A.S., Hoffmann, P., Alfaidy, N., Cand, F., and Huber, P. (2011). Protocadherin-12 cleavage is a regulated process mediated by ADAM10 protein. J. Biol. Chem. *286*, 15195–15204. https://doi.org/10.1074/jbc.m111.230045.

Bouza, A.A., Edokobi, N., Hodges, S.L., Pinsky, A.M., Offord, J., Piao, L., Zhao, Y.T., Lopatin, A.N., Lopez-Santiago, L.F., and Isom, L.L. (2021). Sodium channel β1 subunits participate in regulated intramembrane proteolysis-excitation coupling. JCI Insight *6*, e141776. https://doi.org/10.1172/jci.insight.141776.

Brunkan, A.L., Martinez, M., Walker, E.S., and Goate, A.M. (2005). Presenilin endoproteolysis is an intramolecular cleavage. Mol. Cell Neurosci. *29*, 65–73. https://doi.org/10.1016/j.mcn.2004.12.012.

Campbell, R.R., and Wood, M.A. (2019). How the epigenome integrates information and reshapes the synapse. Nat. Rev. Neurosci. *20*, 133–147. https://doi.org/10.1038/s41583-019-0121-9.

Clayton, D.F., Anreiter, I., Aristizabal, M., Frankland, P.W., Binder, E.B., and Citri, A. (2020). The role of the genome in experience-dependent plasticity: extending the analogy of the genomic action potential. Proc. Natl. Acad. Sci. U S A *117*, 23252–23260. https://doi.org/10.1073/pnas.1820837116.

Cooper, S.R., Emond, M.R., Duy, P.Q., Liebau, B.G., Wolman, M.A., and Jontes, J.D. (2015). Protocadherins control the modular assembly of neuronal columns in the zebrafish optic tectum. J. Cell Biol. *211*, 807–814. https://doi.org/10.1083/jcb.201507108.

Cooper, S.R., Jontes, J.D., and Sotomayor, M. (2016). Structural determinants of adhesion by Protocadherin-19 and implications for its role in epilepsy. Elife *5*, e18529. https://doi.org/10.7554/elife.18529.

Depienne, C., Trouillard, O., Bouteiller, D., Gourfinkel-An, I., Poirier, K., Rivier, F., Berquin, P., Nabbout, R., Chaigne, D., Steschenko, D., et al. (2011). Mutations and deletions in PCDH19 account for various familial or isolated epilepsies in females. Hum. Mutat. *32*, E1959–E1975. https://doi.org/10.1002/humu.21373.

Dibbens, L.M., Tarpey, P.S., Hynes, K., Bayly, M.A., Scheffer, I.E., Smith, R., Bomar, J., Sutton, E., Vandeleur, L., Shoubridge, C., et al. (2008). X-linked protocadherin 19 mutations cause female-limited epilepsy and cognitive impairment. Nat. Genet. *40*, 776–781. https://doi.org/10.1038/ng.149.

Du, W., Liu, X., Fan, G., Zhao, X., Sun, Y., Wang, T., Zhao, R., Wang, G., Zhao, C., Zhu, Y., et al. (2014). From cell membrane to the nucleus: an emerging role of E-cadherin in gene transcriptional regulation. J. Cell Mol. Med. *18*, 1712–1719. https://doi.org/10.1111/jcmm.12340.

Emond, M.R., Biswas, S., Blevins, C.J., and Jontes, J.D. (2011). A complex of Protocadherin-19 and N-cadherin mediates a novel mechanism of cell adhesion. J. Cell Biol. *195*, 1115–1121. https://doi.org/10.1083/jcb.201108115.

Frega, M., van Gestel, S.H.C., Linda, K., van der Raadt, J., Keller, J., Van Rhijn, J.R., Schubert, D., Albers, C.A., and Nadif Kasri, N. (2017). Rapid neuronal differentiation of induced pluripotent stem cells for measuring network activity on micro-electrode arrays. J. Vis. Exp. *119*, 54900. https://doi.org/10.3791/54900.

Gerosa, L., Grillo, B., Forastieri, C., Longaretti, A., Toffolo, E., Mallei, A., Bassani, S., Popoli, M., Battaglioli, E., and Rusconi, F. (2020). SRF and SRFΔ5 splicing isoform recruit corepressor LSD1/KDM1A modifying structural neuroplasticity and environmental stress response. Mol. Neurobiol. *57*, 393–407. https://doi.org/10.1007/s12035-019-01720-8.

Goldberg, A.D., Allis, C.D., and Bernstein, E. (2007). Epigenetics: a landscape takes shape. Cell *128*, 635–638. https://doi.org/10.1016/j.cell.2007.02.006.

Hayashi, S., Inoue, Y., Hattori, S., Kaneko, M., Shioi, G., Miyakawa, T., and Takeichi, M. (2017). Loss of X-linked Protocadherin-19 differentially affects the behavior of heterozygous female and hemizygous male mice. Sci. Rep. *7*, 5801. https://doi.org/10.1038/s41598-017-06374-x.

Hoshina, N., Johnson-Venkatesh, E.M., Hoshina, M., and Umemori, H. (2021). Female-specific synaptic dysfunction and cognitive impairment in a mouse model of PCDH19 Disord. Science *372*, eaaz3893.

Karpova, A., Mikhaylova, M., Bera, S., Bär, J., Reddy, P.P., Behnisch, T., Rankovic, V., Spilker, C., Bethge, P., Sahin, J., et al. (2013). Encoding and transducing the synaptic or extrasynaptic origin of NMDA receptor signals to the nucleus. Cell *152*, 1119–1133. https://doi.org/10.1016/j.cell.2013.02.002.

Kim, S., Kim, H., and Um, J.W. (2018). Synapse development organized by neuronal activity-regulated immediate-early genes. Exp. Mol. Med. *50*, 1–7. https://doi.org/10.1038/s12276-018-0025-1.

Kolc, K.L., Sadleir, L.G., Scheffer, I.E., Ivancevic, A., Roberts, R., Pham, D.H., and Gecz, J. (2018). A systematic review and meta-analysis of 271 PCDH19-variant individuals identifies psychiatric comorbidities, and association of seizure onset and disease severity. Mol. Psychiatry *24*, 241–251. https://doi.org/10.1038/s41380-018-0066-9.

Kosugi, S., Hasebe, M., Tomita, M., and Yanagawa, H. (2009). Systematic identification of cell cycle-dependent yeast nucleocytoplasmic shuttling proteins by prediction of composite motifs. Proc. Natl. Acad. Sci. U S A. *106*, 10171–10176. https://doi.org/10.1073/pnas.0900604106.

Laurent, B., Ruitu, L., Murn, J., Hempel, K., Ferrao, R., Xiang, Y., Liu, S., Garcia, B.A., Wu, H., Wu, F., et al. (2015). A specific LSD1/KDM1A isoform regulates neuronal differentiation through H3K9 demethylation. Mol. Cell *57*, 957–970. https://doi.org/10.1016/j.molcel.2015.01.010.

Li, Z., Jo, J., Jia, J.M., Lo, S.C., Whitcomb, D.J., Jiao, S., Cho, K., and Sheng, M. (2010). Caspase-3 Activation via Mitochondria Is Required for Long-Term Depression and AMPA Receptor Internalization. Cell *141*, 859–871. https://doi.org/10.1016/j.cell.2010.03.053.

Lim, J., Ryu, J., Kang, S., Noh, H.J., and Kim, C.H. (2019). Autism-like behaviors in male mice with a Pcdh19 deletion. Mol. Brain *12*, 95. https://doi.org/10.1186/s13041-019-0519-3.

Lois, C., Hong, E.J., Pease, S., Brown, E.J., and Baltimore, D. (2002). Germline transmission and tissue-specific expression of transgenes delivered by lentiviral vectors. Science *295*, 868–872. https://doi.org/10.1126/science.1067081.

Longaretti, A., Forastieri, C., Toffolo, E., Caffino, L., Locarno, A., Misevičiūtė, I., Marchesi, E., Battistin, M., Ponzoni, L., Madaschi, L., et al. (2020). LSD1 is an environmental stress-sensitive negative modulator of the glutamatergic synapse. Neurobiol. Stress *13*, 100280. https://doi.org/10.1016/j.ynstr.2020.100280.

van Loo, K.M.J., Rummel, C.K., Pitsch, J., Müller, J.A., Bikbaev, A.F., Martinez-Chavez, E., Blaess, S., Dietrich, D., Heine, M., Becker, A.J., and Schoch, S. (2019). Calcium channel subunit α2δ4 is regulated by early growth response 1 and facilitates epileptogenesis. J. Neurosci. *39*, 3175–3187. https://doi.org/10.1523/jneurosci.1731-18.2019.

Marambaud, P., Wen, P.H., Dutt, A., Shioi, J., Takashima, A., Siman, R., and Robakis, N.K. (2003). A CBP binding transcriptional repressor produced by the PS1/epsilon-cleavage of N-cadherin is inhibited by PS1 FAD mutations. Cell *114*, 635–645. https://doi.org/10.1016/j.cell.2003.08.008.

Marcello, E., Gardoni, F., Mauceri, D., Romorini, S., Jeromin, A., Epis, R., Borroni, B., Cattabeni, F., Sala, C., Padovani, A., and Di Luca, M. (2007). Synapse-associated protein-97 mediates alpha-secretase ADAM10 trafficking and promotes its activity. J. Neurosci. *27*, 1682–1691. https://doi.org/10.1523/jneurosci.3439-06.2007.

McCusker, C.D., and Alfandari, D. (2009). Life after proteolysis: exploring the signaling capabilities of classical cadherin cleavage fragments. Commun. Integr. Biol. *2*, 155–157. https://doi.org/10.4161/cib.7700.

Moretto, E., Longatti, A., Murru, L., Chamma, I., Sessa, A., Zapata, J., Hosy, E., Sainlos, M., Saint-Pol, J., Rubinstein, E., et al. (2019). TSPAN5 enriched microdomains provide a platform for dendritic spine maturation through neuroligin-1 clustering. Cell Rep *29*, 1130–1146.e8, e8. https://doi.org/10.1016/j.celrep.2019.09.051.

Nagappan-Chettiar, S., Johnson-Venkatesh, E.M., and Umemori, H. (2017). Activity-dependent proteolytic cleavage of cell adhesion molecules regulates excitatory synaptic development and function. Neurosci. Res. *116*, 60–69. https://doi.org/10.1016/j.neures.2016.12.003.

Niazi, R., Fanning, E.A., Depienne, C., Sarmady, M., and Abou Tayoun, A.N. (2019). A mutation update for the PCDH19 gene causing early-onset epilepsy in females with an unusual expression pattern. Hum. Mutat. *40*, 243–257. https://doi.org/10.1002/humu.23701.

Pederick, D.T., Richards, K.L., Piltz, S.G., Kumar, R., Mincheva-Tasheva, S., Mandelstam, S.A., Dale, R.C., Scheffer, I.E., Gecz, J., Petrou, S., et al. (2018). Abnormal cell sorting underlies the unique X-linked inheritance of PCDH19 epilepsy. Neuron *97*, 59–66.e5, e5. https://doi.org/10.1016/j.neuron.2017.12.005.

Pelucchi, S., Vandermeulen, L., Pizzamiglio, L., Aksan, B., Yan, J., Konietzny, A., Bonomi, E., Borroni, B., Padovani, A., Rust, M.B., et al. (2020). Cyclase-associated protein 2 dimerization regulates cofilin in synaptic plasticity and

Alzheimer's disease. Brain Commun. *2*, fcaa086. https://doi.org/10.1093/braincomms/fcaa086.

Pillai-Nair, N., Panicker, A.K., Rodriguiz, R.M., Gilmore, K.L., Demyanenko, G.P., Huang, J.Z., Wetsel, W.C., and Maness, P.F. (2005). Neural cell adhesion molecule-secreting transgenic mice display abnormalities in GABAergic interneurons and alterations in behavior. J. Neurosci. *25*, 4659–4671. https://doi.org/10.1523/jneurosci.0565-05.2005.

Pilotto, S., Speranzini, V., Marabelli, C., Rusconi, F., Toffolo, E., Grillo, B., Battaglioli, E., and Mattevi, A. (2016). LSD1/KDM1A mutations associated to a newly described form of intellectual disability impair demethylase activity and binding to transcription factors. Hum. Mol. Genet. *25*, ddw120–2587. https://doi.org/10.1093/hmg/ddw120.

Rakotomamonjy, J., Sabetfakhri, N.P., McDermott, S.L., and Guemez-Gamboa, A. (2020). Characterization of seizure susceptibility in Pcdh19 mice. Epilepsia *61*, 2313–2320.

Reiss, K., Maretzky, T., Haas, I.G., Schulte, M., Ludwig, A., Frank, M., and Saftig, P. (2006). Regulated ADAM10-dependent ectodomain shedding of gamma-protocadherin C3 modulates cell-cell adhesion. J. Biol. Chem. *281*, 21735–21744. https://doi.org/10.1074/jbc.m602663200.

Robens, B.K., Yang, X., McGraw, C.M., Turner, L.H., Robens, C., Thyme, S., Rotenberg, A., and Poduri, A. (2021). Mosaic and non-mosaic pcdh19 mutation leads to neuronal hyperexcitability in zebrafish. Preprint at BioRxiv. https://doi.org/10.1101/2021.09.03.458732.

Rusconi, F., Mancinelli, E., Colombo, G., Cardani, R., Da Riva, L., Bongarzone, I., Meola, G., and Zippel, R. (2010). Proteome profile in Myotonic Dystrophy type 2 myotubes reveals dysfunction in protein processing and mitochondrial pathways. Neurobiol. Dis. *38*, 273–280. https://doi.org/10.1016/j.nbd.2010.01.017.

Rusconi, F., Paganini, L., Braida, D., Ponzoni, L., Toffolo, E., Maroli, A., Landsberger, N., Bedogni, F., Turco, E., Pattini, L., et al. (2015). LSD1 neurospecific alternative splicing controls neuronal excitability in mouse models of epilepsy. Cereb. Cortex *25*, 2729–2740. https://doi.org/10.1093/cercor/bhu070.

Rusconi, F., Grillo, B., Ponzoni, L., Bassani, S., Toffolo, E., Paganini, L., Mallei, A., Braida, D., Passafaro, M., Popoli, M., et al. (2016). LSD1 modulates stress-evoked transcription of immediate early genes and emotional behavior. Proc. Natl. Acad. Sci. U S A *113*, 3651–3656. https://doi.org/10.1073/pnas.1511974113.

Rusconi, F., Grillo, B., Toffolo, E., Mattevi, A., and Battaglioli, E. (2017). NeuroLSD1: splicing-generated epigenetic enhancer of neuroplasticity. Trends Neurosci. *40*, 28–38. https://doi.org/10.1016/j.tins.2016.11.002.

Schedin-Weiss, S., Caesar, I., Winblad, B., Blom, H., and Tjernberg, L.O. (2016). Super-resolution microscopy reveals γ-secretase at both sides of the neuronal synapse. Acta Neuropathol. Commun. *4*, 29. https://doi.org/10.1186/s40478-016-0296-5.

Schindelin, J., Arganda-Carreras, I., Frise, E., Kaynig, V., Longair, M., Pietzsch, T., Preibisch, S., Rueden, C., Saalfeld, S., Schmid, B., et al. (2012). Fiji: an open-source platform for biological-image analysis. Nat. Methods *9*, 676–682. https://doi.org/10.1038/nmeth.2019.

Serratto, G.M., Pizzi, E., Murru, L., Mazzoleni, S., Pelucchi, S., Marcello, E., Mazzanti, M., Passafaro, M., and Bassani, S. (2020). The epilepsy-related protein PCDH19 regulates tonic inhibition, GABAAR kinetics, and the intrinsic excitability of hippocampal neurons. Mol. Neurobiol. *57*, 5336–5351. https://doi.org/10.1007/s12035-020-02099-7.

Shan, W., Nagai, T., Tanaka, M., Itoh, N., Furukawa-Hibi, Y., Nabeshima, T., Sokabe, M., and Yamada, K. (2018). Neuronal PAS domain protein 4 (Npas4) controls neuronal homeostasis in pentylenetetrazole-induced epilepsy through the induction of Homer1a. J. Neurochem. *145*, 19–33. https://doi.org/10.1111/jnc.14274.

Smolders, I., Khan, G.M., Manil, J., Ebinger, G., and Michotte, Y. (1997). NMDA receptor-mediated pilocarpine-induced seizures: characterization in freely moving rats by microdialysis. Br. J. Pharmacol. *121*, 1171–1179. https://doi.org/10.1038/sj.bjp.0701231.

Sogorb-Esteve, A., García-Ayllón, M.S., Llansola, M., Felipo, V., Blennow, K., and Sáez-Valero, J. (2018). Inhibition of γ-secretase leads to an increase in presenilin-1. Mol. Neurobiol. *55*, 5047–5058. https://doi.org/10.1007/s12035-017-0705-1.

Suzuki, K., Hayashi, Y., Nakahara, S., Kumazaki, H., Prox, J., Horiuchi, K., Zeng, M., Tanimura, S., Nishiyama, Y., Osawa, S., et al. (2012). Activity-dependent proteolytic cleavage of neuroligin-1. Neuron *76*, 410–422. https://doi.org/10.1016/j.neuron.2012.10.003.

Toffolo, E., Rusconi, F., Paganini, L., Tortorici, M., Pilotto, S., Heise, C., Verpelli, C., Tedeschi, G., Maffioli, E., Sala, C., et al. (2014). Phosphorylation of neuronal Lysine-Specific Demethylase 1LSD1/KDM1A impairs transcriptional repression by regulating interaction with CoREST and histone deacetylases HDAC1/2. J. Neurochem. *128*, 603–616. https://doi.org/10.1111/jnc.12457.

Vlaskamp, D.R.M., Bassett, A.S., Sullivan, J.E., Robblee, J., Sadleir, L.G., Scheffer, I.E., and Andrade, D.M. (2019). Schizophrenia is a later-onset feature of PCDH19 girls clustering epilepsy. Epilepsia *60*, 429–440. https://doi.org/10.1111/epi.14678.

Wang, J., Telese, F., Tan, Y., Li, W., Jin, C., He, X., Basnet, H., Ma, Q., Merkurjev, D., Zhu, X., et al. (2015). LSD1n is an H4K20 demethylase regulating memory formation via transcriptional elongation control. Nat. Neurosci. *18*, 1256–1264. https://doi.org/10.1038/nn.4069.

Wolfe, M.S., and Kopan, R. (2004). Intramembrane proteolysis: theme and variations. Science *305*, 1119–1123. https://doi.org/10.1126/science.1096187.

Yuanxiang, P., Bera, S., Karpova, A., Kreutz, M.R., and Mikhaylova, M. (2014). Isolation of CA1 nuclear enriched fractions from hippocampal slices to study activity-dependent nuclear import of synapto-nuclear messenger proteins. J. Vis. Exp *10*, e51310. https://doi.org/10.3791/51310.

Zhang, Y., Pak, C., Han, Y., Ahlenius, H., Zhang, Z., Chanda, S., Marro, S., Patzke, C., Acuna, C., Covy, J., et al. (2013). Rapid single-step induction of functional neurons from human pluripotent stem cells. Neuron *78*, 785–798. https://doi.org/10.1016/j.neuron.2013.05.029.

Zhang, Y., Chen, G., Gao, B., Li, Y., Liang, S., Wang, X., and Zhu, B. (2016). NR4A1 knockdown suppresses seizure activity by regulating surface expression of NR2B. Sci. Rep. *6*, 37713. https://doi.org/10.1038/srep37713.

Zibetti, C., Adamo, A., Binda, C., Forneris, F., Toffolo, E., Verpelli, C., Ginelli, E., Mattevi, A., Sala, C., and Battaglioli, E. (2010). Alternative splicing of the histone demethylase LSD1/KDM1 contributes to the modulation of neurite morphogenesis in the mammalian nervous system. J. Neurosci. *30*, 2521–2532. https://doi.org/10.1523/jneurosci.5500-09.2010.

STAR★METHODS

KEY RESOURCE TABLE

REAGENT or RESOURCE	SOURCE	IDENTIFIER
Antibodies		
Rabbit polyclonal anti-PCDH19	Bethyl Laboratories	Cat# A304-468A; RRID: AB_2620662
Mouse monoclonal anti-PCDH19	Abcam	Cat# ab57510; RRID: AB_944674
Guinea Pig polyclonal anti-MAP2	Synaptic System	Cat# 188 004; RRID: AB_2138181
Rabbit polyclonal anti-V5 epitope tag	Millipore	Cat# AB3792; RRID: AB_91591
Rabbit polyclonal anti-HA (Y-11)	Santa Cruz Biotechnology	Cat# sc-805; RRID: AB_631618
Rabbit polyclonal anti-HA	Thermo Fisher Scientific	Cat# 71-5500; RRID: AB_2533988
Rabbit polyclonal anti-NR4A1	Proteintech	Cat# 12235-1-AP; RRID: AB_10644125
Rabbit polyclonal anti-PS1	Proteintech	Cat# 16163-1-AP; RRID: AB_2237805
Rabbit monoclonal anti-GAPDH (clone 14C10)	Cell Signaling Technology	Cat# 2118, RRID: AB_561053
Rabbit polyclonal anti-GAPDH	Santa Cruz Biotechnology	Cat# sc-25778; RRID: AB_10167668
Mouse monoclonal anti-alpha tubulin (clone B-5-1-2)	Sigma-Aldrich	Cat# T5168; RRID: AB_477579
Rabbit polyclonal anti-LSD1	Abcam	Cat# ab17721; RRID: AB_443964
Rabbit polyclonal anti-CoREST	Millipore	Cat# 07-455; RRID: AB_310629
Rabbit monoclonal anti-SRF (D71A9)	Cell Signaling Technology	Cat# 5147; RRID: AB_10694554
Rabbit polyclonal anti-HDAC2	Abcam	Cat# ab7029, RRID:AB_305706
Rabbit polyclonal anti-NOVA1	Sigma-Aldrich	Cat# HPA004155; RRID: AB_1079497
Mouse monoclonal anti-Transferrin Receptor (clone H68.4)	Thermo Fisher Scientific	Cat# 13-6800; RRID: AB_2533029
Mouse monoclonal anti-PSD95	NeuroMab	Cat# 73-28; RRID: AB_10698024
Rabbit polyclonal anti-PCDH9	Abcam	Cat# ab171166
Goat polyclonal anti-rabbit IgG (H + L) Alexa Fluor 488	Thermo Fisher Scientific	Cat# A-11034; RRID: AB_2576217
Goat polyclonal anti-rabbit IgG (H + L) Alexa Fluor 555	Thermo Fisher Scientific	Cat# A-21429; RRID: AB_2535850
Goat polyclonal anti-mouse IgG (H + L) Alexa Fluor 488	Thermo Fisher Scientific	Cat# A-11029; RRID: AB_138404
Donkey polyclonal anti-Guinea Pig IgG (H + L) Alexa Fluor 647	Jackson ImmunoResearch Labs	Cat# 706-605-148; RRID:AB_2340476
Goat polyclonal anti-rabbit IgG (H + L) conjugate Horseradish Peroxidase	Jackson ImmunoResearch Labs	Cat# 111-035-00; RRID: AB_2313567
Goat polyclonal anti-mouse IgG (H + L) conjugate Horseradish Peroxidase	GE Healthcare	Cat# NA931, RRID: AB_772210
Goat polyclonal anti-Rabbit IgG (H + L) conjugate Horseradish Peroxidase	Invitrogen	Cat# 31460; RRID: AB_228341
Bacterial and Virus strains		
Escherichia Coli BL21	Invitrogen	N/A
Chemicals, peptides, and recombinant proteins		
N-Methyl-D-aspartic acid (NMDA)	Sigma-Aldrich	Cat# M3262
D(−)-2-Amino-5-phosphonopentanoic acid (APV)	Merck	Cat# A8054
(RS)-α-Amino-3-hydroxy-5-methyl-4-isoxazolepropionic acid (AMPA)	Tocris	Cat# 0169
Bicuculline	Sigma-Aldrich	Cat# 14340
Tetrodotoxin	Tocris	Cat# 1078
(+)-MK-801 hydrogen maleate (MK-801)	Merck	Cat# M107
(2*S*)-*N*-[(3,5-Difluorophenyl)acetyl]-L-alanyl-2-phenyl]glycine 1,1-dimethylethyl ester (DAPT)	Tocris	Cat# 2634

(*Continued on next page*)

Continued

REAGENT or RESOURCE	SOURCE	IDENTIFIER
(2*R*)-N[4]-Hydroxy-N[1]-[(1*S*)-1-(1*H*-indol-3-ylmethyl)-2-(methylamino)-2-oxoethyl]-2-(2-methylpropyl)butanediamide (GM6001)	Tocris	Cat# 2983
GI4023, (2R)-N-[(1S)-2,2-Dimethyl-1-[(methylamino)carbonyl]-propyl]-2-[(1S)-1-[formyl(hydroxy)amino]ethyl]-5-phenylpentanamide (GI254023X)	Sigma-Aldrich	Cat# SML0789
MG-132	Sigma-Aldrich	Cat# SML1135
(5*S*)-(*tert*-Butoxycarbonylamino)-6-phenyl-(4*R*)-hydroxy-(2*R*)-benzylhexanoyl)-L-leucy-L-phenylalaninamide (L-685, 458)	MedChemExpress	Cat# HY-19369
Caspase Inhibitor I	Calbiochem	Cat# 187389-52-2
EZ-Link[TM] Sulfo-NHS-SS-Biotin	Thermo Fisher Scientific	Cat# 21331
4′,6-Diamidino-2-Phenylindole, Dihydrochloride (DAPI)	Invitrogen	Cat# D1306
Experimental models: Cell lines		
Human: HEK293 FT	Thermo Fisher Scientific	Cat# R70007
Human: iPSC WTC-1	Dr. Nael Nadif Kasri lab, cells obtained from Coriell institute	Cat# GM25256
Experimental models: Organisms/strains		
Rat: Wistar Rats	Charles River Laboratories	N/A
Mouse: C57BL/6N	Charles River Laboratories	N/A
Oligonucleotides		
See Table S9	Sigma-Aldrich	N/A
Recombinant DNA		
PCDH19 shRNA in pLVTHM, rat specific	In House, Bassani et al., 2018	N/A
PCDH19 shRNA in pLVTHM, human specific	This Paper	N/A
PCDH19 control shRNA (scramble) in pLVTHM	In House, Bassani et al., 2018	N/A
PCDH9 in pIRES2	In House, Bassani et al., 2018	N/A
pGEX 4T1	In House	N/A
GST-CTF in pGEX 4T1	In House, Bassani et al., 2018	N/A
cFUW	In House	N/A
PCDH19-V5 in cFUW	In House, Bassani et al., 2018	N/A
CTF-V5 in cFUW	In House, Bassani et al., 2018	N/A
CTF-V5 NLS_{br1BR2} in cFUW	This paper	N/A
PCDH19-879Δ in cFUW-RFP	In House, Bassani et al., 2018	N/A
pCMV-HA	Clontech	Cat# 635690
CTF in pCMV-HA (HA-CTF)	In House	N/A
CTFa in pCMV-HA (HA-CTFa)	In House	N/A
CTFb in pCMV-HA (HA-CTFb)	In House	N/A
pIRES2-EGFP	Clontech	Cat# V011106
LSD1 in pCGN-HA (HA-LSD1)	Dr. E. Battaglioli, Toffolo et al., 2014	N/A
nLSD1 in pCGN-HA (HA-nLSD1)	Dr. E. Battaglioli, Toffolo et al., 2014	N/A
NOVA1 in pCGN-HA (HA-NOVA1)	Dr. E. Battaglioli, Rusconi et al., 2015	N/A
Software and algorithms		
Image Lab 6.0	Bio - Rad	N/A
Fiji	ImageJ	RRID: SCR_002285; http://fiji.sc
Prism Software	GraphPad Prism	RRID: SCR_002798, http://www.graphpad.com/
cNLS Mapper	Kosugi et al., 2009	http://nls-mapper.iab.keio.ac.jp/cgi-bin/NLS_Mapper_form.cgi

RESOURCE AVAILABILITY

Lead contact

Further information and requests for resources and reagents should be directed to and will be fulfilled by le Lead Contact, Silvia Bassani (silvia.bassani@in.cnr.it).

Materials availability

Plasmids generated in this study are available from the Lead contact with a completed Materials Transfer Agreement.

Data and code availability

- All data reported in this paper will be shared by the Lead contact upon request.
- This paper does not report original code.
- Any additional information required to reanalyze the data reported in this paper is available from the Lead contact upon request.

EXPERIMENTAL MODEL AND SUBJECT DETAILS

Primary hippocampal neurons

Dissociated primary hippocampal neurons were obtained from Sprague Dawley rat embryos of either sex at embryonic day 18 (E18). Pregnant rats (2-3 months of age) were purchased from Charles River, Italy. Animal care and sacrifice were performed in accordance with the CNR licensing released by the Italian Ministry of Health (authorization no. 100/2016 and 2D46A.N.463). After trypsin-mediated and mechanical disaggregation of hippocampal tissue, the dissociated neurons were plated on poly-D-lysine coated coverslips at a density of 75.000/well (for ICC experiments) or 150.000/well (for biochemical assays). Neurons were grown in Neurobasal Medium (Life Technologies) supplemented with homemade B-27, 0.25% L-glutamine, 1% penicillin/streptomycin and 0.125% Glutamate (Sigma-Aldrich) and maintained at 37°C, 5% CO2. The B-27 was prepared as previously described (Serratto et al., 2020). At days *in vitro* (DIV) 4 the 50% of the medium was replaced by fresh medium without glutamate. Primary hippocampal neurons were either transfected with calcium phosphate method (ICC experiments) at DIV 4, or transduced with defective lentiviral particles (biochemical assays) at DIV 8, and used at DIV 12. Brain regions from pregnant rats (2-3 months of age) were used for crude synaptosomes preparation (hippocampus) and GST pull-down assay (hippocampus and cortex) in order to reduce the number of animals.

C57BL/6N mice

Adult (6-7 weeks old) male mice (C57BL/6N, wild type) were purchased from Charles River (Italy). All animal care, experimental procedure and sacrifice were performed in accordance with the CNR licensing released by the Italian Ministry of Health (authorization no. 670/2018-PR). After shipping, mice were group-housed in a non-specific pathogen free (SPF) animal facility under standard conditions for few days. Age and sex-matched C57BL/6N mice were randomly assigned to experimental groups (controls or pilocarpine treated). Pilocarpine-treated mice (single dose, 270 mg/kg i.p., (Rusconi et al., 2015)) were sacrificed at the occurrence of the first tonic-clonic seizure and their brain isolated. Coronal brain slices (400 μm thick) containing the hippocampus from untreated and treated mice were cut with a vibrating blade microtome (Leica Biosystems, Italy), incubated 2.5 h in oxygenated artificial cerebrospinal fluid (aCSF, mM: 125 NaCl, 2.5 KCl, 2 CaCl2, 1 MgCl2, 26 NaHCO3, 1.25 NaH_2PO_4, 25 glucose, pH 7.3–7.4; 95% O_2 and 5% CO_2) and fractionated as described below.

HEK cells

HEK 293FT cells (sex: female, Thermo Fisher Scientific) were cultured in DMEM with high glucose and pyruvate (Thermo Fisher Scientific) supplemented with 10% fetal bovine serum (FBS) (GIBCO), 1% L-glutamine and 0.1% gentamycin (Thermo Fisher Scientific). HEK 293FT cells at 50%–60% confluence were transiently transfected by using the Jet-PEI Transfection Kit (Polyplus Transfection) according to the manufacturer's instructions. After 24-48 hours, cells were fixed for ICC or lysed for biochemical experiments. Replication-incompetent lentiviral vectors to deliver PCDH19 shRNAs, control shRNA and CTF-V5 were produced in HEK 293FT cells as previously described (Lois et al., 2002). Cells were not authenticated before use. All cells were maintained at 37°C, 5% CO2.

Human-induced pluripotent stem cells

Human-induced pluripotent stem cells (hiPSCs) used in this study were obtained from reprogrammed control skin fibroblasts of clinically normal adult male subject (30 years old). hiPSCs were cultured on Matrigel (Corning, # 356237) in E8 Flex (Thermo Fisher Scientific) supplemented with primocin (0.1 μg/mL, Invitrogen), puromycin (0.5 μg/mL) and G418 (50 μg/mL) at 37°C/5% CO_2. Medium was refreshed every 2-3 days and cells were passaged twice per week using an enzyme-free reagent (ReLeSR, Stem Cell Technologies).

hiPSCs were differentiated into neurons using the Ngn2-protocol as previously described (Frega et al., 2017; Zhang et al., 2013). Briefly, hiPSCs were directly differentiated into excitatory cortical layer 2/3 neurons by overexpressing Neurogenin 2 (Ngn2) upon doxycycline treatment. Neuronal maturation was supported by rat astrocytes, which were added to the culture in a 1:1 ratio two

days after hiPSCs plating. At DIV 3 the medium was changed to Neurobasal medium (Thermo Fisher Scientific, # 21103049) supplemented with B-27 (Thermo Fisher Scientific, # 17504001), glutaMAX (Thermo Fisher Scientific, # 35050061), primocin (0.1 μg/mL), NT3 (10 ng/mL) (Thermo Fisher Scientific, # PHC7036), BDNF (10 ng/mL), and doxycycline (4 μg/mL). Cytosine β-D-arabinofuranoside (2 μM) (Sigma-Aldrich, #C1768) was added to remove any proliferating cell from the culture at DIV 3. From DIV 6 onwards half of the medium was refreshed every other day. From DIV 10 onwards the medium was additionally supplemented with 2.5% FBS to support astrocyte viability. Neuronal cultures were kept through the whole differentiation process at 37°C/5% CO2.

METHOD DETAILS

Plasmids

PCDH19-V5 and CTF-V5 in cFUW, PCDH19-879Δ in cFUW-RFP, GST-CTF in pGEX 4T1, PCDH9 in pIRES2, PCDH19 shRNA (rat-specific target sequence 5′-gagcagcatgaccaatacaat-3′) and control shRNA (scramble, target sequence 5′-gctgagcgaaggagagat-3′) in pLVTHM vector were previously described in (Bassani et al., 2018).

The sequence of CTF-V5 in cFUW was mutated in correspondence of the first of the two basic regions (BR1 and BR2) that compose PCDH19 CTF putative NLS (CTF-V5 NLS_{BR1BR2}, amino acids 760-782, RGKRIAEYSYGHQKKSSKKKKIS). Amino acids 760, 762 and 763 were replaced by alanines by using the Agilent Technologies QuickChange II XL Site-Directed Mutagenesis Kit (Agilent, USA) to obtain a mutant CTF-V5 (CTF-V5 NLS_{br1BR2}, *A*G*AA*IAEYSYGHQKKSSKKKKIS, mutant amino acids in italics). The primers in Table S9 were used for mutagenesis.

PCDH19 shRNA target sequence was modified for use in hiPSCs-derived neurons (human-specific target sequence 5′-GAGCAG CACGACCAATACAAC-3′) and cloned in pLVTHM vector (Addgene). PCDH19 CTF and its proximal and distal parts (CTFa, aa 700-890 and CTFb, aa 891-1148) were subcloned in pCMV-HA (Clontech) to obtain HA-CTF, HA-CTFa, and HA-CTFb. HA-LSD1 and HA-nLSD1 in pCGN were previously described in (Toffolo et al., 2014). NOVA1 was subcloned in pCGN-HA vector (Rusconi et al., 2015). pIRES2 EGFP plasmid was from Clontech.

NLS prediction

To predict PCDH19 NLS, the freely available program cNLS Mapper (Kosugi et al., 2009) was used. cNLS Mapper predicted the following bipartite NLS (prediction cut off 7) "RGKRIAEYSYGHQKKSSKKKKIS" (amino acids 760-782; UniProtKB: Q80TF3, mouse PCDH19; UniprotKB: A0A0G2K8I5, rat PCDH19; UniprotKB: Q8TAB3-1, human PCDH19).

Treatments on primary neurons

Primary hippocampal neurons were treated at DIV 12 or DIV 22 (DIV 22, Figures S1A and S1B) with the following reagents: NMDA (50 μM for 3, 6, 10, 20 or 30 min; Sigma-Aldrich), NMDA (50 μM, 30 min) plus APV (100 μM, 30 min; Merck), AMPA (100 μM, 30 min; Tocris), bicuculline (BIC, 40 μM, 30 min; Sigma-Aldrich), tetrodotoxin (TTX, 2 μM, 30 min; Tocris).

In particular, NMDA was applied for 6 min for experiments in Figures 3C, 3D, 3E, 6C, S3A–S3D, S5A, and S5B; for 20 min for experiments in Figures 2B, 3A, and 3B; for 30 min for Figures 1A–1D, 2A, 2B, 2D, 4E, 7A–7D, S1A–S1D, and S2B; for 3-30 min for experiments in Figure 2C.

The protocol to activate synaptic or extrasynaptic NMDARs was previously reported (Karpova et al., 2013). Briefly, neurons were treated with BIC (50 μM) and 4-aminopyridine (4-AP, 2.5 mM; Merck) for 30 min, in order to promote presynaptic glutamate release and activate specifically postsynaptic NMDARs. Neurons were treated with the same concentration of BIC and 4-AP plus the blocker of open NMDA channels MK-801 (10 μM, Merck) for 30 min to irreversibly block activated synaptic NMDA receptors. Thereafter, neurons were washed to remove unbound MK-801 and NMDA (100 μM, 3 min) was applied to activate extrasynaptic NMDARs. Neurons were washed again and incubated for 30 min before ICC.

DAPT (10 μM, Tocris), GM6001 (10 μM, Tocris), GI254023X (5μM, Sigma-Aldrich), MG132 (10μM, Sigma-Aldrich), L-685, 458 (10 μM, MedChemExpress) were added to the neuron media 20 min, 4 h or 24 h before NMDA treatment (50 μM, 20 min or 6 min), as indicated.

The pancaspase inhibitor (caspase inhibitor I, 20 μM, Calbiochem) was added to the neuron media 4 h before NMDA treatment (50 μM, 6 min).

ICC, image acquisition and analysis

Cultured hippocampal neurons at DIV 12 or 22 (DIV 22, Figures S1A and S1B) and HEK 293FT cells were fixed with 4% paraformaldehyde/4% sucrose for 10 min at room temperature (RT). Cells were incubated with primary (2 h) and secondary (1 h) antibodies in gelatin detergent buffer (GDB: 30 mM phosphate buffer at pH 7.4 containing 0.2% gelatin, 0.5% Triton X-100 and 0.8 M NaCl). The following primary antibodies were used: anti-PCDH19 (1:5000, Bethyl); anti-MAP2 (1:2000, Synaptic System); anti-V5 (1:400, Millipore); anti-HA (1:200, Santa Cruz Biotechnology); anti-NR4A1 (1:400, Proteintech). Secondary antibodies conjugated to the following dyes were used: Alexa Fluor 488 (1:400, Invitrogen), Alexa Fluor 555 (1:400, Invitrogen) and DyLight 649 (1:400, Jackson Laboratories). Nuclei were stained by incubating cells with DAPI (Invitrogen; 1:10000 in PBS buffer) for 5 min at RT. Images were acquired with Zeiss LSM 800 confocal microscope (Carl Zeiss, Italy) by using 40X/1.3 and 63X/1.4 oil objectives. Images were obtained from the z-projection (maximum intensity) of 6-12 stacks taken at 0.75 μm intervals at 1024 × 1024 pixel resolution. Confocal images were

analyzed with Fiji (Schindelin et al., 2012). DAPI, MAP2 or GFP channels were used to draw regions of interest (ROIs) corresponding to the nuclear and dendritic compartments or to entire neurons. The mean fluorescence intensity along dendrites and in the soma was quantified on z-stack projections. Nuclear fluorescent signals (mean intensity) were quantified on a single stack in which the nuclear section was wider, to exclude the signal coming from perinuclear regions.

hiPSCs-derived neurons at DIV 21 were stimulated with NMDA, fixed, and stained with DAPI and anti-PCDH19 (1:30000, Bethyl Laboratories). Neurons were imaged at 63X using the Zeiss Axio Imager Z1 equipped with apotome. All conditions within a batch were acquired with the same settings in order to compare signal intensities between different experimental conditions. Fluorescent signals were quantified using Fiji (Schindelin et al., 2012) software.

Biotinylation assay

To selectively label surface proteins, cultured hippocampal neurons at DIV 12 were treated with membrane-impermeable sulfo-NHS-SS-biotin (0.3 mg/mL, EZ-Link sulfo-NHS-LC-Biotin; Thermo Scientific) for 5 min at 37°C as previously described in (Bassani et al., 2018). Input, biotin-labeled surface proteins and non-biotinylated proteins were subjected to SDS-PAGE.

Crude synaptosomes and TIF

Crude synaptosomes were obtained as described in (Moretto et al., 2019). Briefly, hippocampi and cortices were homogenized with glass-teflon potter in homogenization buffer (0.32 M sucrose, 10 mM HEPES-NaOH, protease inhibitors, pH 7.4). Total homogenate (H) was centrifuged (1000 g, 10 min, 4°C) to obtain the pellet (P1), corresponding to the nuclear fraction, and the supernatant (S1). S1 fraction was centrifuged (10,000 g, 15 min, 4°C) to obtain the pellet (P2), corresponding to crude synaptosomes fraction, and the supernatant (S2), containing cytosolic components and light membranes. The P2 fraction was resuspended in homogenization buffer and centrifuged (10,000 g, 15 min, 4°C) to wash the synaptosomes. The fractions were resuspended in homogenization buffer and sample buffer was added before SDS-PAGE and western blotting.

The triton-insoluble fraction (TIF) from hippocampal cultures was obtained as described in (Pelucchi et al., 2020). Briefly, neurons were lysed at 4°C using a glass–glass homogenizer in ice-cold buffer with cOmplete^{TM} Protease Inhibitor Cocktail (Roche), Ser/Thr and Tyr phosphatase inhibitors (Sigma-Aldrich), 0.32 M Sucrose, 1 mM Hepes, 1 mM NaF, 0.1 mM PMSF, 1 mM MgCl2. Homogenates were centrifuged at 13,000 g for 15 min at 4°C. Triton X- extraction of the resulting pellet was carried out at 4°C for 15 min in an extraction buffer (0.5% Triton X-, 75 mM KCl and protease inhibitors, Roche). After extraction, the samples were centrifuged at 100,000g for 1 h at 4°C and the TIFs obtained were resuspended in 20 mM HEPES with protease inhibitors (Roche).

Subcellular fractionation

Primary hippocampal neurons were fractionated as previously described (Rusconi et al., 2010). Briefly, neurons were lysed in hypotonic buffer (10 mM Hepes pH 7.5, 0.5% NP-40, 10 mM KCl, 1mM EDTA, 1mM DTT, protease and phosphatase inhibitors) and centrifuged at 400 g for 10 min at 4°C to isolate nuclei fraction (P1). The supernatant was further centrifuged (15,000 g, 10 min, 4°C) to obtain the cytosol and membrane enriched fraction (S1). The nuclei fraction (P1) was re-suspended in hypertonic buffer (20 mM Tris-HCl pH 7.5, 0.6 M NaCl, 0.2 mM EDTA, 1.4 mM MgCl2, 25% glycerol, protease and phosphatase inhibitors) and centrifuged at 80,000 g for 10 min at 4°C to separate nuclear aggregates (P2) from the soluble nuclear fraction (S2). Fractions, including an aliquot of total homogenate (H), were re-suspended in SDS sample buffer, boiled for 5 min and subjected to SDS-PAGE.

Mouse coronal brain slices were fractionated as described in (Yuanxiang et al., 2014). Briefly, slices were homogenized in hypotonic lysis buffer (mM: 10 HEPES, 1.5 MgCl2, 10 KCl, pH 7.9, protease and phosphatase inhibitors)
to obtain the homogenate fraction (H). Lysates were centrifuged at 11,000 rpm for 1 min to separate the supernatant (cytosolic fraction, C), from the nuclei-membranes enriched fraction (N-M), which was suspended in hypotonic buffer. Fractions were re-suspended in SDS sample buffer, boiled for 5 min and subjected to SDS-PAGE.

GST pull-down

GST-tagged proteins were produced in *E.coli* strain BL21 and purified according to standard procedures. Homogenates in modified RIPA buffer (50 mM Tris-HCl, 150 mM NaCl, 1 mM EDTA, 1% NP40, 1% Triton X-100, pH 7.4, protease inhibitor cocktail) from adult rat cortex and hippocampus were incubated overnight at 4°C with either GST-CTF or GST immobilized on beads. Beads and associated protein complexes were washed 5 times with lysis buffer and resuspended in SDS sample buffer, boiled for 5 min and subjected to SDS-PAGE and immunoblotting.

CoIP

HEK 293FT cells were transiently transfected using a JetPEI Transfection Kit (Polyplus Transfection) according to manufacturer's instructions and lysed 48 h later in modified RIPA buffer. After incubation on a rotating wheel for 1 h at 4°C, homogenates were cleared by centrifugation (16,000 g, 10 min, 4°C). The supernatant was incubated overnight at 4°C on a rotating wheel with the appropriate rabbit antibodies (anti-V5, Millipore; anti-HA, Thermo Fisher Scientific; non-immune control IgG, Sigma-Aldrich). Protein A-agarose beads (Amersham GE Healthcare) were added and, after incubation on a rotating wheel for 2 h at 4°C, beads and coimmunoprecipitated complexes were collected by centrifugation and washed 5 times with modified RIPA buffer. After resuspension in SB3X, protein complexes were analyzed by SDS-PAGE.

Immunoblotting

Proteins from cell lysates and brain tissue homogenates were separated by SDS-PAGE and transferred to 0.22 μm pore-size nitrocellulose membranes (Amersham GE Healthcare) according to standard procedures. Membranes were blocked for 1 h at RT with 5% non-fat milk in TBST (10 mM Tris-HCl, 150 mM NaCl, 0.1% Tween-20, pH 8.0). Membranes were incubated with primary antibodies in TBST overnight (ON) at 4°C and with secondary antibodies in TBST 1 h at RT. After each incubation period, membranes were washed three times for 10 min with TBST. Immunoreactive bands were visualized by enhanced chemiluminescence (ECL, PerkinElmer). Signals were quantified by using Fiji (Schindelin et al., 2012) (scanned images from X-ray films) or ImageLab software (images acquired with Chemidoc). The following primary antibodies were used: anti-PCDH19 (1:20,000, Bethyl Laboratories, Inc.); anti-PCDH19 (1:500, AbCam; Figure S2), anti-GAPDH (1:2,000, Santa Cruz); anti-LSD1 (1:1,000, AbCam); anti-CoREST (1:1,000, Millipore); anti-SRF (1:1,000, Cell Signaling Technology); anti-HDAC2 (1:1,000, Abcam); anti-NOVA1 (1:1,000, Sigma-Aldrich); anti-HA (1:500, Santa Cruz Biotechnology); anti-Transferrin Receptor (TfR) (1:1,000, Thermo Fisher Scientific); anti-Alpha Tubulin (1:40,000, Sigma-Aldrich); anti-PSD95 (1:1,000, Neuromab); anti-V5 (1:2,000, Millipore); anti-PCDH9 (1:500, Abcam), anti-PS1 (1:1,000, Proteintech). The following peroxidase-conjugated secondary antibodies were used: anti-rabbit (1:20,000, Jackson ImmunoResearch) and anti-mouse (1:2,000, GE Healthcare). Protein extracts from hiPSCs-derived neurons were separated by SDS-PAGE, transferred on nitrocellulose membrane (BioRad) and probed with antibodies against PCDH19 (1:30,000, Bethyl Laboratories) and GAPDH (1:1,000, Cell Signaling Technology). Proteins were then detected with Horseradish Peroxidase conjugated Goat anti-Rabbit (1:10,000, Invitrogen). Proteins were revealed with Super Signal West Femto ECL (Thermo Scientific) and visualized with ChemiDoc Touch Imaging system (BioRad).

ChIP

Chromatin immunoprecipitation (ChIP) experiments were performed as previously described (Rusconi et al., 2015). Briefly, mouse brain slices were incubated in 1% formaldehyde at RT for 15 min and transferred in 0.125 M glycine for 10 min. Afterwards, slices were homogenized in lysis buffer 3 (LB3) (10 mM Tris-HCl pH 8, 1 mM EDTA, 0.5 mM EGTA, 100 mM NaCl, 0.1% Na-deoxy-cholate, 0.5% N-lauroylsarcosine, protease inhibitors and 0.2 mM PMSF). Lysates were sonicated two times for 30 sec at 30% power (Bandelin Electronic Sonicator, Germany) to generate 200-500 base pair chromatin fragments and diluted in LB3 buffer plus 1X Triton-X100 and 1X PMSF. Immunoprecipitation was performed overnight by incubating lysates with anti-LSD1 (AbCam) or anti-PCDH19 (Bethyl Laboratories), while no antibody was used for control condition (mock). Protein G Dynabeads (Invitrogen) were added to samples and incubation proceeded for 2 h at 4°C. After immunoprecipitation input sample (mock supernatant) was kept apart. Beads were washed twice with low salt buffer (0.1% SDS, 2 mM EDTA, 1% Triton, 20 mM Tris-HCl pH 8, 150 mM NaCl), high salt buffer (0.1% SDS, 2 mM EDTA, 1% Triton, 20 mM Tris-HCl pH 8, 500 mM NaCl) and with once with TE buffer (10 mM Tris-HCl pH 8, 1 mM EDTA pH 8.0). Finally, beads were washed with TE-NaCl buffer (10 mM Tris-HCl, 1 mM EDTA, 50 mM NaCl). DNA was eluted with elution buffer (50 mM Tris-HCl pH 8, 10 mM EDTA pH 8.0, 1% SDS). Reversal of cross-linking was done ON at 65°C. Ribonuclease A (Sigma-Aldrich) and Proteinase K (Sigma-Aldrich, 1 h at 56°C) were added to eliminate RNA and proteins, respectively. DNA was recovered in 10 mM Tris-HCl pH 8. DNA levels were analyzed by quantitative real-time PCR (qRT-PCR) in both immunoprecipitated and input samples. RT-PCR was carried out using Power SYBR Green PCR Master Mix (Applied Biosystem) according to manufacturer's instructions and by using the primers in Table S9.

Three independent ChIP experiments were performed for each condition and all qRT-PCR analysis were performed in duplicate. Relative proportions of immunoprecipitated DNA were determined based on the threshold cycle (Ct) value for each PCR reaction. In order to control for variation between different ChIP fractions, a ΔCt value for each gene promoter analyzed was calculated by subtracting the Ct value of the input (Ct Input) from the Ct value of the immunoprecipitated sample (Ct antibody or Ct mock). Input DNA fraction represents only 1% of the total material. For this reason, the Ct input value was first adjusted for this dilution factor by subtracting 6.644 cycles (Log2 of 100). Data were then plotted as fold enrichment over mock.

RT-PCR

Primary neurons were transduced at DIV 8 with lentiviral vectors encoding CTF-V5 or PCDH19 shRNA and relative controls (empty cFUW vector and scramble, respectively). Total mRNA was extracted at DIV 12 using NucleoSpin RNA Kit (Macherey-Nagel) and cDNA was synthesized with iScript cDNA Synthesis Kit (Biorad) according to the manufacturer's instructions. RT-PCR was performed according to standard procedures (Longaretti et al., 2020) by using *RPSA* as endogenous control and the primers in Table S9.% of LSD1 splicing isoforms evaluation was performed as in (Zibetti et al., 2010).

hiPSCs-derived neurons were infected at DIV 1 with lentiviral vectors encoding PCDH19 shRNA and a relative control (scramble).

At DIV 21 or 35, as indicated, total mRNA was extracted using Nucleospin RNA Kit (Macherey-Nagel). cDNA was synthesized using iScript cDNA Synthesis Kit (Bio-Rad) according to the manufacturer's instructions. To measure mRNA expression, the primers in Table S9 were used.

QUANTIFICATION AND STATISTICAL ANALYSIS

Prism software (GraphPad) was used for the statistical analysis of data. Statistical significance was determined by using two-tailed Student's t test (pairwise comparisons) or one-way ANOVA (multiple comparisons). Tukey's or Dunnett's multiple comparison test

were used, as indicated. Data are presented as mean ± standard error of the mean (SEM). Differences were considered significance for $p < 0.05$. Asterisks indicate the following significance levels: *$p < 0.05$; **$p < 0.01$; ***$p < 0.001$. N represents the number of cells (Figures 1, 6C, 7D, and S1) or biological replicates on cell cultures lysates (Figures 2, 3, 4E, 5C, 6A, 6B, 6D, 6E, 7B, 7E, 7F, S2A–S2C, S3, and S5) or brain tissue homogenates (Figures 4F and S2D). Experiments on cultured neurons were done on at least three independent cultures. Statistical details (n, exact mean values, SEM, statistical tests and p values) can be found in the Tables S1–S8 and in figure legends.

Cell Reports, Volume *39*

Supplemental information

The epilepsy-associated protein PCDH19 undergoes NMDA receptor-dependent proteolytic cleavage and regulates the expression of immediate-early genes

Laura Gerosa, Sara Mazzoleni, Francesco Rusconi, Alessandra Longaretti, Elly Lewerissa, Silvia Pelucchi, Luca Murru, Serena Gea Giannelli, Vania Broccoli, Elena Marcello, Nael Nadif Kasri, Elena Battaglioli, Maria Passafaro, and Silvia Bassani

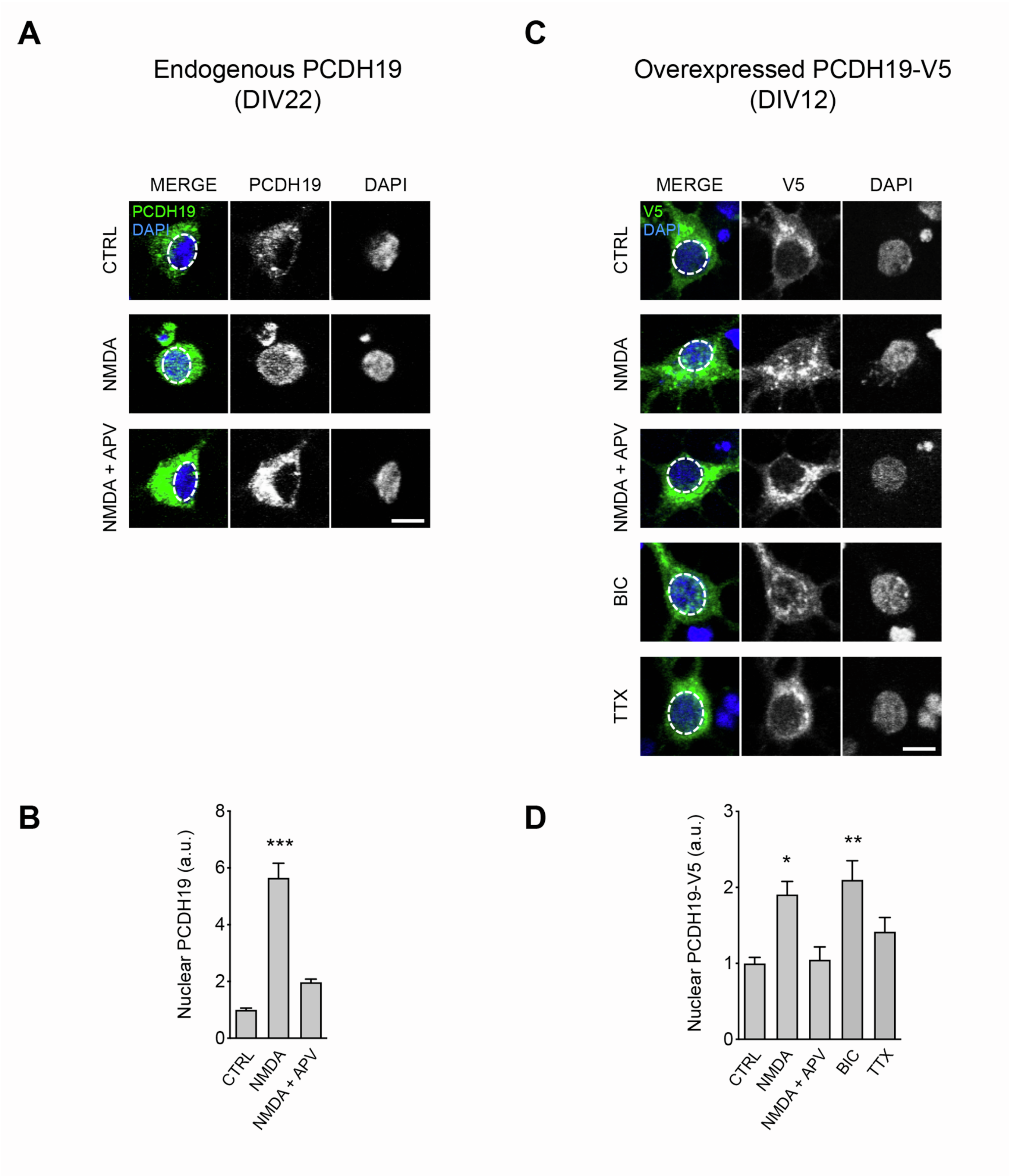
A
Endogenous PCDH19
(DIV22)
MERGE
PCDH19
DAPI
CTRL
NMDA
NMDA + APV
C
Overexpressed PCDH19-V5
(DIV12)
MERGE
V5
DAPI
CTRL
NMDA
NMDA + APV
BIC
TTX
B
Nuclear PCDH19 (a.u.)
***
0
2
4
6
8
CTRL
NMDA
NMDA + APV
D
Nuclear PCDH19-V5 (a.u.)
*
**
0
1
2
3
CTRL
NMDA
NMDA + APV
BIC
TTX

Figure S1. PCDH19 intracellular distribution changes following NMDAR activation in mature neurons expressing endogenous PCDH19 and developing neurons overexpressing PCDH19, Related to Figure 1

(A) Representative images of hippocampal neurons somata and nuclei at DIV 22 immunolabeled for PCDH19 and stained with DAPI, showing PCDH19 distribution after treatments with NMDA, NMDA + APV or vehicle (CTRL). Scale bar, 10 μm.

(B) Quantification of PCDH19 nuclear expression (n, CTRL 9, NMDA 11, NMDA + APV 9; CTRL vs. NMDA ***$p < 0.001$, one-way ANOVA and Dunnett's *post hoc* test). Data are presented as mean ± SEM.

(C) Representative images of hippocampal neurons somata and nuclei from neurons at DIV 12 previously transfected (DIV 4) with PCDH19-V5 and immunolabeled for V5 and stained with DAPI, showing PCDH19 distribution after treatments, as indicated. Scale bar, 10 μm.

(D) Quantification of PCDH19 nuclear expression (n, CTRL 10, NMDA 21, NMDA + APV 6, BIC 17, TTX 13; CTRL vs. NMDA *$p < 0.05$, CTRL vs. BIC **$p < 0.01$, one-way ANOVA and Dunnett's *post hoc* test). Data are presented as mean ± SEM.

Values are shown in Table S8.

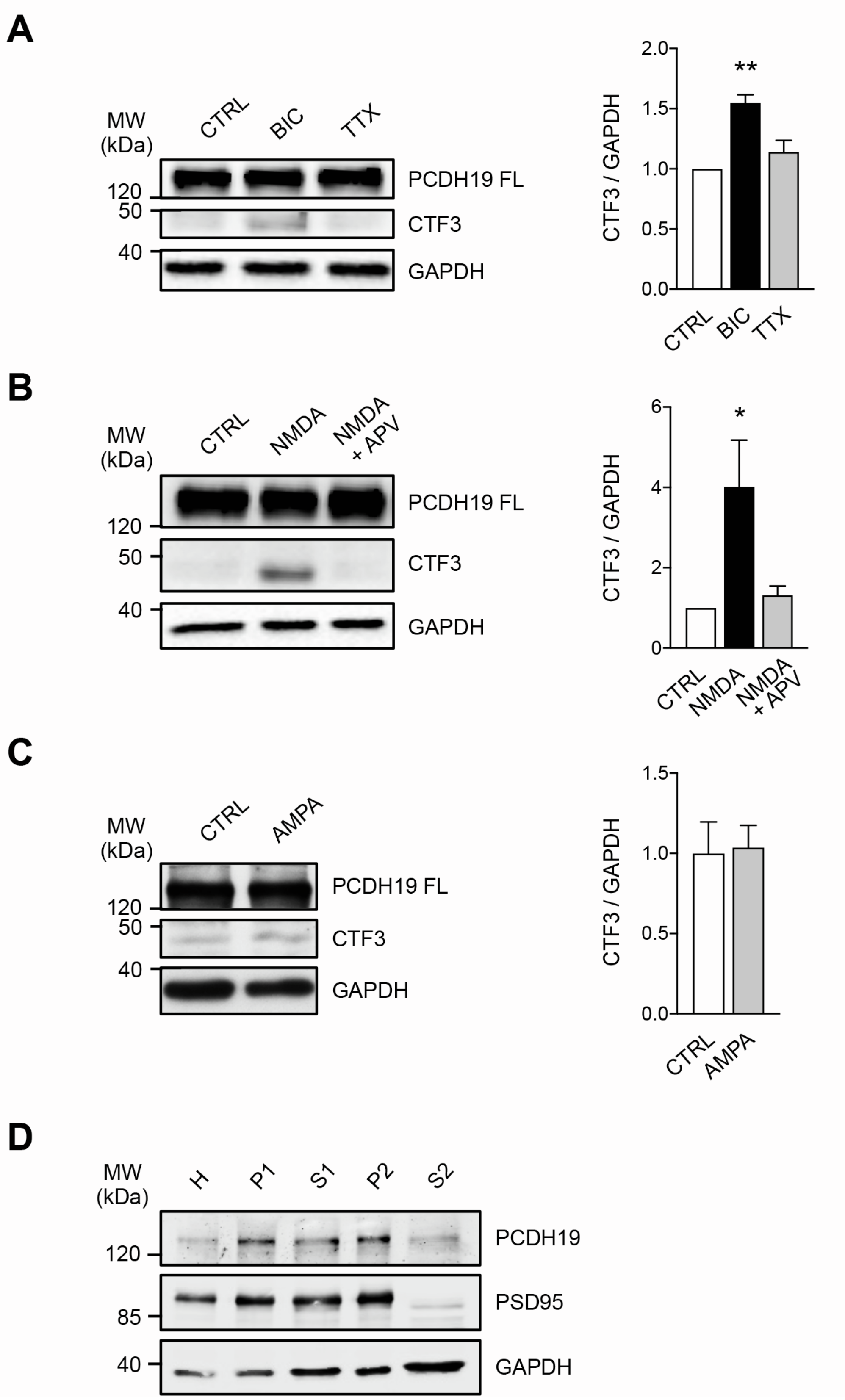

A
MW (kDa)
CTRL
BIC
TTX
PCDH19 FL
CTF3
GAPDH
120
50
40
CTF3 / GAPDH
2.0
1.5
1.0
0.5
0.0
**
CTRL
BIC
TTX
B
MW (kDa)
CTRL
NMDA
NMDA + APV
PCDH19 FL
CTF3
GAPDH
120
50
40
CTF3 / GAPDH
6
4
2
0
*
CTRL
NMDA
NMDA + APV
C
MW (kDa)
CTRL
AMPA
PCDH19 FL
CTF3
GAPDH
120
50
40
CTF3 / GAPDH
1.5
1.0
0.5
0.0
CTRL
AMPA
D
MW (kDa)
H
P1
S1
P2
S2
PCDH19
PSD95
GAPDH
120
85
40

Figure S2. PCDH19 proteolytic cleavage in hippocampal neurons and PCDH19 expression in crude synaptosomes, Related to Figure 2

(A) Representative western blots of primary hippocampal neurons at DIV 12 treated with vehicle (CTRL), BIC or TTX and quantification of PCDH19 CTF3 ($n = 3$; $^{**}p < 0.01$, one-way ANOVA and Dunnett's *post hoc* test). GAPDH is used as loading control. Data are presented as mean ± SEM.

(B) Western blots of primary hippocampal neurons at DIV 12 treated with vehicle (CTRL), NMDA or NMDA + APV and quantification of PCDH19 CTF3 ($n = 5$; $^{*}p < 0.05$, one-way ANOVA and Dunnett's *post hoc* test). Data are presented as mean ± SEM.

(C) Western blots of primary hippocampal neurons at DIV 12 treated with vehicle (CTRL) or AMPA and quantification of PCDH19 CTF3 ($n = 3$; $p > 0.05$, n.s., Student t test). Data are presented as mean ± SEM.

(D) Representative western blot of crude synaptosomes from rat brain (hippocampus and cortex). PSD95 is used as postsynaptic protein and GAPDH as loading control. H, total homogenate; P, pellet; S, supernatant. P2 fraction contains crude synaptosomes.

Values are shown in Table S8.

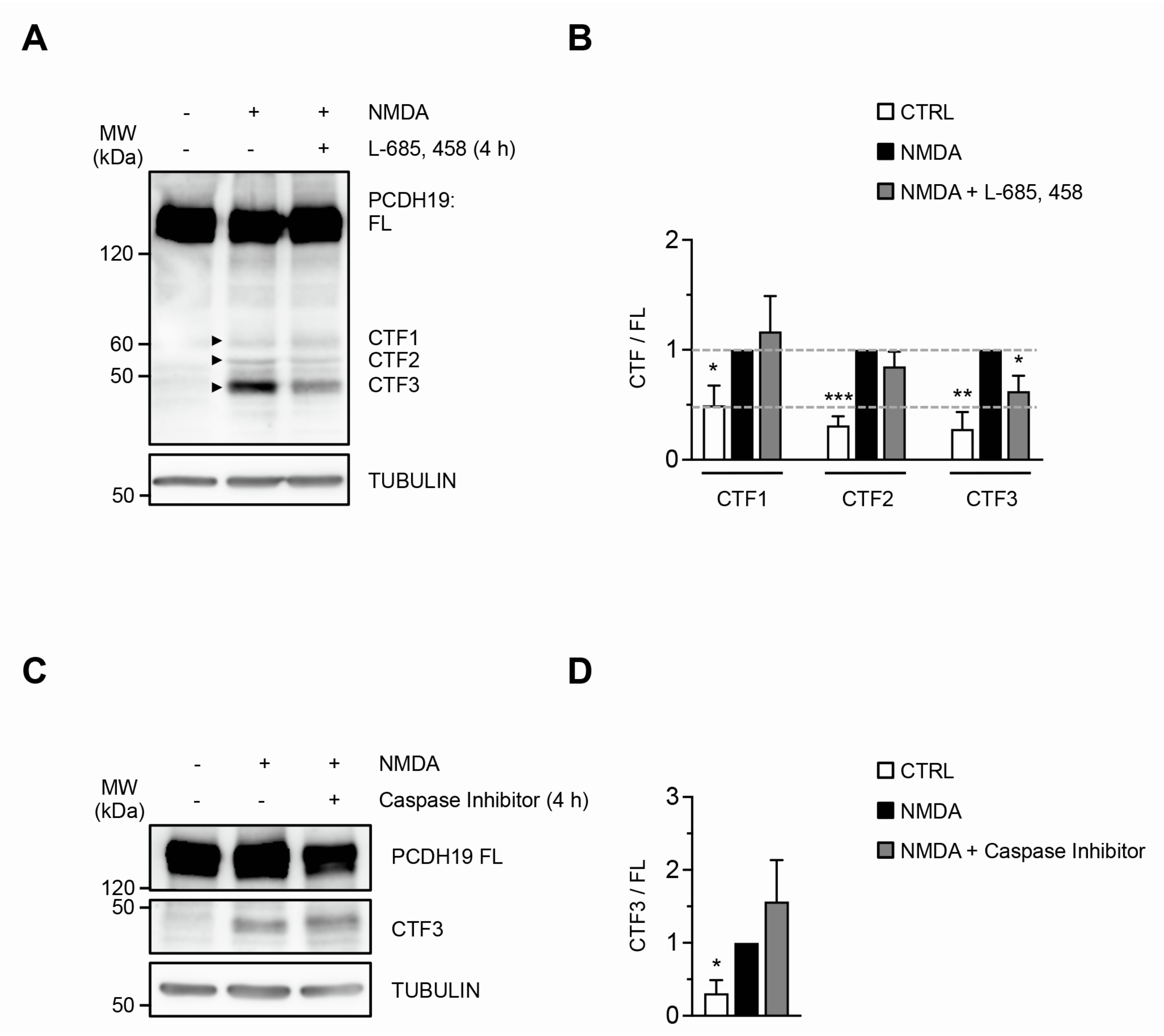

Figure S3. PCDH19 CTF3 level is reduced by gamma secretase inhibitor L-685, 458 but not by caspase inhibitor, Related to Figure 3

(A) Western blots of primary hippocampal neurons at DIV 12 treated with NMDA (6 min) NMDA + L-685, 458 (4 h) or vehicle (CTRL).

(B) Quantification of PCDH19 CTFs in western blots as in A (CTF1 / FL: n = 4; *p < 0.05, Student t test, CTRL compared with NMDA; CTF2 / FL: n = 4; ***p < 0.001, Student t test, CTRL compared with NMDA; CTF3 / FL: n = 4; **p < 0.01, Student t test, CTRL compared with NMDA; *p < 0.05, Student t test, NMDA + L-685, 458 compared with NMDA). Data are presented as mean ± SEM.

(C) Western blots of primary hippocampal neurons at DIV 12 treated with NMDA (6 min) NMDA + caspase inhibitor (4 h) or vehicle (CTRL).

(D) Quantification of PCDH19 CTF3 in western blots as in A (CTF3 / FL: n = 3; *p < 0.05, Student t test). Data are presented as mean ± SEM.

Values are shown in Table S8.

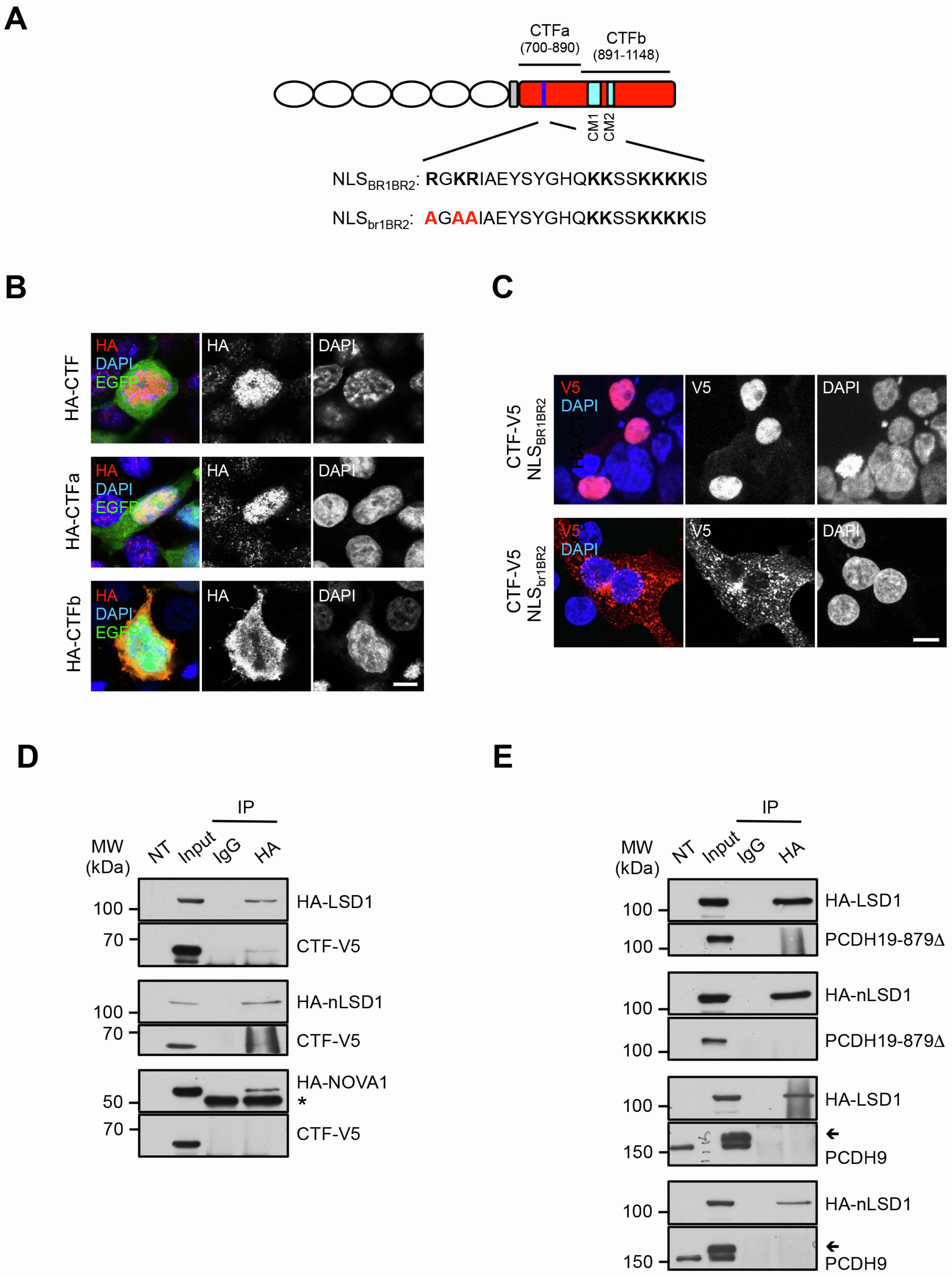
A
CTFa
(700-890)
CTFb
(891-1148)
CM1
CM2
NLSBR1BR2: RGKRIAEYSYGHQKKSSKKKKIS
NLSbr1BR2: AGAAIAEYSYGHQKKSSKKKKIS
B
HA-CTF
HA-CTFa
HA-CTFb
HA
DAPI
EGFP
C
CTF-V5 NLSBR1BR2
CTF-V5 NLSbr1BR2
V5
DAPI
D
E
IP
MW (kDa)
NT
Input
IgG
HA
HA-LSD1
CTF-V5
HA-nLSD1
HA-NOVA1
*
PCDH19-879Δ
PCDH9
100
70
50
150

Figure S4. PCDH19 CTF enters the nucleus and associates with LSD1 in HEK cells, Related to Figures 4 and 5

(A) Scheme of PCDH19 structure, showing PCDH19 intracellular C-terminus (CTF, red) with the nuclear localization signal (dark blue) and the conserved regions CM1 and CM2 (light blue). The amino acids composing the two basic regions of the bipartite NLS (NLS_{BR1BR2}, BR = basic region) are in bold, with the mutated amino acids in red.

(B) HEK cells transfected with HA-CTF, HA-CTFa and HA-CTFb together with pEGFP and stained with HA and DAPI. Scale bar, 10 µm.

(C) HEK cells transfected with wild type CTF-V5 (CTF-V5 NLS_{BR1BR2}) or CTF-V5 with mutated NLS (CTF-V5 NLS_{br1BR2}) and stained with V5 and DAPI. Scale bar, 10 µm.

(D) CoIP in HEK cells cotransfected with HA-LSD1/HA-nLSD1 and CTF-V5 or HA-NOVA1 and CTF-V5, as indicated. Input: 10 % of IP volume. IP: anti-IgG or anti-HA. Western blots probed for HA or V5. The star indicates IgG chains signal. NT, lysate from untransfected cells.

(E) CoIP in HEK cells cotransfected with HA-LSD1 and PCDH19-879Δ, HA-nLSD1 and PCDH19-879Δ, HA-LSD1 and PCDH9, or HA-nLSD1 and PCDH9, as indicated. Input: 10 % of IP volume. IP: anti-IgG or anti-HA. Western blots probed for HA, PCDH19 or PCDH9. The arrow indicates PCDH9 specific band, above of an aspecific signal. NT, lysate from untransfected cells.

Values are shown in Table S8.

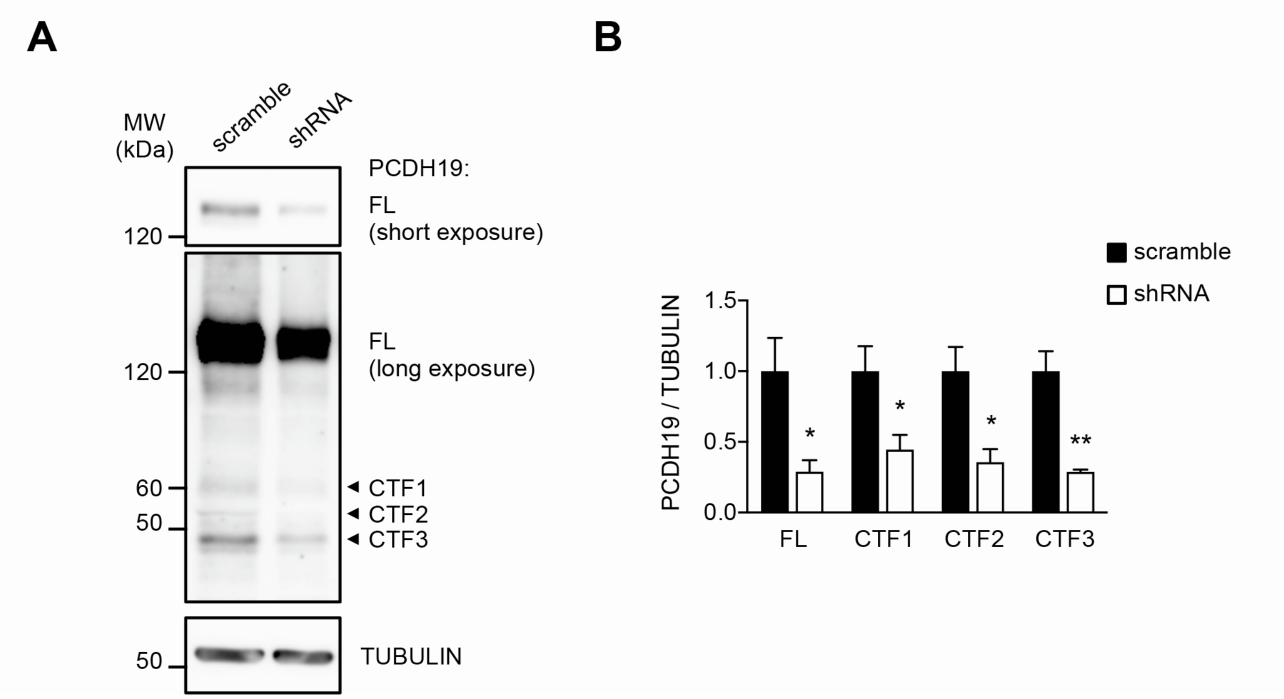

Figure S5. PCDH19 shRNA reduces PCDH19 FL and CTFs levels, Related to Figure 6
(A) Representative western blot of neurons transduced with PCDH19 shRNA (shRNA) or control shRNA (scramble). Neurons were treated with NMDA (50 μM, 6 min).
(B) Quantification of PCDH19 FL and CTFs expression in western blots as in A (PCDH19 / TUBULIN: n = 5; *$p < 0.05$, **$p < 0.01$, Student t test). Data are presented as mean ± SEM. Values are shown in Table S8.

Table S1. Mean values, SEM, number of samples and p values of data displayed in Figure 1, Related to Figure 1

**Fig. 1C**<br>**Dendritic PCDH19**	Mean	SEM	N	p value<br>(One-way ANOVA & Dunnett's *post hoc*)	
CTRL	1.000	0.033	17	CTRL vs.:	
BIC	0.764	0.045	23	BIC	$p = 0.004$ (**)
NMDA	0.805	0.036	26	NMDA	$p = 0.019$ (*)
NMDA + APV	1.017	0.060	26	NMDA + APV	$p > 0.05$ (n.s.)
**Fig. 1D**<br>**Nuclear PCDH19**	Mean	SEM	N	p value<br>(One-way ANOVA & Dunnett's *post hoc*)	
CTRL	1.000	0.062	152	CTRL vs.:	
BIC	1.808	0.127	20	BIC	$p < 0.001$ (***)
TTX	0.998	0.113	19	TTX	$p > 0.05$ (n.s.)
NMDA	1.740	0.123	47	NMDA	$p < 0.001$ (***)
NMDA + APV	1.151	0.114	31	NMDA + APV	$p > 0.05$ (n.s.)
SYN. NMDAR	1.529	0.150	61	SYN. NMDAR	$p < 0.001$ (***)
EXTRASYN. NMDAR	1.160	0.118	56	EXTRASYN. NMDAR	$p > 0.05$ (n.s.)

Table S2. Mean values, SEM, number of samples and p values of data displayed in Figure 2, Related to Figure 2

**Fig. 2A PCDH19 FL / GAPDH**	Mean	SEM	N	p value (One-way ANOVA & Tukey's *post hoc*)
CTRL	1.000	0.000	8	CTRL vs. NMDA $p < 0.01$ (**)
NMDA	0.573	0.085	8	CTRL vs. NMDA + APV $p > 0.05$ (n.s.)
NMDA + APV	0.909	0.104	7	NMDA vs. NMDA + APV $p < 0.05$ (*)
**PCDH19 FL / TfR**	Mean	SEM	N	p value (One-way ANOVA & Tukey's *post hoc*)
CTRL	1.000	0.000	4	CTRL vs. NMDA $p < 0.01$ (**)
NMDA	0.536	0.113	4	CTRL vs. NMDA + APV $p > 0.05$ (n.s.)
NMDA + APV	0.914	0.061	4	NMDA vs. NMDA + APV $p < 0.05$ (*)
**Fig. 2B PCDH19 (CTF / FL)**	Mean	SEM	N	p value (Student t test)
CTF1 (CTRL)	1.000	0.733	4	CTF1
CTF1 (NMDA)	2.507	1.460	4	(CTRL vs. NMDA) $p > 0.05$ (n.s.)
CTF2 (CTRL)	0.570	0.479	4	CTF2
CTF2 (NMDA)	3.329	2.000	4	(CTRL vs. NMDA) $p > 0.05$ (n.s.)
CTF3 (CTRL)	1.056	0.532	4	CTF3
CTF3 (NMDA)	8.789	2.312	4	(CTRL vs. NMDA) $p = 0.017$ (*)
CTF1 (CTRL) +MG	0.342	0.088	3	CTF1
CTF1 (NMDA) +MG	3.793	2.288	3	(CTRL vs. NMDA) $p > 0.05$ (n.s.)
CTF2 (CTRL) +MG	0.386	0.288	4	CTF2
CTF2 (NMDA) +MG	4.634	0.983	4	(CTRL vs. NMDA) $p = 0.006$ (**)
CTF3 (CTRL) +MG	1.254	0.815	4	CTF3
CTF3 (NMDA) +MG	14.148	4.906	3	(CTRL vs. NMDA) $p = 0.028$ (*)
**Fig. 2C PCDH19 FL / GAPDH**	Mean	SEM	N	p value (One-way ANOVA & Dunnett's *post hoc*)
CTRL (C)	1.000	0.000	4	CTRL vs.
3 min	1.031	0.102	4	3 min $p > 0.05$ (n.s.)
6 min	0.868	0.124	4	6 min $p > 0.05$ (n.s.)
10 min	0.684	0.091	4	10 min $p < 0.05$ (*)
20 min	0.693	0.048	4	20 min $p > 0.05$ (n.s.)
30 min	0.522	0.049	4	30 min $p < 0.01$ (**)
**CTF3 / GAPDH**	Mean	SEM	N	p value (One-way ANOVA & Dunnett's *post hoc*)
CTRL (C)	1.000	0.000	4	CTRL vs.

3 min	11.405	2.325	4	3 min	p > 0.05 (n.s.)
6 min	19.110	3.343	4	6 min	p < 0.05 (*)
10 min	23.769	3.565	4	10 min	p < 0.01 (**)
20 min	39.834	5.825	4	20 min	p < 0.001 (***)
30 min	23.966	6.653	4	30 min	p < 0.01 (**)
**Fig. 2D CTF2 / FL (NMDA / CTRL)**	Mean	SEM	N	p value (Student t test)	
H	1.654	0.481	3		
TIF	3.265	0.153	3	H vs. TIF	p = 0.032 (*)

Table S3. Mean values, SEM, number of samples and p values of data displayed in Figure 3, Related to Figure 3

**Fig. 3B PCDH19 (CTF1 / FL)**	Mean	SEM	N	p value (Student t test)	
CTRL	0.169	0.040	4	CTRL vs. NMDA	$p < 0.001$ (***)
NMDA	1.000	0.000	4-5		
NMDA + GM6001	0.186	0.058	5	NMDA + GM6001 vs. NMDA	$p < 0.001$ (***)
NMDA + GI254023X	0.152	0.059	4	NMDA + GI254023X vs. NMDA	$p < 0.001$ (***)
NMDA + DAPT	1.066	0.064	5	NMDA + DAPT vs. NMDA	$p > 0.05$ (n.s.)
**PCDH19 (CTF2 / FL)**	Mean	SEM	N	p value (Student t test)	
CTRL	0.095	0.053	5	CTRL vs. NMDA	$p < 0.001$ (***)
NMDA	1.000	0.000	5-6		
NMDA + GM6001	0.110	0.027	6	NMDA + GM6001 vs. NMDA	$p < 0.001$ (***)
NMDA + GI254023X	0.078	0.031	5	NMDA + GI254023X vs. NMDA	$p < 0.001$ (***)
NMDA + DAPT	1.002	0.087	6	NMDA + DAPT vs. NMDA	$p > 0.05$ (n.s.)
**PCDH19 (CTF3 / FL)**	Mean	SEM	N	p value (Student t test)	
CTRL	0.177	0.123	4	CTRL vs. NMDA	$p < 0.001$ (***)
NMDA	1.000	0.000	3-8		
NMDA + GM6001	0.689	0.081	6	NMDA + GM6001 vs. NMDA	$p = 0.003$ (**)
NMDA + GI254023X	0.560	0.153	3	NMDA + GI254023X vs. NMDA	$p = 0.045$ (*)
NMDA + DAPT	0.809	0.045	8	NMDA + DAPT vs. NMDA	$p < 0.001$ (***)
**Fig. 3D PCDH19 (CTF1 / FL)**	Mean	SEM	N	p value (Student t test)	
CTRL	0.6534	0.116	8	CTRL vs. NMDA	$p = 0.010$ (*)
NMDA	1.000	0.000	4-8		
NMDA + GI254023X 4h	0.340	0.065	5	NMDA + GI254023X vs. NMDA 4h	$p < 0.001$ (***)
NMDA + DAPT 4h	1.625	0.359	6	NMDA + DAPT vs. NMDA 4h	$p > 0.05$ (n.s.)
NMDA + DAPT + GI254023X 4h	0.880	0.188	5	NMDA + DAPT + GI254023X vs. NMDA 4h	$p > 0.05$ (n.s.)
NMDA + GI254023X 24h	0.361	0.052	4	NMDA + GI254023X vs. NMDA 24h	$p < 0.001$ (***)
NMDA + DAPT 24h	3.693	0.582	7	NMDA + DAPT vs. NMDA 24h	$p < 0.001$ (***)
NMDA + DAPT + GI254023X 24h	0.934	0.281	6	NMDA + DAPT + GI254023X vs. NMDA 24h	$p > 0.05$ (n.s.)
**PCDH19**	Mean	SEM	N	p value	

(CTF2 / FL)				(Student t test)	
CTRL	0.381	0.103	8	CTRL vs. NMDA	p < 0.001 (***)
NMDA	1.000	0.000	5-9		
NMDA + GI254023X 4h	0.112	0.057	5	NMDA + GI254023X vs. NMDA 4h	p < 0.001 (***)
NMDA + DAPT 4h	1.861	0.480	5	NMDA + DAPT vs. NMDA 4h	p > 0.05 (n.s.)
NMDA + DAPT + GI254023X 4h	0.414	0.204	5	NMDA + DAPT + GI254023X vs. NMDA 4h	p = 0.020 (*)
NMDA + GI254023X 24h	0.384	0.173	6	NMDA + GI254023X vs. NMDA 24h	p = 0.001 (**)
NMDA + DAPT 24h	3.618	0.671	9	NMDA + DAPT vs. NMDA 24h	p = 0.021 (*)
NMDA + DAPT + GI254023X 24h	1.059	0.361	8	NMDA + DAPT + GI254023X vs. NMDA 24h	p > 0.05 (n.s.)
**PCDH19 (CTF3 / FL)**	Mean	SEM	N	p value (Student t test)	
CTRL	0.302	0.081	9	CTRL vs. NMDA	p < 0.001 (***)
NMDA	1.000	0.000	4-9		
NMDA + GI254023X 4h	0.674	0.086	4	NMDA + GI254023X 4h vs. NMDA	p = 0.009 (**)
NMDA + DAPT 4h	0.677	0.079	5	NMDA + DAPT 4h vs. NMDA	p = 0.003 (**)
NMDA + DAPT + GI254023X 4h	0.542	0.120	4	NMDA + DAPT + GI254023X 4h vs. NMDA	p = 0.009 (**)
NMDA + GI254023X 24h	0.738	0.061	5	NMDA + GI254023X 24h vs. NMDA	p = 0.002 (**)
NMDA + DAPT 24h	1.471	0.247	9	NMDA + DAPT 24h vs. NMDA	p > 0.05 (n.s.)
NMDA + DAPT + GI254023X 24h	1.282	0.250	8	NMDA + DAPT + GI254023X 24h vs. NMDA	p > 0.05 (n.s.)
**Fig. 3E PS1 FL / GAPDH**	Mean	SEM	N	p value (One-way ANOVA & Tukey's *post hoc*)	
NMDA	1.000	0.000	3	NMDA + DAPT 4h vs. NMDA	p > 0.05 (n.s.)
NMDA + DAPT 4h	0.994	0.055	3	NMDA + DAPT 24h vs. NMDA	p > 0.05 (n.s.)
NMDA + DAPT 24h	0.911	0.176	3	NMDA + DAPT 24h vs. NMDA + DAPT 4h	p > 0.05 (n.s.)
**PS1 CTF / GAPDH**	Mean	SEM	N	p value (One-way ANOVA & Tukey's *post hoc*)	
NMDA	1.000	0.000	3	NMDA + DAPT 4h vs. NMDA	p > 0.05 (n.s.)
NMDA + DAPT 4h	1.134	0.059	3	NMDA + DAPT 24h vs. NMDA	p < 0.001 (***)
NMDA + DAPT 24h	1.814	0.109	3	NMDA + DAPT 24h vs. NMDA + DAPT 4h	p = 0.001 (**)

Table S4. Mean values, SEM, number of samples and p values of data displayed in Figure 4, Related to Figure 4

**Fig. 4E**<br>CTF3 (P2 / H)	Mean	SEM	N	p value<br>(One-way ANOVA & Tukey's *post hoc*)	
CTRL	1.000	0.000	3	CTRL vs. NMDA	p > 0.05 (n.s.)
NMDA	2.295	0.505	3	CTRL vs. NMDA + APV	p > 0.05 (n.s.)
NMDA + APV	0.613	0.275	3	NMDA vs. NMDA + APV	p = 0.027 (*)
**Fig. 4F**<br>CTF3 / GAPDH	Mean	SEM	N	p value<br>(Student t test)	
H (CTRL)	1.000	0.061	5	H:	
H (PILO)	2.092	0.574	3	CTRL vs. PILO	p = 0.043 (*)
C (CTRL)	0.304	0.081	5	C:	
C (PILO)	1.390	0.286	3	CTRL vs. PILO	p = 0.003 (**)
CTF3 / PCDH19 FL	Mean	SEM	N	p value<br>(Student t test)	
H (CTRL)	1.000	0.259	5	H:	
H (PILO)	3.059	1.397	3	CTRL vs. PILO	p > 0.05 (n.s.)
N-M (CTRL)	0.639	0.027	4	N-M:	
N-M (PILO)	2.698	0.847	3	CTRL vs. PILO	p = 0.033 (*)

Table S5. Mean values, SEM, number of samples and p values of data displayed in Figure 5, Related to Figure 5

**Fig. 5C**<br>**LSD1 ChIP**<br>**(% of the input)**	Mean	SEM	N	p value<br>(Student t test)
Nr4a1 mock IP	0.053	0.015	4	Nr4a1:<br>IP mock vs. LSD1 p = 0.008 (**)
Nr4a1 LSD1 IP	0.137	0.015	4	
c-Fos mock IP	0.051	0.014	4	c-Fos:<br>IP mock vs. LSD1 p = 0.001 (**)
c-Fos LSD1 IP	0.334	0.051	4	
Npas4 mock IP	0.018	0.008	4	Npas4:<br>IP mock vs. LSD1 p = 0.017 (*)
Npas4 LSD1 IP	0.058	0.008	4	
**CTF ChIP**<br>**(% of the input)**	Mean	SEM	N	p value<br>(Student t test)
Nr4a1 mock IP	0.042	0.013	3	Nr4a1:<br>IP mock vs. CTF p = 0.004 (**)
Nr4a1 CTF IP	0.204	0.025	3	
c-Fos mock IP	0.045	0.018	3	c-Fos:<br>IP mock vs. CTF p = 0.017 (*)
c-Fos CTF IP	0.253	0.049	3	
Npas4 mock IP	0.010	0.005	3	Npas4:<br>IP mock vs. CTF p = 0.012 (*)
Npas4 CTF IP	0.088	0.017	3	

Table S6. Mean values, SEM, number of samples and p values of data displayed in Figure 6, Related to Figure 6

**Fig. 6A**<br>RT-PCR<br>(rel. mRNA level)	Mean	SEM	N	p value<br>(Student t test)
Nr4a1 (CTRL)	0.685	0.180	3	Nr4a1:
Nr4a1 (CTF-V5)	0.120	0.025	3	CTRL vs. CTF-V5 $p = 0.036$ (*)
c-Fos (CTRL)	0.791	0.181	3	c-Fos:
c-Fos (CTF-V5)	0.143	0.022	3	CTRL vs. CTF-V5 $p = 0.023$ (*)
Egr1 (CTRL)	1.008	0.073	4	Egr1:
Egr1 (CTF-V5)	0.991	0.120	4	CTRL vs. CTF-V5 $p > 0.05$ (n.s.)
Cyr61 (CTRL)	2.253	0.979	3	Cyr61:
Cyr61 (CTF-V5)	1.284	0.715	3	CTRL vs. CTF-V5 $p > 0.05$ (n.s.)
Npas4 (CTRL)	1.002	0.314	3	Npas4:
Npas4 (CTF-V5)	0.080	0.028	3	CTRL vs. CTF-V5 $p = 0.043$ (*)
**Fig. 6B**<br>RT-PCR<br>(rel. mRNA level)	Mean	SEM	N	p value<br>(Student t test)
Nr4a1 (scramble)	0.818	0.094	8	Nr4a1:
Nr4a1 (shRNA)	2.394	0.645	7	scramble vs. shRNA $p = 0.022$ (*)
c-Fos (scramble)	0.659	0.122	8	c-Fos:
c-Fos (shRNA)	0.680	0.163	7	scramble vs. shRNA $p > 0.05$ (n.s.)
Egr1 (scramble)	1.010	0.064	6	Egr1:
Egr1 (shRNA)	1.582	0.153	6	scramble vs. shRNA $p = 0.006$ (**)
Cyr61 (scramble)	0.171	0.046	7	Cyr61:
Cyr61 (shRNA)	0.182	0.059	6	scramble vs. shRNA $p > 0.05$ (n.s.)
Npas4 (scramble)	1.170	0.202	8	Npas4:
Npas4 (shRNA)	2.898	0.857	6	scramble vs. shRNA $p = 0.044$ (*)
**Fig. 6C**<br>NR4A1 intensity	Mean	SEM	N	p value<br>(One way ANOVA and Tukey's *post hoc*)
scramble (vehicle)	1.000	0.080	58	scramble (veh.) vs. :<br>scramble (NMDA) $p < 0.001$ (***)
scramble (NMDA)	1.644	0.173	32	CTF-V5 NLS$_{BR1BR2}$<br>(NMDA) $p > 0.05$ (n.s.)
CTF-V5 NLS$_{BR1BR2}$<br>(NMDA)	1.080	0.172	20	CTF-V5 NLS$_{br1BR2}$<br>(NMDA) $p = 0.024$ (*)
CTF-V5 NLS$_{br1BR2}$<br>(NMDA)	1.620	0.124	16	shRNA (veh.) $p = 0.006$ (**)
shRNA (vehicle)	1.535	0.116	35	Other comparisons $p > 0.05$ (n.s.)
scramble (DAPT)	1.000	0.055	15	scramble (DAPT) vs. :
shRNA + PCDH19	1.152	0.084	17	shRNA + PCDH19 $p > 0.05$ (n.s.)
shRNA + PCDH19<br>(DAPT)	1.397	0.066	14	shRNA + PCDH19 (DAPT) $p = 0.001$ (**)<br>shRNA + PCDH19 vs. :<br>shRNA + PCDH19 (DAPT) $p > 0.05$ (n.s.)

**Fig. 6D**<br>**% nLSD1**	Mean	SEM	N	p value<br>(Student t test)
CTRL	62.240	1.372	6	
CTF	64.669	2.174	6	CTRL vs. CTF p > 0.05 (n.s.)
scramble	55.931	0.435	6	
shRNA	70.165	0.578	6	scramble vs. shRNA p < 0.001 (***)
**Fig. 6E**<br>**NOVA1 expression**	Mean	SEM	N	p value<br>(One way ANOVA and Dunnett's *post hoc*)
scramble	1.018	0.029	4	scramble vs.:
shRNA	1.934	0.186	4	shRNA p = 0.004 (**)
rescue	0.884	0.214	3	rescue p > 0.05 (n.s.)

Table S7. Mean values, SEM, number of samples and p values of data displayed in Figure 7, Related to Figure 7

**Fig. 7B**<br>PCDH19 (CTF/FL)	Mean	SEM	N	p value<br>(One-way ANOVA & Tukey's post hoc)
CTRL	1.000	0.143	3	CTRL vs. NMDA p = 0.001 (**)
NMDA	6.434	0.941	3	CTRL vs. NMDA + APV p > 0.05 (n.s.)
NMDA + APV	1.530	0.381	3	NMDA vs. NMDA + APV p = 0.002 (**)
**Fig. 7D**<br>Nuclear PCDH19	Mean	SEM	N	p value<br>(Student t test)
CTRL	1.000	0.057	12	
NMDA	3.196	0.513	12	CTRL vs. NMDA p < 0.001 (***)
**Fig. 7E**<br>RT-PCR<br>(rel. mRNA level)	Mean	SEM	N	p value<br>(Student t test)
PCDH19 (scramble)	1.000	0.000	3	PCDH19:
PCDH19 (shRNA)	0.302	0.050	3	scramble vs. shRNA p < 0.001 (***)
**Fig. 7F**<br>RT-PCR<br>(rel. mRNA level)	Mean	SEM	N	p value<br>(Student t test)
NR4A1 (scramble)	0.8632	0.071	7	NR4A1:
NR4A1 (shRNA)	0.8709	0.149	8	scramble vs. shRNA p > 0.05 (n.s.)
c-FOS (scramble)	0.8834	0.146	7	c-FOS:
c-FOS (shRNA)	1.134	0.166	8	scramble vs. shRNA p > 0.05 (n.s.)
EGR1 (scramble)	0.8473	0.094	5	EGR1:
EGR1 (shRNA)	2.400	0.536	5	scramble vs. shRNA p = 0.03 (*)
CYR61 (scramble)	0.8835	0.079	5	CYR61:
CYR61 (shRNA)	1.219	0.289	6	scramble vs. shRNA p > 0.05 (n.s.)
NPAS4 (scramble)	1.039	0.039	4	NPAS4:
NPAS4 (shRNA)	4.549	0.384	4	scramble vs. shRNA p = 0.001 (**)

Table S8. Mean values, SEM, number of samples and p values of data displayed in Figures S1, S2, S3, and S5, Related to Figures 1, S1, 2, S2, 3, S3, 6 and S5

**Fig. S1B**<br>Nuclear PCDH19	Mean	SEM	N	p value<br>(One-way ANOVA & Dunnett's *post hoc*)
CTRL	1.000	0.060	9	CTRL vs.:
NMDA	5.649	0.512	11	NMDA $p < 0.001$ (***)
NMDA + APV	1.970	0.114	9	NMDA + APV $p > 0.05$ (n.s.)
**Fig. S1D**<br>Nuclear PCDH19-V5	Mean	SEM	N	p value<br>(One-way ANOVA & Dunnett's *post hoc*)
CTRL	1.000	0.079	10	CTRL vs.:
NMDA	1.905	0.173	21	NMDA $p = 0.011$ (*)
NMDA + APV	1.048	0.169	6	NMDA + APV $p > 0.05$ (n.s.)
BIC	2.098	0.252	17	BIC $p = 0.002$ (**)
TTX	1.415	0.186	13	TTX $p > 0.05$ (n.s.)
**Fig. S2A**<br>CTF3 / GAPDH	Mean	SEM	N	p value<br>(One way ANOVA and Dunnett's *post hoc*)
CTRL	1.000	0.000	3	CTRL vs.:
BIC	1.545	0.069	3	BIC $p = 0.002$ (**)
TTX	1.140	0.095	3	TTX $p > 0.05$ (n.s.)
**Fig. S2B**<br>CTF3 / GAPDH	Mean	SEM	N	p value<br>(One way ANOVA and Dunnett's *post hoc*)
CTRL	1.000	0.000	5	CTRL vs.:
NMDA	4.008	1.163	5	NMDA $p = 0.016$ (*)
NMDA + APV	1.314	0.234	5	NMDA + APV $p > 0.05$ (n.s.)
**Fig. S2C**<br>CTF3 / GAPDH	Mean	SEM	N	p value<br>(Student t test)
CTRL	1.000	0.196	3	CTRL vs.
AMPA	1.036	0.138	3	AMPA $p > 0.05$ (n.s.)
**Fig. S3B**<br>**PCDH19 (CTF1 / FL)**	Mean	SEM	N	p value<br>(Student t test)
CTRL	0.494	0.180	4	NMDA vs. :
NMDA	1.000	0.000	4	CTRL $p = 0.031$ (*)
NMDA + L-685, 458	1.167	0.321	4	NMDA + L-685, 458 $p > 0.05$ (n.s.)
**PCDH19 (CTF2 / FL)**	Mean	SEM	N	p value<br>(Student t test)
CTRL	0.312	0.083	4	NMDA vs. :
NMDA	1.000	0.000	4	CTRL $p < 0.001$ (***)
NMDA + L-685, 458	0.848	0.134	4	NMDA + L-685, 458 $p > 0.05$ (n.s.)

**PCDH19 (CTF3 / FL)**	Mean	SEM	N	p value (Student t test)
CTRL	0.280	0.154	4	NMDA vs. :
NMDA	1.000	0.000	4	CTRL p = 0.003 (**)
NMDA + L-685, 458	0.623	0.140	4	NMDA + L-685, 458 p = 0.036 (*)
**Fig. S3D PCDH19 (CTF3 / FL)**	Mean	SEM	N	p value (Student t test)
CTRL	0.308	0.180	3	NMDA vs. :
NMDA	1.000	0.000	3	CTRL p = 0.018 (*)
NMDA + Caspase Inh.	1.566	0.568	3	NMDA + Caspase Inh. p > 0.05 (n.s.)
**Fig. S5B PCDH19 / TUBULIN**	Mean	SEM	N	p value (Student t test)
FL (scramble)	1.000	0.235	5	
FL (shRNA)	0.289	0.080	5	scramble vs. shRNA p = 0.021 (*)
CTF1 (scramble)	1.000	0.176	5	
CTF1 (shRNA)	0.445	0.103	5	scramble vs. shRNA p = 0.026 (*)
CTF2 (scramble)	1.000	0.171	5	
CTF2 (shRNA)	0.355	0.092	5	scramble vs. shRNA p = 0.010 (*)
CTF3 (scramble)	1.000	0.141	5	
CTF3 (shRNA)	0.287	0.017	5	scramble vs. shRNA p = 0.001 (**)

Table S9. Oligonucleotides sequences used for data in Figures 4, S4, 5, 6, and 7, Related to Figures 4, S4, 5, 6, and 7

**Figs. 4, S4 Name:**	**Primers for mutagenesis Sequence (5' - 3'):**
Fw	GGAGAAAGTGAGCCTAGCGGGAGCGGCAATTGCTGAGTACTCCTATGG
Rev	CCATAGGAGTACTCAGCAATTGCCGCTCCCGCTAGGCTCACTTCTCC
**Fig. 5 Name:**	**Primers for ChIP experiments Sequence (5' - 3'):**
Nr4a1, Fw	GTCACGGAGCGCTTAAGATGT
Nr4a1, Rev	TCCGCTCCGCAGTCCTTCTA
c-Fos, Fw	TCAGAGTTGGCTGCAGCCGGC
c-Fos, Rev	GCGTGTAGGATTTCGGAGATG
Npas4, Fw	AAAGGGTCTTGGGTAGGTGC
Npas4, Rev	CCTCCGCACAGCTCTAGAAAA
**Fig. 6 Name:**	**Primers for RT-PCR Sequence (5' - 3'):**
RPSA, Fw	ACCCAGAGGAGATTGAGAAGG
RPSA, Rev	TGGGGAACTGCTGAATGGGC
Nr4a1, Fw	GAAGCTCATCTTCTGCTCAGG
Nr4a1, Rev	CCATGTCGATCAGTGATGAGG
c-Fos, Fw	TCAGAGTTGGCTGCAGCCGGC
c-Fos, Rev	GCGTGTAGGATTTCGGAGATG
Egr1, Fw	TTCAATCCTCAAGGGGAGC
Egr1, Rev	AACCGGGTAGTTTGGCTGGGA
Cyr61, Fw	AACGAGGACTGCAGCAAAACG
Cyr61, Rev	TCTGAGTGAGCTCTGCAGATC
Npas4, Fw	GGTGGTGAGACTTCAAGCCAA
Npas4, Rev	TCCGTGTCACTGATAGGGTAG
**Fig. 7 Name:**	**Primers for RT-PCR Sequence (5' - 3'):**
PCDH19, Fw	AACGATGTGCTGAACACCAG
PCDH19, Rev	GCATCCAGCACCTGTCAGAGT
NR4A1, Fw	CACAGCTTGCTTGTCGATGTC
NR4A1, Rev	ATGCCGGTCGGTGATGAG
c-FOS, Fw	GCGTTGTGAAGACCATGACAG
c-FOS, Rev	TCTAGTTGGTCTGTCTCCGCT
EGR1, Fw	ACCTGACCGCAGAGTCTTTTC
EGR1, Rev	GTGGTTTGGCTGGGGTAACT
CYR61, Fw	GAAGCGGCTCCCTGTTTTTG
CYR61, Rev	CGGGTTTCTTTCACAAGGCG
NPAS4, Fw	CTGCATCTACACTCGCAAGG
NPAS4, Rev	GCCACAATGTCTTCAAGCTCT
